# New adjusted missing value imputation in multiple regression with simple random sampling and rank set sampling methods

**DOI:** 10.1371/journal.pone.0316641

**Published:** 2025-03-17

**Authors:** Juthaphorn Sinsomboonthong, Saichon Sinsomboonthong

**Affiliations:** 1 Department of Statistics, Faculty of Science, Kasetsart University, Bangkok, Thailand; 2 Department of Statistics, School of Science, King Mongkut’s Institute of Technology Ladkrabang, Bangkok, Thailand; Max Planck Institute for Solid State Research, GERMANY

## Abstract

This research compared the efficiency of several adjusted missing value imputation methods in multiple regression analysis. The four imputation methods were the following: regression-ratio quartile1,3 (R-RQ1,3) imputation of Al-Omari, Jemain and Ibrahim; adjusted regression-chain ratio quartile1,3 (AR-CRQ1,3) imputation of Kadilar and Cinji; adjusted regression-multivariate ratio quatile1,3 (AR-MRQ1,3) imputation of Feng, Ni, and Zou; and adjusted regression-multivariate chain ratio quartile1,3 (AR-MCRQ1,3) imputation of Lu for each simple random sampling (SRS) and rank set sampling (RSS). The performance measures mean square error (MSE) and mean absolute percentage error (MAPE). The study showed that the AR-MRQ1 method with SRS provided the minimum mean square error for small error variance. However, the AR-MCRQ3 provided the minimum mean square error for a large error variance. Considering all error variance in mean absolute percentage error, the AR-MCRQ1 provided the minimum mean absolute percentage error. The AR-MRQ1 method with RSS provided the minimum mean square error for a small error variance. However, the AR-MCRQ3 provided the minimum mean square error for medium and large error variance. Regarding the mean absolute percentage error measure, the AR-MRQ1 provided the minimum mean absolute percentage error for a small error variance. However, the AR-MCRQ1 provided the minimum mean absolute percentage error for medium and large error variance. For both SRS and RSS, AR-MCRQ1 was the best method for missing value imputation in multiple regression analysis, followed by AR-MCRQ3. Moreover, the RSS estimators provided smaller MSE and MAPE than the SRS estimators. Therefore, the RSS estimators were more efficient than the SRS estimators.

## 1. Introduction

Multiple regression analysis is a study of the relationship between many independent variables and a dependent variable to determine which independent variables can estimate or explain the variation of the dependent variable. The regression model is a matrix of *✓*. Estimation of the parameters often causes problems. Information or data can be incomplete, and significant data values may be missing, resulting from erratic data storage or transfer tools or limitations of the technology, leading to an inability to utilize the data [1] fully. To use multiple regression analysis effectively, the data must be complete. For this reason, some researchers may not include the data of some variables and make an analysis only on a smaller set of full data, which may not give a meaningful result of a biased estimate or a high standard error. The discarded data can differ significantly from the remaining data [2], making the estimation unreliable. Therefore, a reasonable estimation of missing values is fundamental.

Simple random sampling (SRS) is a sampling of a population of size N, where each possible sample of size n had the same probability of being selected. The sample obtained at such sampling is called a simple random sample [2]. Rank set sampling (RSS) was introduced by McIntyre (1952) for estimating mean pasture and forage yields as a more efficient and cost-effective method than the commonly used simple random sampling in situations where the visual ordering of the sample units can be done quickly, the exact measurement of the units is difficult and expensive [3]. Takahasi and Wakimoto (1968) provided the necessary mathematical theory of RSS [4]. Samawi and Muttlak (1996) studied using RSS to estimate the population ratio and suggested the RSS estimator of the population ratio [5]. The data sampled by SRS and RSS may be quite different, and the various proposed missing value imputation methods investigated in this study may give different results from the data obtained by the two sampling methods.

Three assumptions are discussed in the context of missing imputation, which also forms the basis of later simulations: missing completely at random (MCAR), missing at random (MAR), and missing not at random (MNAR) [6–8].

It is essential to understand why the data are missing. Graham et al. (2003) in [7] described that missing data can informally be thought of as being caused in some combination of three ways: random processes, processes that are measured, and processes that are not measured. Modern missing data methods generally work well for the first two causes, but not for the last. More formally, missing data assumptions are commonly described as falling into one of three categories, defined by [9]: First, data can be “Missing Completely at Random”, or MCAR. When data are MCAR, missing cases are no different than non-missing cases in the analysis. Thus, these cases can be thought of as randomly missing from the data, and the only real penalty for failing to account for missing data is loss of power. Second, data can be “Missing at Random”, or MAR. In this case, missing data depends on known values and thus is described entirely by variables observed in the data set. Accounting for the values that “cause” the missing data will produce unbiased results in an analysis. Third, data can be missing in an unmeasured fashion, termed “nonignorable” (also called “Missing Not at Random” (MNAR) and “Not Missing at Random” (NMAR)). Since the missing data depends on events or items that the researcher has not measured, it cannot be adquately attributed without measuring some related variables.

Missing data can frequently occur in a longitudinal data analysis. Many imputation methods have been proposed in the literature to handle such an issue. Complete case (CC), mean substitution (MS), last observation carried forward (LOCF), and multiple imputation (MI) are the four most frequently used methods in practice. In a real-world data analysis, the missing data can be MCAR, MAR, or MNAR, depending on why the data was missed. In this paper, simulations under various situations (including missing rates and slope sizes) were conducted to evaluate the performance of the four methods considered using bias, RMSE, and 95% coverage probability as evaluation measures. The results showed that LOCF has the most significant bias and the poorest 95% coverage probability in most cases under both MAR and MCAR assumptions. Hence, LOCF should not be used in a longitudinal data analysis. Under MCAR assumption, CC and MI methods performed equally well. Under MAR assumption, MI has the minor bias, smallest RMSE, and best 95% coverage probability. Therefore, the CC or MI method is the appropriate method to be used under MCAR. In contrast. the MI method is a more reliable and better-grounded statistical method under MAR MAR [10].

In 1996, rank set sampling (RSS) was studied by [5], which was suggested by [3] and developed by [4] to examine the ratio estimator. It was proved that RSS estimators were more efficient than simple random sampling (SRS). Later in 2003, the chain ratio (CR) estimator with SRS was examined [11]. The efficiency comparison used the mean squared error (MSE). They proved that the CR estimator was more efficient than the traditional ratio estimator under certain conditions.

A 2006 study investigated missing value estimation in multiple regression analysis. Three methods were used to estimate the missing values of a dependent variable: regression, EM algorithm, and pairwise deleted methods. The sample sizes were 20, 100, and 150. The missing values were 10%, 30%, and 50%. The error variances were 25, 50, 75, and 100. The evaluation measure was MSE. The study found that the regression method had the lowest MSE from all 500 iterations, outperforming other methods in many situations [12].

In 2009, modified ratio estimators of the population mean of a variable of interest were suggested, involving the first or third quartiles of an auxiliary variable correlated with the variable of interest. The newly suggested estimators were investigated using SRS and RSS methods. The efficiency was compared in terms of MSE. These estimators were unbiased, and the RSS estimators were more efficient than SRS estimators for the same quartile with the same correlation coefficient and sample size. Also, when the two quartiles were compared, an estimator based on the regression-ratio quartile3 (R-RQ3) imputation of Al-Omari, Jemain, and Ibrahim was more efficient than an estimator based on the regression-ratio quartile1 (R-RQ1) imputation of Al-Omari, Jemain, and Ibrahim [13].

In 2012, imputation methods were proposed when data on the dependent variable was missing in multiple linear regression analysis. The proposed methods of estimation were called ratio-Q1 imputation method (RQ1), ratio-Q3 imputation method (RQ3), regression-ratio-Q1 imputation method (R-RQ1), and regression-ratio-Q3 imputation method (R-RQ3). The efficiency of the proposed methods was compared to the mean and regression imputation method in a variance simulation situation. The evaluation measures were estimated root mean squares error (RMSE) and mean absolute percentage error (MAPE). In each tested situation, linear regression models with two independent variables were considered under the assumption that the error was distributed normally. The missing value is missing at random results showed that the R-RQ1 imputation of Al-Omari, Jemain, and Ibrahim was more efficient in the following situations: (1) a small sample size (n = 20), a large percentage of missing values (20%), a large value of variance (σ^2^ = 1.5, 2); (2) the medium sample size (n = 40, 60), a small percentage of missing values (10%), significant value of variance (σ^2^ = 0.5, 1); (3) the medium sample size (n = 40, 60), the medium percentage of missing values (15%), the medium value of variance (σ^2^ = 1, 1.5); and (4) a large sample size (n = 100), the medium percentage of missing values (15%), the medium value of variance (σ^2^ = 1.5). The R-RQ3 imputation of Al-Omari, Jemain, and Ibrahim was efficient for the situation of large sample size (n = 100), medium percentage of missing values (15%), and medium value of variance (σ^2^ = 1) [1].

In a later year, there was an investigation of the research [15] by [14] of a chain ratio (CR) estimator and a regression estimator with a linear combination of two auxiliary variables that used the supplementary data to increase the accuracy of the estimator. That study proposed a multivariate chain ratio (MCR) estimator and a regression estimator that used a linear combination of two auxiliary variables versus a traditional multivariate ratio (MR) estimator [15] and a regression estimator that used the information of two auxiliary variables [15], and a chain ratio (CR) estimator that used one auxiliary variable [11]. The evaluation measure was MSE. It was found that the proposed MCR estimator and the proposed regression estimator that used a linear combination of two auxiliary variables were equally highly effective, followed by the traditional MR estimator and the regression estimator that used the data of the two auxiliary variables [15].

Multiple Imputation, Maximum Likelihood, and Fully Bayesian methods are the three most used model-based approaches in missing data problems. Although it is easy to show that when the assumption is missing at random (MAR), the complete case analysis is unbiased and efficient, the methods above are still commonly used in practice for this setting. To examine the performance of relationships between these three methods in this setting, we derive and investigate small samples and asymptotic expressions of the estimates and standard errors. We thoroughly examine how these estimates are related to the three approaches in the linear regression model when the assumption is MAR. They show that when the assumption is MAR in the linear model, the estimates of the regression coefficients using these three methods are asymptotically equivalent to the complete case estimates under general conditions. A simulation of an accurate data set from a liver cancer clinical trial was conducted to compare the properties of these methods when the assumption was MAR [16].

In 2015, a paper proposed imputation estimators when there were missing observations on a dependent variable in multiple linear regression analysis. The proposed estimators were called ratio-Q1 (RQ1), ratio-Q3 (RQ3), regression-ratio-Q1 (R-RQ1) and regression-ratio-Q3 (R-RQ3). In various simulation situations, the efficiencies of the proposed estimations were compared to two existing methods—mean imputation and regression imputation. Mean absolute percentage error (MAPE) was the evaluation measure. For each situation, a multiple linear regression model with two independent variables was considered under the assumption that the error was distributed normally. Variances, sample sizes, and percentages of missing values were varied. Findings revealed that the MAPE from every estimator increased as either the percentage of missing values or the variance of error increased. Moreover, for all situations, R-RQ1, R-RQ3, and regression imputation performed better than the others [17].

In 2017, a study compared the ratio and regression estimators empirically based on bias and coefficient of variation. The author conducted simulation studies based on the sampling rate, population size, heterogeneity of the auxiliary variable X, deviation from linearity, and model misspecification. The study showed that the ratio estimator was better than the regression estimator when the regression line was close to the origin. Ratio and regression estimators worked even if there was a weak linear relationship between X and Y and if there was a minimum model misspecification. The relationship between the target and auxiliary variables was weak, Bootstrap estimated yield lower bias. The regression estimator was generally more efficient than the ratio estimator [18]. In regression analysis, missing covariate data has been a common problem. Many researchers use ad hoc methods to overcome this problem due to the ease of implementation. However, these methods require assumptions about the data that rarely hold in practice. Model-based methods such as Maximum Likelihood (ML) using the expectation maximization (EM) algorithm and Multiple Imputation (MI) are more promising when dealing with difficulties caused by missing data. Then again, inappropriate methods of missing value imputation can lead to serious bias that severely affects the parameter estimates. A simulation study was performed to assess the effects of different missing data techniques on the performance of a regression model. The covariate data were generated using an underlying multivariate normal distribution, and the dependent variable was generated using a combination of explanatory variables. Missing values in covariates were simulated using an assumption called missing at random (MAR). Four levels of missingness (10%, 20%, 30%, and 40%) were assessed. A linear regression analysis was fitted, and the model performance was measured; MSE, and R-squared were obtained. The study showed that MI was superior in handling missing data with the highest R-squared and the lowest MSE when the percentage of missingness was less than 30%. Both methods could not hold a larger than 30% level of missingness [19].

In 2021, Little reviewed assumptions about missing data mechanisms that underlie methods for the statistical analysis of data with missing values. Little explained Rubin’s original definition of missing at random (MAR), its motivation and criticisms, and his sufficient conditions for ignoring the missingness mechanism for likelihood-based, Bayesian, and frequentist inference. Related definitions, including missing completely at random (MCAR), missing at random (MAR), and partially MAR. Little presented a formal argument for weakening Rubin’s sufficient conditions for frequentist maximum likelihood and inference with precision based on the observed information. Some simple examples of MAR are described, with an example where the missingness mechanism can be ignored even though MAR does not hold. Alternative approaches to statistical inference based on the likelihood function were reviewed, along with non-likelihood frequentist approaches, including weighted generalized estimating equations. Connections with the causal inference literature were also discussed [20].

Missing data has been a common issue in many domains of study. If this issue was disregarded, an erroneous conclusion might ensue. In 2022, a study developed new imputation methods and compared the efficiency of eight imputation methods: hot deck imputation (HD), k-nearest neighbors imputation (KNN), stochastic regression, imputation (SR), predictive mean matching imputation (PMM), random forest imputation (RF), stochastic regression random forest with equivalent weight imputation (SREW), k-nearest random forest with equivalent weight imputation (KREW), and k-nearest stochastic regression and random forest with equivalent weight imputation (KSREW). Simulations were run using various sample sizes (30, 60, 100, and 150) and missing percentages (10%, 20%, 30%, and 40%). Average mean square error (AMSE) was used to compare the efficiencies. The proposed composite approach outperformed the single ones. On the other hand, increasing the number of components to a four-component method did not affect the imputation performance [21].

In 2022, Isabella Sayers et al. conducted a comparison of five missing value imputation methods in multiple regression model: multiple regression, regression-ratio-Q1 (R-RQ1), regression-ratio-Q3 (R-RQ3), stochastic regression, and k-nearest stochastic regression, with equivalent weighting methods under missing completely at random (MCAR). The sample size, error variance, and missing values percentage varied. The evaluation measure was MSE. The small sample sizes were 20 and 40; the medium sample sizes were 60 and 80, and the large sample sizes were 100 and 120. The percentages of missing values were 5, 10, 15, and 20. The missing values were constructed by a missing-at-random method. The variances of error used in this study were 1, 3, 5, 7, and 9. The research result showed that, for all percentages of missing values, all variances of error, and all sample sizes, most of the R-RQ1 method is the maximum efficiency, followed by the multiple regression method [22].

In 2024, Song and Guo presented a fully informative multiple imputation (fiMI) method. It was based on a linear regression model with a missing response variable, utilizing all observable data to obtain estimates of the regression coefficients and, thereby, the predicted values of the missing response variable. This provided a good explanation of the relationship between the response variable and their respective variables and effectively enhanced the imputation accuracy of the response variable. The stability and sensitivity of the fiMI method were evaluated through a simulation study. Subsequently, the proposed fiMI method was applied to two real data sets: the admission prediction data set and the goalkeeper data set [23].

The studies above inspired the authors to study the use of a ratio estimator that utilizes the 1st and 3rd quartiles [13]. The estimator was used to estimate the missing values in multiple regression analysis with a simple random sampling (SRS). An efficient missing-value imputation method was the regression-ratio quartile1,3 imputation with SRS of Al-Omari, Jemain, and Ibrahim. This method was highly efficient [1, 22]. The authors propose three new adjusted methods for comparison: the adjusted regression-chain ratio quartile1,3 imputation with SRS of Kadilar and Cingi from the research [11], the adjusted regression-multivariate ratio quartile1,3 imputation with SRS of Feng, Ni, and Zou from the research [14], which provided highly efficient, and the adjusted regression-multivariate chain ratio quartile1,3 imputation with SRS of Lu from the research [15], which provided the highest efficiency.

In addition, the research compares an estimator of the utilization ratio of the 1st and 3rd quartiles analyzed with a rank set sampling (RSS) (referenced in [18]) versus simple random sampling. It was found that the efficiencies of the utilization ratio estimators of the 1st and 3rd quartiles analyzed with RSS were higher than those analyzed with SRS. Interested in developing further, the authors propose four new adjusted methods for estimating missing values in regression analysis: the adjusted regression-ratio quartile1,3 imputation with RSS of Al-Omari, Gemain, and Ibrahim from the proposed ratio estimator [13]; the adjusted regression-chain ratio quartile1,3 imputation with RSS of Kadilar and Cinji from the proposed chain ratio estimator [11]; the adjusted regression-multivariate ratio quartile1,3 imputation with RSS of Feng, Ni, and Zou from the proposed multivariate ratio estimator [14]; and the adjusted regression-multivariate chain ratio quartile1,3 imputation with RSS of Lu from the proposed multivariate chain ratio estimator [15]. The efficiency measures were mean square error (MSE) and mean absolute percentage error (MAPE). The optimum method was used to select a proper missing value imputation method under different sample sizes, error variances, and percentages of missing values. Moreover, the research also compared the performance of SRS and RSS estimators considering the MSE and MAPE.

Our contributions to the field of statistics are tables suggesting the best missing value imputation method for multiple regression analysis based on sampling method, sample size, missing value percentage, and error variance. In addition, the table suggested a better sampling method for SRS and RSS estimators based on sample sizes and error variances.

## 2. Materials and methods

This research compared new adjusted missing-value imputation methods in multiple regression with simple random sampling and rank-set sampling methods. The measures for comparing missing value imputation were mean square error and mean absolute percentage error. The study comprised many steps, as follows.

2.1 The population data of the independent variables (*X*_1_,*X*_2_) and the error (*✓*) were constructed with a size of 100,000 values. This data had a normal distribution with the probability density function,


$$\begin{array}{ccc} f(x)=\frac{1}{\sigma \sqrt{2\pi }}e^{-\frac{\frac{1}{}}{2}{\left(\frac{x-\mu }{\sigma }\right)}^{2}};-\infty <x<\infty ,-\infty <\mu <\infty ,\sigma^{2}>0. & & \end{array}$$ (1)

where expected value was *E* (*X*)=*μ*, and variance was *Var* (*X*)=*σ*^2^. The independent variable *X*_1_ had parameters *μ*=3,*σ*^2^=2.25. The independent variable *X*_2_ had parameters *μ* = 5, *σ*^2^=4, and the error had parameters *μ*=0,*σ*^2^=1,3,5,7,9 [1].

2.2 The above independent variables and the error were sampled with simple random sampling (SRS) and rank set sampling (RSS) methods for a small sample size of 20 and 40; a middle sample size of 60 and 80; a large sample size of 100 and 120; a considerable sample size of 200 and 500. For each sampling instance, it was repeated 1,000 times [1].

2.3 The multicollinearity between independent variables was tested whether the independent variables were correlated or not by using the Pearson correlation coefficient. If the correlation coefficient was more significant than or equal to 0.6, return to sampling in Sect 2.2 again. The multicollinearity was tested, and the variables were resampled repeatedly until the correlation coefficient was less than 0.6, indicating that the independent variables were not correlated [24, 25]. The construction of the dependent variable (*Y *) is described in Sect 2.5. Multicollinearity can be tested based on VIF and Pearson correlation coefficient. In this research, we used the Pearson correlation coefficient because it was easier to use the values of the independent variables to determine the correlation coefficient without needing to determine the value of the dependent variable’s value first.

2.4 The parameter of the y-intercept was set to *β*_0_=0.5, and the regression coefficients were set to *β*_1_=1 and *β*_2_=−0.3 to create the dependent variable in Sect 2.5.

2.5 The dependent variable *Y * was linearly correlated with the independent variables *X*_1_ and *X*_2_. The y-intercept *β*_0_, regression coefficient *β*_1_ and *β*_1_, and error *μ* = 5 were constructed using the following linear relationship model,


$$\begin{array}{ccc} Y_{i}=\beta_{0}+\beta_{1}X_{1i}+\beta_{2}X_{2i}+\epsilon_{i};i=1,2,...,n, & & \end{array}$$ (2)

where *Y_i_* was the dependent variable; *X*_1*i*_ and *X*_2*i*_ were the 1st and 2nd independent variables; *β*_0_ was the y-intercept or value of *Y * for *X*_1*i*_=0 and *X*_2*i*_=0, *β*_1_ and *β*_2_ were the regression coefficient or slope of the straight line; and *ϵ_i_* was the error with *μ* = 0, and *σ*^2^=1,2,3,4 and 5 [1].

2.6 The independent variable *X*_2_ had missing values of 5, 10, 15, 20, 30, and 40 percentages missing completely at random (MCAR) [6, 9].

2.7 The number of missing values was calculated, and the positions of missing values were randomized.

2.7.1 The number of missing values of the independent variable *X*_2_ was calculated as follows:

Number of missing values = Sample sizes x Missing value percentages

2.7.2 The positions of missing values of the independent variable *X*_2_ were randomized and determined as missing completely at random (MCAR). MCAR was assumed in this simulation study. The missing values in *X*_2_ were not related to *X*_1_. In the simulation, they were simulated independently of each other. The positions of the missing values in *X*_2_ were randomized, they were replaced with the series mean of the entire data set in *X*_2_. Missing values of 5, 10, 15, 20, 30, and 40% are shown in Table 1.

**Table 1:** Number of missing values and number of intact data for missing value percentages of 5, 10, 15, 20, 30, and 40 in small, middle, large, and huge sample sizes.

	Missing value percentages											
	5		10		15		20		30		40	
Sample sizes (n)	No. of missing values	No. of intact data	No. of missing values	No. of intact data	No. of missing values	No. of intact data	No. of missing values	No. of intact data	No. of missing values	No. of intact data	No. of missing values	No. of intact data
Small													
20	1	19	2	18	3	17	4	16	6	14	8	12
40	2	38	4	36	6	34	8	32	12	28	16	24
Middle													
60	3	57	6	54	9	51	12	48	18	42	24	36
80	4	76	8	72	12	68	16	64	24	56	32	48
Large													
100	5	95	10	90	15	85	20	80	30	70	40	60
120	6	114	12	108	18	102	24	96	36	84	48	72
Huge													
200	10	190	20	180	30	170	40	160	60	140	80	120
500	25	475	50	450	75	425	100	400	150	350	200	300

Sample sizes provided eight values, the proportions of missing values provided six values, and missing value estimation methods provided eight values, which had quite a broad scope in multiple regression analysis with two covariates and MCAR. There were 8*x*6*x*8 = 384 scenarios for each error variance of the sampling method.

2.8 The missing values of the independent variable *X*_2_ were calculated using the series mean, which replaced the missing values with the mean of the entire data set in *X*_2_ as follows [26]:


$$\begin{array}{ccc} \hat{X}=\frac{\overset{k}{\underset{i}{\sum }}}{k}X_{i}, & & \end{array}$$ (3)

where *X_i_* was the data value with available values; *k* was the amount of data with available values; and $\hat{X}$ was the estimate of the missing value predicted from the mean of the available values.

2.9 In Sect 2.8, the estimates of the missing values of the independent variable *X*_2_ were placed in the positions of the missing values.

2.10 The regression-ratio imputations were replaced in all eight methods as follows:

2.10.1 Regression-ratio quartile1,3 imputation with SRS of Al-Omari, Jemain, and Ibrahim (R-RQ1,3SRS)

Regression-ratio quartile1 imputation (R-RQ1) was a ratio imputation estimation method applied by replacing ${ŷ}_{reg}$ to *ȳ*. This method uses advantage of the 1st quartile of variables *X*_1_ and *X*_2_ [1]. Regression-ratio quartile3 imputation (R-RQ3) is described in the same way.

2.10.1.1 Multiple regression imputation mechanisms of MCAR

Regression-ratio quartile1,3 imputation with SRS of Al-Omari, Jemain, and Ibrahim relies on the imputation of the multiple regression ${ŷ}_{reg}$ into *ȳ* for the missing value estimator presented in Sect 2.10.1.4, as follows:

1) The data *X*_2,*r*+1,...,_*X*_2*n*_, the missing values of the independent variables, were replaced with *X*_2,*r*+1,*new*,...,_*X*_2*n*,*new*_. The estimate of the dependent variable ${Ŷ}_{i}$ was calculated using the missing value estimation with multiple regression analysis. 2) The independent variable *X*_1*i*_ with complete information and the independent variable *X*_2*i*,*new*_ with replaced missing values provided the estimation of the dependent variable ${Ŷ}_{i}$ as follows: ${Ŷ}_{i}={\hat{\beta }}_{0}+{\hat{\beta }}_{1}X_{1i}+{\hat{\beta }}_{2}X_{2i}$.

2.10.1.2 Ratio estimator with simple random sampling

The estimators, and (11), that we used in this study, were derived ultimately from the ratio estimator with simple random sampling (R-SRS). Here, we show the derivation steps from the equation of R-SRS to and (11) in the subsections below. Simple random sampling is a sampling of a population of size N, where each possible sample of size n had the same probability of being selected. The sample obtained at such sampling is called a simple random sample [2].

The ratio estimator (${\hat{\mu }}_{Y}$) is a method for estimating *μ_Y_* with ${\hat{\mu }}_{Y}$ based on the relationship between the auxiliary variable *X* (e.g. sample mean of $\bar{x}$) and the dependent variable *Y * (e.g. sample mean of *ȳ*) under the condition that the population mean $\mu_{X}$ is a known parameter. We defined it under simple random sampling as follows [13]:


$$\begin{array}{ccc} {\hat{\mu }}_{YSRS}=\mu_{X}\left(\frac{{ȳ}_{SRS}}{{\bar{x}}_{SRS}}\right), & & \end{array}$$ (4)

where ${\hat{\mu }}_{YSRS}$ was a ratio estimate, ${\bar{x}}_{SRS}=\frac{\sum_{i}^{n}}{n}x_{i}$ and ${ȳ}_{SRS}=\frac{\sum_{i}^{n}}{n}y_{i}$ were the sample mean of the independent variable *X*_2_ and the dependent variable *Y *, respectively.

2.10.1.3 Ratio estimator using quartile1,3 (RQ1,3)

AI-Omari et al. (2009) proposed the following ratio estimator [13],


$$\begin{array}{ccc} {\hat{\mu }}_{YR}={ȳ}_{SRS}\left(\frac{\mu_{X}}{{\bar{x}}_{SRS}}\right). & & \end{array}$$ (5)

In addition, they also proposed a ratio estimator for estimating the population mean of the dependent variable by taking advantage of the 1st and 3rd quartiles of the independent variable *X*_1_ and *X*_2_. The calculation formula is the following,


$$\begin{array}{ccc} {\hat{\mu }}_{YRQ1}={ȳ}_{SRS}\left(\frac{\mu_{X}+q_{1}}{{\bar{x}}_{SRS}+q_{1}}\right), & & \end{array}$$ (6)

and


$$\begin{array}{ccc} {\hat{\mu }}_{YRQ3}={ȳ}_{SRS}\left(\frac{\mu_{X}+q_{3}}{{\bar{x}}_{SRS}+q_{3}}\right), & & \end{array}$$ (7)

where ${\hat{\mu }}_{YRQ1}$ was an estimate of the mean using quartile1, ${\hat{\mu }}_{YRQ3}$ was an estimate of the mean using quartile3; ${\bar{x}}_{SRS}$ and ${ȳ}_{SRS}$ were the sample mean of the independent variable *X*_2_ and the dependent variable *Y *, respectively; *q*_1_ and *q*_3_ were quartile1 and 3 of the population of the independent variable *X*_2_, respectively.

2.10.1.4 Missing value estimator

In and (7), Jomprapan (2012) [1] substitute ${\bar{x}}_{in}$ for *μ_X_* since it is difficult to obtain the population mean. Therefore, the sample mean ${\bar{x}}_{in}$ is instead of the population mean *μ_X_* and the sample mean ${\bar{x}}_{ir}$ was instead of the sample mean ${\bar{x}}_{SRS}$. Two estimators of missing value were obtained as and ((9), which are called ratio quartile1 imputation with simple random sampling (RQ1SRS) and ratio quartile3 imputation with simple random sampling (RQ3SRS),


$$\begin{array}{ccc} {ŷ}_{RQ1SRS}=ȳ\overset{k}{\underset{i=1}{\prod }}\left(\frac{{\bar{x}}_{in}+q_{1}}{{\bar{x}}_{ir}+q1}\right), & & \end{array}$$ (8)

and


$$\begin{array}{ccc} {ŷ}_{RQ3SRS}=ȳ\overset{k}{\underset{i=1}{\prod }}\left(\frac{{\bar{x}}_{in}+q_{3}}{{\bar{x}}_{ir}+q3}\right), & & \end{array}$$ (9)

where ${ŷ}_{RQ1SRS}$ was a ratio quartile1 estimate, ${ŷ}_{RQ3SRS}$ was a ratio quartile3 estimate, ${\bar{x}}_{in}$ was a sample mean of the independent variable *X*_2_ when the data was complete, ${\bar{x}}_{ir}$ was a sample mean of the independent variable *X*_2_ without accounting for the missing values, *ȳ* was a mean of the complete dependent variable *Y * and *q*_1_, *q*_3_ were quartile1, 3 of the samples of the random variable *X*_2_, respectively [1, 17].

However, using only one estimator (*ȳ*) for all missing values may not be appropriate because using a constant for all missing values causes the estimator’s variance to be underestimated. Therefore, she took the estimate from the multiple regression analysis, ${ŷ}_{reg}$ substituted it in *ȳ* in and (9), resulting in and (11), which are called regression-ratio quartile1 imputation with a simple random sampling of Al-Omari, Jemain, and Ibrahim (R-RQ1SRS) and regression-ratio quartile3 imputation with a simple random sampling of Al-Omari, Jemain, and Ibrahim (R-RQ3SRS),


$$\begin{array}{ccc} {ŷ}_{R-RQ1SRS}={ŷ}_{reg}\overset{k}{\underset{i=1}{\prod }}\left(\frac{{\bar{x}}_{in}+q_{1}}{{\bar{x}}_{ir}+q1}\right), & & \end{array}$$ (10)

and


$$\begin{array}{ccc} {ŷ}_{R-RQ3SRS}={ŷ}_{reg}\overset{k}{\underset{i=1}{\prod }}\left(\frac{{\bar{x}}_{in}+q_{3}}{{\bar{x}}_{ir}+q3}\right), & & \end{array}$$ (11)

where ${ŷ}_{R-RQ1SRS}$ was the regression-ratio quartile1 imputation estimate with a simple random sampling; ${ŷ}_{R-RQ3SRS}$ was the regression-ratio quartile3 imputation estimate with a simple random sampling; and ${ŷ}_{reg}$ was the value of the dependent variable *Y * for multiple regression analysis.

The ${ŷ}_{R-RQ1SRS}$ and ${ŷ}_{R-RQ3SRS}$ were calculated from and (11), respectively, which were substituted in the same position as the missing value of the independent variable *X*_2_ for the estimable dependent variable ${ŷ}_{i}$ in Sect 2.10.1.1.

The authors propose three adaptation methods, based on ideas from [11, 14, 15], taking advantage of the 1st and 3rd quartiles and ${ŷ}_{reg}$ was obtained by imputation with multiple regression analysis to replace *ȳ* using simple random sampling as follows in Sect 2.10.2–2.10.4.

2.10.2 Adjusted regression-chain ratio quartile1,3 imputation with SRS of Kadilar and Cingi (AR-CRQ1,3SRS)

Kadilar and Cingi (2003) proposed the following chain ratio estimator [11],


$$\begin{array}{ccc} {\hat{\mu }}_{YCR}={ȳ}_{SRS}{\left(\frac{\mu_{X}}{{\bar{x}}_{SRS}}\right)}^{\alpha }. & & \end{array}$$ (12)

In addition to the two methods proposed in Sect 2.10.1.4 by [1], the authors propose another technique, a new adaptation of the chain ratio method [11], for estimating missing values, that took advantage of the 1st and 3rd quartiles of variables *X*_2_. The technique is the following Equations,


$$\begin{array}{ccc} {\hat{\mu }}_{YCRQ1}={ȳ}_{SRS}{\left(\frac{\mu_{X}+q_{1}}{{\bar{x}}_{SRS}+q_{1}}\right)}^{\alpha }, & & \end{array}$$ (13)

and


$$\begin{array}{ccc} {\hat{\mu }}_{YCRQ3}={ȳ}_{SRS}{\left(\frac{\mu_{X}+q_{3}}{{\bar{x}}_{SRS}+q_{3}}\right)}^{\alpha }, & & \end{array}$$ (14)

The authors also propose using ${ŷ}_{reg}$ to replace *ȳ* in and (14). Hence, *μ_X_* was substituted by ${\bar{x}}_{in}$ since obtaining the population mean was difficult. Also, ${\bar{x}}_{ir}$ was used instead of ${\bar{x}}_{SRS}$. Two estimators of missing values were obtained as and (16). These are called the adjusted regression-chain ratio quartile1 imputation with a simple random sampling of Kadilar and Cingi (AR-CRQ1SRS) and the adjusted regression-chain ratio quartile3 imputation with a simple random sampling of Kadilar and Cingi (AR-CRQ3SRS),


$$\begin{array}{ccc} {ŷ}_{AR-CRQ1SRS}={ŷ}_{reg}\overset{k}{\underset{i=1}{\prod }}{\left(\frac{{\bar{x}}_{in}+q_{1}}{{\bar{x}}_{ir}+q_{1}}\right)}^{\alpha }, & & \end{array}$$ (15)

and


$$\begin{array}{ccc} {ŷ}_{AR-CRQ3SRS}={ŷ}_{reg}\overset{k}{\underset{i=1}{\prod }}{\left(\frac{{\bar{x}}_{in}+q_{3}}{{\bar{x}}_{ir}+q_{3}}\right)}^{\alpha }, & & \end{array}$$ (16)

where ${ŷ}_{AR-CRQ1SRS}$ was the adjusted regression-chain ratio quartile1 imputation estimate with a simple random sampling; ${ŷ}_{AR-CRQ3SRS}$ was the adjusted regression-chain ratio quartile3 imputation estimate with a simple random sampling; and *α* was any constant value. In this case, we set *α* = 0 . 10 , 0 . 30 , 0 . 50 , 0 . 70 , 0 . 90. The experiment showed that *α* = 0 . 90 provided the best estimation performance.

2.10.3 Adjusted regression-multivariate ratio quartile1,3 imputation with SRS of Feng, Ni, and Zou (AR-MRQ1,3SRS)

Feng et al. (1998) proposed a multivariate ratio estimator [14],


$$\begin{array}{ccc} {\hat{\mu }}_{YMR}=\epsilon {ȳ}_{SRS}\left(\frac{\mu_{X}}{{\bar{x}}_{SRS}}\right). & & \end{array}$$ (17)

The authors propose a new adaptation of the multivariate ratio method of [14]. The adapted methods estimated missing values by taking advantage of the 1st and 3rd quartiles of variables *X*_1_ and *X*_2_,


$$\begin{array}{ccc} {\hat{\mu }}_{YMRQ1}=\epsilon {ȳ}_{SRS}\left(\frac{\mu_{X}+q_{1}}{{\bar{x}}_{SRS}+q_{1}}\right), & & \end{array}$$ (18)

and


$$\begin{array}{ccc} {\hat{\mu }}_{YMRQ3}=\epsilon {ȳ}_{SRS}\left(\frac{\mu_{X}+q_{3}}{{\bar{x}}_{SRS}+q_{3}}\right). & & \end{array}$$ (19)

In addition, the authors also propose using ${ŷ}_{reg}$ to replace *ȳ* in and (19) and replacing *μ_X_* with ${\bar{x}}_{in}$. Also, ${\bar{x}}_{ir}$ replaced ${\bar{x}}_{SRS}$, resulting in two missing value estimators defined by and (21). These are called the adjusted regression-multivariate ratio quartile1 imputation with a simple random sampling of Feng, Ni, and Zou (AR-MRQ1SRS) and the adjusted regression-multivariate ratio quartile3 imputation with a simple random sampling of Feng, Ni, and Zou (AR-MRQ3SRS),


$$\begin{array}{ccc} {ŷ}_{AR-MRQ1SRS}={ŷ}_{reg}[\epsilon_{1}\left(\frac{{\bar{x}}_{1n}+q_{1}}{{\bar{x}}_{1r}+q_{1}}\right)+\epsilon_{2}\left(\frac{{\bar{x}}_{2n}+q_{1}}{{\bar{x}}_{2r}+q_{1}}\right)], & & \end{array}$$ (20)

and


$$\begin{array}{ccc} {ŷ}_{AR-MRQ3SRS}={ŷ}_{reg}[\epsilon_{1}\left(\frac{{\bar{x}}_{1n}+q_{3}}{{\bar{x}}_{1r}+q_{3}}\right)+\epsilon_{2}\left(\frac{{\bar{x}}_{2n}+q_{3}}{{\bar{x}}_{2r}+q_{3}}\right)], & & \end{array}$$ (21)

where ${ŷ}_{AR-MRQ1SRS}$ was the adjusted regression-multivariate ratio quartile1 imputation estimate with a simple random sampling; ${ŷ}_{AR-MRQ3SRS}$ was the adjusted regression-multivariate ratio quartile3 imputation estimate with a simple random sampling; and *ϵ*_1_,*ϵ*_2_ were the weighted values, and *ϵ*_1_+*ϵ*_2_=1. In this case, we set *ϵ*_1_=0.10,0.30,0.50,0.70,0.90 and *ϵ*_2_=0.10,0.30,0.50,0.70,0.90. By trial and error, *ϵ*_1_=0.9 and *ϵ*_2_=0.10 were found to give the best estimation performance.

2.10.4 Adjusted regression-multivariate chain ratio quartile1,3 imputation with SRS of Lu (AR-MCRQ1,3SRS)

Lu (2013) proposed a multivariate chain ratio estimator [15],


$$\begin{array}{ccc} {\hat{\mu }}_{YMCR}={ȳ}_{SRS}{\left(\frac{\omega_{1}{\bar{x}}_{1n}+\omega_{2}{\bar{x}}_{2n}}{\omega_{1}{\bar{x}}_{1r}+\omega_{2}{\bar{x}}_{2r}}\right)}^{\alpha }. & & \end{array}$$ (22)

The authors propose a new adaptation of the multivariate chain ratio method [15]. This adaptation estimated the missing values by taking advantage of the 1st and 3rd quartiles of variables *X*_1_ and *X*_2_,


$$\begin{array}{ccc} {\hat{\mu }}_{YMCRQ1}={ȳ}_{SRS}{\left(\frac{\omega_{1}\left({\bar{x}}_{1n}+q_{1}\right)+\omega_{2}({\bar{x}}_{2n}+q_{1})}{\omega_{1}({\bar{x}}_{1r}+q_{1})+\omega_{2}({\bar{x}}_{2r}+q_{1})}\right)}^{\alpha }, & & \end{array}$$ (23)

and


$$\begin{array}{ccc} {\hat{\mu }}_{YMCRQ3}={ȳ}_{SRS}{\left(\frac{\omega_{1}\left({\bar{x}}_{1n}+q_{3}\right)+\omega_{2}({\bar{x}}_{2n}+q_{3})}{\omega_{1}({\bar{x}}_{1r}+q_{3})+\omega_{2}({\bar{x}}_{2r}+q_{3})}\right)}^{\alpha }. & & \end{array}$$ (24)

In addition, the authors also propose using ${ŷ}_{reg}$ to replace *ȳ* in and (24), resulting in two missing value estimators defined by and (26). These are called the adjusted regression-multivariate chain ratio quartile1 imputation with a simple random sampling of Lu (AR-MCRQ1SRS) and the adjusted regression-multivariate chain ratio quartile3 imputation with a simple random sampling of Lu (AR-MCRQ3SRS),


$$\begin{array}{ccc} {ŷ}_{AR-MCRQ1SRS}={ŷ}_{reg}{\left(\frac{\omega_{1}\left({\bar{x}}_{1n}+q_{1}\right)+\omega_{2}({\bar{x}}_{2n}+q_{1})}{\omega_{1}({\bar{x}}_{1r}+q_{1})+\omega_{2}({\bar{x}}_{2r}+q_{1})}\right)}^{\alpha }, & & \end{array}$$ (25)

and


$$\begin{array}{ccc} {ŷ}_{AR-MCRQ3SRS}={ŷ}_{reg}{\left(\frac{\omega_{1}\left({\bar{x}}_{1n}+q_{3}\right)+\omega_{2}({\bar{x}}_{2n}+q_{3})}{\omega_{1}({\bar{x}}_{1r}+q_{3})+\omega_{2}({\bar{x}}_{2r}+q_{3})}\right)}^{\alpha }, & & \end{array}$$ (26)

where ${ŷ}_{AR-MCRQ1SRS}$ was the adjusted regression-multivariate chain ratio quartile1 imputation estimate with a simple random sampling; ${ŷ}_{AR-MCRQ3SRS}$ was the adjusted regression-multivariate chain ratio quartile3 imputation estimate with a simple random sampling; *α* was any constant value; and *ω*_1_,*ω*_2_ were the weighted values, and *ω*_1_+*ω*_2_=1. We set *α*=0.10,0.30,0.50,0.70,0.90; *ω*_1_=0.10,0.30,0.50,0.70,0.90, and *ω*_2_=0.10,0.30,0.50,0.70,0.90. The experiment showed that *α* = 0 . 10, *ω*_1_=0.10, and *ω*_2_=0.90 gave the best estimation performance.

According to the research of [13], a ratio estimator was proposed by taking advantage of the 1st and 3rd quartiles with a rank set sampling (RSS) (referenced in [18]) compared with a simple random sampling (SRS). It was found that the ratio estimator of the 1st and 3rd quartiles with RSS had higher efficiency than SRS. Therefore, the authors adopted this concept and proposed four additional adjustment methods for estimating missing value in the regression analysis: [11, 13–15] by utilization of the 1st and 3rd quartiles and using ${ŷ}_{reg}$ which was *ŷ* obtained by imputation of the regression method to replace *ȳ* by RSS as follows:

2.10.5 Adjusted regression-ratio quartile1,3 imputation with RSS of Al-Omari, Jemain, and Ibrahim (AR-RQ1,3RSS)

2.10.5.1 Multiple regression imputation

This imputation was similar to Sect 2.10.1.1, regression-ratio quartile1,3 imputation with RSS of Al-Omari, Jemain, and Ibrahim relies on the imputation the multiple regression, ${ŷ}_{reg}$ into *ȳ* in the missing value estimator presented in Sect 2.10.5.3.

2.10.5.2 Ratio estimator with a rank set sampling (R-RSS)

A rank set sampling method followed the steps below [13, 18].

1) The first data set of size n units was randomized. Then, the smallest value of the independent variable *X*, which was correlated to the value of the dependent variable *Y *, was selected.

2) The second data set of size n units was randomized. Then, the second smallest value of the independent variable, which was correlated to the value of the dependent variable, was selected, and so on.

3) The nth data set of size n units was randomized. Then, the nth most significant value of the independent variable, which was correlated to the value of the dependent variable, was selected.

The selected data sets were used to estimate the missing values. A rank set sampling estimator *μ_Y_* was based on the relationship between the auxiliary variable *X* and the dependent variable *Y *, assuming that the population mean *μ_X_* was a known parameter. We could define it under rank set sampling as follows [13],


$$\begin{array}{ccc} {\hat{\mu }}_{YRSS}=\mu_{X}\left(\frac{{ȳ}_{RSS}}{{\bar{x}}_{RSS}}\right), & & \end{array}$$ (27)

where ${\hat{\mu }}_{YRSS}$ was a ratio estimate; ${\bar{x}}_{RSS}=\frac{\sum_{i}^{n}}{n}x_{i}$ and ${ȳ}_{RSS}=\frac{\sum_{i}^{n}}{n}y_{i}$ were the sample mean of the independent variable *X*_2_ and the dependent variable *Y *, respectively.

2.10.5.3 Proposed missing value estimator

AI-Omari et al. (2009) proposed the following ratio estimator [13],


$$\begin{array}{ccc} {\hat{\mu }}_{YR}={ȳ}_{RSS}\left(\frac{\mu_{X}}{{\bar{x}}_{RSS}}\right). & & \end{array}$$ (28)

In addition, they also proposed a ratio estimator to estimate the population mean of the dependent variable by taking advantage of the 1st and 3rd quartiles of the variables *X*_1_ and *X*_2_. The calculation formula is the following,


$$\begin{array}{ccc} {\hat{\mu }}_{YRQ1}={ȳ}_{RSS}\left(\frac{\mu_{X}+q_{1}}{{\bar{x}}_{RSS}+q_{1}}\right), & & \end{array}$$ (29)

and


$$\begin{array}{ccc} {\hat{\mu }}_{YRQ3}={ȳ}_{RSS}\left(\frac{\mu_{X}+q_{3}}{{\bar{x}}_{RSS}+q_{3}}\right), & & \end{array}$$ (30)

where ${\hat{\mu }}_{YRQ1}$ was an estimate of the mean using quartile1, ${\hat{\mu }}_{YRQ3}$ was an estimate of the mean using quartile3; ${\bar{x}}_{RSS}$ and ${ȳ}_{RSS}$ were sample mean of the independent variable *X*_2_ and the dependent variable *Y *, respectively; *q*_1_ and *q*_3_ were quartile1 and 3 of the population of the independent variable *X*_2_, respectively.

The authors propose a new adjusted ratio method of [13] using ${ŷ}_{reg}$, which was *ŷ* obtained by imputation with the multiple regression method, to replace *ȳ* in and (30). The population mean (*μ_X_*) was substituted by the sample mean (${\bar{x}}_{in}$) since obtaining the population mean was difficult. The sample mean (${\bar{x}}_{ir}$) was substituted into the sample mean (${\bar{x}}_{RSS}$), resulting in two missing value estimators defined in and (32) below. We called these estimators an adjusted regression-ratio quartile1 imputation with a rank set sampling of Al-Omari, Jemain, and Ibrahim (AR-RQ1RSS) and an adjusted regression-ratio quartile3 imputation with a rank set sampling of Al-Omari, Jemain, and Ibrahim (AR-RQ3RSS),


$$\begin{array}{ccc} {ŷ}_{AR-RQ1RSS}={ŷ}_{reg}\overset{k}{\underset{i=1}{\prod }}\left(\frac{{\bar{x}}_{in}+q_{1}}{{\bar{x}}_{ir}+q_{1}}\right), & & \end{array}$$ (31)

and


$$\begin{array}{ccc} {ŷ}_{AR-RQ3RSS}={ŷ}_{reg}\overset{k}{\underset{i=1}{\prod }}\left(\frac{{\bar{x}}_{in}+q_{3}}{{\bar{x}}_{ir}+q_{3}}\right), & & \end{array}$$ (32)

where ${ŷ}_{AR-RQ1RSS}$ was the adjusted regression-ratio quartile1 imputation estimate with a rank set random sampling; ${ŷ}_{AR-RQ3RSS}$ was the adjusted regression-ratio quartile3 imputation estimate with a rank set sampling; and ${ŷ}_{reg}$ was the value of the dependent variable *Y *.

2.10.6 Adjusted regression-chain ratio quartile1,3 imputation with RSS of Kadilar and Cingi (AR-CRQ1,3RSS)

Kadilar and Cingi (2003) proposed the following chain ratio estimator [11],


$$\begin{array}{ccc} {\hat{\mu }}_{YCR}={ȳ}_{RSS}{\left(\frac{\mu_{X}}{{\bar{x}}_{RSS}}\right)}^{\alpha }. & & \end{array}$$ (33)

The authors propose a new adaptation of the chain ratio method of [11], for estimating values that take advantage of the 1st and 3rd quartiles of variables *X*_1_ and *X*_2_. The technique is the following Equatios,


$$\begin{array}{ccc} {\hat{\mu }}_{YCRQ1}={ȳ}_{RSS}{\left(\frac{\mu_{X}+q_{1}}{{\bar{x}}_{RSS}+q_{1}}\right)}^{\alpha }, & & \end{array}$$ (34)

and


$$\begin{array}{ccc} {\hat{\mu }}_{YCRQ3}={ȳ}_{RSS}{\left(\frac{\mu_{X}+q_{3}}{{\bar{x}}_{RSS}+q_{3}}\right)}^{\alpha }. & & \end{array}$$ (35)

In addition, the authors also propose using ${ŷ}_{reg}$ to replace *ȳ* in and (35). Hence, ${\bar{x}}_{in}$ substituted *μ_X_* since it was difficult to obtain the population mean. Also, ${\bar{x}}_{ir}$ was used instead of ${\bar{x}}_{RSS}$. Two estimators of missing values were obtained as and (37). These are called the adjusted regression-chain ratio quartile1 imputation with a rank set sampling of Kadilar and Cingi (AR-CRQ1RSS) and the adjusted regression-chain ratio quartile3 imputation with a simple random sampling of Kadilar and Cingi (AR-CRQ3RSS),


$$\begin{array}{ccc} {ŷ}_{AR-CRQ1RSS}={ŷ}_{reg}\overset{k}{\underset{i=1}{\prod }}{\left(\frac{{\bar{x}}_{in}+q_{1}}{{\bar{x}}_{ir}+q_{1}}\right)}^{\alpha }, & & \end{array}$$ (36)

and


$$\begin{array}{ccc} {ŷ}_{AR-CRQ1RSS}={ŷ}_{reg}\overset{k}{\underset{i=1}{\prod }}{\left(\frac{{\bar{x}}_{in}+q_{3}}{{\bar{x}}_{ir}+q_{3}}\right)}^{\alpha }, & & \end{array}$$ (37)

where ${ŷ}_{AR-CRQ1RRS}$ was the adjusted regression-chain ratio quartile1 imputation estimate with a rank set sampling; ${ŷ}_{AR-CRQ3RSS}$ was the adjusted regression-chain ratio quartile3 imputation estimate with a rank set sampling; and *α* was any constant value. In this case, we set *α* = 0 . 10 , 0 . 30 , 0 . 50 , 0 . 70 , 0 . 90. The experiment showed that *α* = 0 . 90 provided the best estimation performance.

2.10.7 Adjusted regression-multivariate ratio quartile1,3 imputation with RSS of Feng, Ni, and Zou (AR-MRQ1,3RSS)

Feng et al. (1998) proposed a multivariate ratio estimator [14],


$$\begin{array}{ccc} {\hat{\mu }}_{YMR}=\epsilon {ȳ}_{RSS}\left(\frac{\mu_{X}}{{\bar{x}}_{RSS}}\right). & & \end{array}$$ (38)

The authors propose a new adaptation of the multivariate ratio method [14]. The adapted methods estimated missing values by taking advantage of the 1st and 3rd quartiles of variables *X*_1_ and *X*_2_,


$$\begin{array}{ccc} {\hat{\mu }}_{YMRQ1}=\epsilon {ȳ}_{RSS}\left(\frac{\mu_{X}+q_{1}}{{\bar{x}}_{RSS}+q_{1}}\right), & & \end{array}$$ (39)

and


$$\begin{array}{ccc} {\hat{\mu }}_{YMRQ3}=\epsilon {ȳ}_{RSS}\left(\frac{\mu_{X}+q_{3}}{{\bar{x}}_{RSS}+q_{3}}\right). & & \end{array}$$ (40)

In addition, the authors also propose using ${ŷ}_{reg}$ to replace *ȳ* in and (40) and replacing *μ_X_* with ${\bar{x}}_{in}$. Also, ${\bar{x}}_{ir}$ replaced ${\bar{x}}_{RSS}$, resulting in two missing value estimators defined by and (42). These are called the adjusted regression-multivariate ratio quartile1 imputation with a rank set sampling of Feng, Ni, and Zou (AR-MRQ1RSS) and the adjusted regression-multivariate ratio quartile3 imputation with a rank set sampling of Feng, Ni, and Zou (AR-MRQ3RSS),


$$\begin{array}{ccc} {ŷ}_{AR-MRQ1RSS}={ŷ}_{reg}[\epsilon_{1}\left(\frac{{\bar{x}}_{1n}+q_{1}}{{\bar{x}}_{1r}+q_{1}}\right)+\epsilon_{2}\left(\frac{{\bar{x}}_{2n}+q_{1}}{{\bar{x}}_{2r}+q_{1}}\right)], & & \end{array}$$ (41)

and


$$\begin{array}{ccc} {ŷ}_{AR-MRQ3RSS}={ŷ}_{reg}[\epsilon_{1}\left(\frac{{\bar{x}}_{1n}+q_{3}}{{\bar{x}}_{1r}+q_{3}}\right)+\epsilon_{2}\left(\frac{{\bar{x}}_{2n}+q_{3}}{{\bar{x}}_{2r}+q_{3}}\right)], & & \end{array}$$ (42)

where ${ŷ}_{AR-MRQ1RSS}$ was the adjusted regression-multivariate ratio quartile1 imputation estimate with a rank set sampling; ${ŷ}_{AR-MRQ3RRS}$ was the adjusted regression-multivariate ratio quartile3 imputation estimate with a rank set sampling; and *ϵ*_1_,*ϵ*_2_ were the weighted values, and *ϵ*_1_+*ϵ*_2_=1. In this case, we set *ϵ*_1_=0.10,0.30,0.50,0.70,0.90 and *ϵ*_2_=0.10,0.30,0.50,0.70,0.90. By trial and error, *ϵ*_1_=0.9 and *ϵ*_2_=0.10 were found to give the best estimation performance.

2.10.8 Adjusted regression-multivariate chain ratio quartile1,3 imputation with RSS of Lu (AR-MCRQ1,3RSS)

Lu (2013) proposed a multivariate chain ratio estimator [15],


$$\begin{array}{ccc} {\hat{\mu }}_{YMCR}={ȳ}_{RSS}{\left(\frac{\omega_{1}{\bar{x}}_{1n}+\omega_{2}{\bar{x}}_{2n}}{\omega_{1}{\bar{x}}_{1r}+\omega_{2}{\bar{x}}_{2r}}\right)}^{\alpha }. & & \end{array}$$ (43)

The authors propose a new adaptation of the multivariate chain ratio method [15]. This adaptation estimated the missing values by taking advantage of the 1st and 3rd quartiles of variables *X*_1_ and *X*_2_,


$$\begin{array}{ccc} {\hat{\mu }}_{YMCRQ1}={ȳ}_{RSS}{\left(\frac{\omega_{1}\left({\bar{x}}_{1n}+q_{1}\right)+\omega_{2}({\bar{x}}_{2n}+q_{1})}{\omega_{1}({\bar{x}}_{1r}+q_{1})+\omega_{2}({\bar{x}}_{2r}+q_{1})}\right)}^{\alpha }, & & \end{array}$$ (44)

and


$$\begin{array}{ccc} {\hat{\mu }}_{YMCRQ3}={ȳ}_{RSS}{\left(\frac{\omega_{1}\left({\bar{x}}_{1n}+q_{3}\right)+\omega_{2}({\bar{x}}_{2n}+q_{3})}{\omega_{1}({\bar{x}}_{1r}+q_{3})+\omega_{2}({\bar{x}}_{2r}+q_{3})}\right)}^{\alpha }. & & \end{array}$$ (45)

In addition, the authors also propose using ${ŷ}_{reg}$ to replace *ȳ* in and (45), resulting in two missing value estimators defined by and (47). These are called the adjusted regression-multivariate chain ratio quartile1 imputation with a rank set sampling of Lu (AR-MCRQ1RSS) and the adjusted regression-multivariate chain ratio quartile3 imputation with a rank set sampling of Lu (AR-MCRQ3RSS),


$$\begin{array}{ccc} {ŷ}_{AR-MCRQ1RSS}={ŷ}_{reg}{\left(\frac{\omega_{1}\left({\bar{x}}_{1n}+q_{1}\right)+\omega_{2}({\bar{x}}_{2n}+q_{1})}{\omega_{1}({\bar{x}}_{1r}+q_{1})+\omega_{2}({\bar{x}}_{2r}+q_{1})}\right)}^{\alpha }, & & \end{array}$$ (46)

and


$$\begin{array}{ccc} {ŷ}_{AR-MCRQ3RSS}={ŷ}_{reg}{\left(\frac{\omega_{1}\left({\bar{x}}_{1n}+q_{3}\right)+\omega_{2}({\bar{x}}_{2n}+q_{3})}{\omega_{1}({\bar{x}}_{1r}+q_{3})+\omega_{2}({\bar{x}}_{2r}+q_{3})}\right)}^{\alpha }, & & \end{array}$$ (47)

where ${ŷ}_{AR-MCRQ1RSS}$ was the adjusted regression-multivariate chain ratio quartile1 imputation estimate with a rank set sampling; ${ŷ}_{AR-MCRQ3SRS}$ was the adjusted regression-multivariate chain ratio quartile3 imputation estimate with a rank set sampling; *α* was any constant value; and *ω*_1_,*ω*_2_ were the weighted values, and *ω*_1_+*ω*_2_=1. We set *α*=0.10,0.30,0.50,0.70,0.90; *ω*_1_=0.10,0.30,0.50,0.70,0.90, and *ω*_2_=0.10,0.30,0.50,0.70,0.90. The experiment showed that *α* = 0 . 10, *ω*_1_=0.10, and *ω*_2_=0.90 gave the best estimation performance.

2.11 The true value of the dependent variable (*y*) generated from Sect 2.5 and the estimated value (or predicted value) of the dependent variable (${ŷ}_{i}$) obtained from Sect 2.10 were used to calculate the mean squared error and the mean absolute percentage error.

2.12 Comparison of the eight regression-ratio imputation methods was based on mean squared error and mean absolute percentage error.

2.12.1 Mean Square Error (MSE) [1, 27–29].


$$\begin{array}{ccc} MSE=\frac{1}{n}\overset{n}{\underset{i=1}{\sum }}{\left(y_{i}-{ŷ}_{i}\right)}^{2}, & & \end{array}$$ (48)

where *y_i_* was the actual value of the i th dependent variable; ${ŷ}_{i}$ was the i th predicted value of the dependent variable; and MSE was the mean square error of the i th predicted value of the dependent variable.

2.12.2 Mean Absolute Percentage Error (MAPE) [1, 17].


$$\begin{array}{ccc} MAPE=\frac{1}{n}\overset{n}{\underset{i=1}{\sum }}\frac{\left|y_{i}-\hat{y_{i}}\right|}{\left|y_{i}\right|}X100, & & \end{array}$$ (49)

where MAPE was the mean absolute percentage error of the predicted value of the dependent variable.

2.13 R Studio Version 4.2.1 program was used to simulate each experimental scenario. For each sampling method, the simulation was repeated for 1,000 cycles [1, 17].

2.14 The actual data collected for multiple regression analysis are the following.

Two precious metal loss data set, gold and platinum were collected. The loss was in the production process of a jewelry company. The three independent variables were the total weight of the specimen (*X*_1_), the total output weight of the specimen (*X*_2_), and the recovery value (*X*_3_). The dependent variable was the loss of precious metals (*Y *). The sample size used was 12 sample units.

## 3. Results

### 3.1. Results of a study on simulated data

Mean square error for various simulation scenarios are the following: error variances of 1, 3, 5, 7, and 9; sample sizes of 20, 40, 60, 80, 100, 120, 200, and 500; and missing value percentages of 5, 10, 15, 20, 30 and 40%, with SRS in missing value estimation methods as shown in Tables 2, 3, 4, 5 and 6.

**Table 2:** Mean square errors for the scenario where error variance was 1; sample sizes of 20, 40, 60, 80, 100, 120, 200, and 500; and missing value percentages of 5, 10, 15, 20, 30, and 40% with SRS in missing value estimation methods.

Sample sizes	Missing values	Listwise delition	Missing value estimation methods							
			R-RQ1	R-RQ3	AR- CRQ1	AR-CRQ3	AR-MRQ1	AR-MRQ3	AR-MCRQ1	AR-MCRQ3
20	5%	1.0668	1.0045	1.0044	1.0045	1.0044	**0.9952**	0.9952	1.0715	**1.0693**
	10%	1.0768	1.0015	1.0019	1.0018	1.0021	**0.9951**	0.9951	**1.1698**	**1.1655**
	15%	1.0960	1.0130	1.0141	1.0136	1.0147	**1.0076**	1.0076	**1.2620**	**1.2555**
	20%	1.1148	1.0265	1.0269	1.0267	1.0274	**1.0240**	**1.0239**	**1.3676**	**1.3593**
	30%	1.1380	1.0741	1.0703	1.0716	1.0692	1.0669	1.0669	1.5727	**1.5604**
	40%	1.1416	1.1249	1.1069	1.1131	1.0994	1.0848	1.0848	1.7653	**1.7482**
40	5%	1.0807	1.0135	1.0137	1.0136	1.0137	**1.0077**	**1.0077**	1.0898	**1.0877**
	10%	1.1067	1.0174	1.0181	1.0178	1.0185	**1.0129**	1.0129	**1.1867**	**1.1823**
	15%	1.1101	1.0113	1.0122	1.0118	1.0127	**1.0093**	1.0093	**1.2767**	**1.2701**
	20%	1.1482	1.0498	1.0501	1.0499	1.0505	**1.0488**	**1.0488**	**1.3978**	**1.3894**
	30%	1.1532	1.0974	1.0918	1.0937	1.0897	1.0866	1.0866	**1.6051**	**1.5926**
	40%	1.1510	1.1431	1.1233	1.1299	1.1147	1.0989	1.0989	**1.7815**	**1.7646**
60	5%	1.0785	1.0122	1.0125	1.0124	1.0126	**1.0073**	1.0073	**1.0899**	**1.0877**
	10%	1.1018	1.0140	1.0148	1.0145	1.0152	**1.0105**	1.0105	**1.1816**	**1.1773**
	15%	1.1249	1.0322	1.0329	1.0326	1.0333	**1.0309**	1.0309	**1.2940**	**1.2876**
	20%	1.1425	1.0510	1.0508	1.0508	1.0510	**1.0501**	**1.0501**	**1.3978**	**1.3894**
	30%	1.1565	1.1040	1.0977	1.0998	1.0953	1.0917	1.0917	**1.6159**	**1.6034**
	40%	1.1531	1.1556	1.1342	1.1412	1.1247	1.1055	1.1055	**1.7997**	**1.7826**
80	5%	1.0666	1.0031	1.0033	1.0032	1.0034	**0.9990**	0.9990	1.0866	**1.0844**
	10%	1.1029	1.0149	1.0157	1.0154	1.0161	**1.0115**	1.0115	**1.1839**	**1.1795**
	15%	1.1171	1.0163	1.0171	1.0167	1.0176	**1.0153**	1.0153	**1.2799**	**1.2735**
	20%	1.1349	1.0391	1.0389	1.0389	1.0390	**1.0384**	**1.0384**	**1.3888**	**1.3804**
	30%	1.1531	1.1056	1.0989	1.1011	1.0963	1.0924	1.0924	**1.6175**	**1.6051**
	40%	1.1564	1.1598	1.1382	1.1453	1.1286	1.1090	1.1090	**1.8004**	**1.7834**
100	5%	1.0750	1.0085	1.0088	1.0087	1.0089	**1.0045**	1.0045	**1.0894**	**1.0872**
	10%	1.1002	1.0124	1.0131	1.0128	1.0135	**1.0096**	1.0096	**1.1834**	**1.1791**
	15%	1.1226	1.0202	1.0210	1.0207	1.0216	**1.0192**	1.0192	**1.2774**	**1.2710**
	20%	1.1357	1.0391	1.0391	1.0390	1.0393	**1.0386**	1.0386	**1.3874**	**1.3791**
	30%	1.1512	1.1002	1.0936	1.0958	1.0911	1.0874	1.0874	**1.6094**	**1.5970**
	40%	1.1621	1.1665	1.1452	1.1521	1.1357	1.1168	1.1168	**1.8004**	**1.7837**
120	5%	1.0713	1.0042	1.0045	1.0043	1.0046	**1.0002**	1.0002	**1.0845**	**1.0823**
	10%	1.1019	1.0149	1.0155	1.0152	1.0159	**1.0126**	1.0126	**1.1897**	**1.1854**
	15%	1.1217	1.0238	1.0245	1.0242	1.0249	**1.0231**	1.0231	**1.2841**	**1.2778**
	20%	1.1364	1.0410	1.0408	1.0408	1.0409	**1.0404**	**1.0404**	**1.3885**	**1.3802**
	30%	1.1554	1.1072	1.1003	1.1026	1.0976	1.0936	1.0936	**1.6220**	**1.6095**
	40%	1.1496	1.1621	1.1400	1.1472	1.1300	1.1087	1.1087	**1.8017**	**1.7849**
200	5%	1.0782	1.0147	1.0150	1.0148	1.0151	1.0111	1.0112	**1.0973**	**1.0951**
	10%	1.1011	1.0073	1.0081	1.0078	1.0086	1.0047	1.0047	**1.1768**	**1.1725**
	15%	1.1199	1.0203	1.0210	1.0207	1.0215	1.0197	1.0197	**1.2777**	**1.2714**
	20%	1.1436	1.0495	1.0490	1.0491	1.0491	1.0488	1.0488	**1.4027**	**1.3943**
	30%	1.1558	1.1041	1.0972	1.0995	1.0945	1.0907	1.0907	**1.6154**	**1.6029**
	40%	1.1604	1.1693	1.1470	1.1542	1.1369	1.1157	1.1157	**1.8083**	**1.7915**
500	5%	1.0736	1.0066	1.0070	1.0069	1.0072	1.0033	1.0033	**1.0883**	**1.0861**
	10%	1.1072	1.0125	1.0133	1.0130	1.0138	1.0102	1.0102	**1.1847**	**1.1804**
	15%	1.1264	1.0247	1.0254	1.0251	1.0260	1.0242	1.0242	**1.2844**	**1.2781**
	20%	1.1410	1.0465	1.0461	1.0462	1.0462	1.0460	1.0460	**1.3967**	**1.3883**
	30%	1.1493	1.1008	1.0935	1.0960	1.0906	1.0863	1.0863	**1.6152**	**1.6028**
	40%	1.1625	1.1697	1.1472	1.1544	1.1370	1.1160	1.1161	**1.8098**	**1.7929**

Bold values indicate the best method from comparing the four methods with SRS. The listwise deletion method is a control condition.

**Table 3:** Mean square errors for the scenario where error variance was 3; sample sizes of 20, 40, 60, 80, 100, 120, 200, and 500; and missing value percentages of 5, 10, 15, 20, 30, and 40% with SRS in missing value estimation methods.

Sample	Missing	Listwise	Missing value estimation methods							
sizes	values	delition	R-RQ1	R-RQ3	AR- CRQ1	AR-CRQ3	AR-MRQ1	AR-MRQ3	AR-MCRQ1	AR-MCRQ3
20	5%	3.0359	2.9873	2.9871	2.9872	2.9871	**2.9596**	2.9596	2.9648	**2.9631**
	10%	3.0744	2.9892	2.9918	2.9907	2.9931	**2.955**	2.955	**2.9832**	**2.9798**
	15%	3.1144	2.9918	2.9986	2.9959	3.0021	**2.9645**	2.9645	**3.0037**	**2.9987**
	20%	3.0696	2.9091	2.9205	2.9159	2.9266	**2.9025**	**2.9025**	**2.972**	**2.9653**
	30%	3.1096	2.8693	2.8901	2.882	2.9024	2.9294	2.9294	3.0255	**3.0159**
	40%	3.1651	2.8222	2.8448	2.836	2.8608	2.9695	2.9696	3.1041	**3.0909**
40	5%	3.0582	2.9893	2.9899	2.9897	2.9902	**2.9692**	**2.9692**	2.9888	**2.9871**
	10%	3.092	2.9843	2.9878	2.9863	2.9896	**2.9596**	2.9596	**2.9965**	**2.993**
	15%	3.1522	3.0213	3.0286	3.0256	3.0323	**3.0071**	3.0071	**3.0781**	**3.0729**
	20%	3.1167	2.9457	2.9577	2.9529	2.9642	**2.9502**	**2.9502**	**3.0289**	**3.0223**
	30%	3.1449	2.8948	2.9154	2.9073	2.9278	2.9655	2.9655	**3.0691**	**3.0594**
	40%	3.1574	2.7963	2.8191	2.8102	2.8356	2.9602	2.9603	**3.0835**	**3.0703**
60	5%	3.0767	3.0084	3.0094	3.009	3.0098	**2.9881**	2.9881	**2.9996**	**2.9979**
	10%	3.1042	2.9956	2.9995	2.9979	3.0015	**2.9729**	2.9729	**3.0033**	**3**
	15%	3.1057	2.9591	2.9671	2.9638	2.9712	**2.9471**	2.9471	**3.0027**	**2.9977**
	20%	3.1323	2.9514	2.9645	2.9591	2.9715	**2.9602**	**2.9602**	**3.0222**	**3.0157**
	30%	3.1348	2.8869	2.9071	2.8991	2.9194	2.9595	2.9596	**3.0661**	**3.0564**
	40%	3.1296	2.7752	2.7963	2.7878	2.8117	2.9345	2.9345	**3.078**	**3.0646**
80	5%	3.0844	3.0168	3.0177	3.0173	3.0182	**2.9977**	2.9977	3.0139	**3.0121**
	10%	3.109	3.0007	3.0047	3.0031	3.0067	**2.9795**	2.9795	**3.0133**	**3.0099**
	15%	3.1026	2.9578	2.9658	2.9625	2.9699	**2.9473**	2.9473	**3.0073**	**3.0022**
	20%	3.1388	2.9565	2.9692	2.964	2.9761	**2.9665**	**2.9665**	**3.0416**	**3.0351**
	30%	3.1396	2.8808	2.9011	2.893	2.9134	2.9556	2.9557	**3.0676**	**3.0578**
	40%	3.1638	2.7933	2.8151	2.8065	2.8312	2.96	2.9601	**3.0886**	**3.0754**
100	5%	3.0747	3.0037	3.0047	3.0043	3.0052	**2.9851**	2.9851	**2.9994**	**2.9977**
	10%	3.097	2.9872	2.9913	2.9896	2.9933	**2.9667**	2.9668	**3.0018**	**2.9985**
	15%	3.1132	2.9702	2.9784	2.975	2.9827	**2.9606**	2.9606	**3.012**	**3.0071**
	20%	3.1348	2.9528	2.9654	2.9602	2.9723	**2.9637**	2.9637	**3.0398**	**3.0332**
	30%	3.1458	2.8906	2.9113	2.903	2.9238	2.9676	2.9676	**3.0689**	**3.0593**
	40%	3.1528	2.7938	2.8149	2.8066	2.8305	2.9573	2.9574	**3.0879**	**3.0748**
120	5%	3.0503	2.9814	2.9824	2.982	2.9829	**2.964**	2.9641	**2.9826**	**2.9809**
	10%	3.0987	2.9875	2.9916	2.9899	2.9936	**2.9679**	2.9679	**3.0062**	**3.0028**
	15%	3.0983	2.9611	2.9691	2.9658	2.9732	**2.9523**	2.9523	**3.0077**	**3.0028**
	20%	3.144	2.9695	2.9822	2.977	2.9891	**2.9811**	**2.9811**	**3.0518**	**3.0454**
	30%	3.1569	2.9097	2.93	2.9219	2.9424	2.9862	2.9862	**3.096**	**3.0862**
	40%	3.135	2.7846	2.8046	2.7967	2.8196	2.9441	2.9442	**3.0906**	**3.0774**
200	5%	3.0633	2.9904	2.9916	2.9911	2.9921	2.9733	2.9733	**2.9919**	**2.9901**
	10%	3.0984	2.983	2.9872	2.9854	2.9893	2.964	2.964	**2.9963**	**2.993**
	15%	3.1228	2.975	2.9832	2.9798	2.9875	2.9673	2.9673	**3.0208**	**3.0159**
	20%	3.146	2.9647	2.9776	2.9724	2.9847	2.9778	2.9779	**3.0499**	**3.0434**
	30%	3.1576	2.9065	2.9266	2.9185	2.9389	2.9836	2.9837	**3.0931**	**3.0835**
	40%	3.1584	2.7994	2.8198	2.8117	2.8351	2.9632	2.9632	**3.1019**	**3.0887**
500	5%	3.0737	2.9991	3.0003	2.9998	3.0009	2.9817	2.9818	**2.996**	**2.9943**
	10%	3.105	2.9849	2.9894	2.9875	2.9916	2.9666	2.9667	**2.9983**	**2.995**
	15%	3.1402	2.9834	2.9921	2.9885	2.9966	2.9768	2.9769	**3.0264**	**3.0215**
	20%	3.1438	2.9565	2.9695	2.9642	2.9765	2.971	2.9711	**3.0435**	**3.037**
	30%	3.1521	2.893	2.9134	2.9053	2.9258	2.9728	2.973	**3.0807**	**3.071**
	40%	3.1561	2.7965	2.8167	2.8087	2.832	2.9617	2.9619	**3.1012**	**3.088**

**Table 4:** Mean square errors for the scenario where error variance was 5; sample sizes of 20, 40, 60, 80, 100, 120, 200 and 500; and missing value percentages of 5, 10, 15, 20, 30, and 40% with SRS in missing value estimation methods.

Sample	Missing	Listwise	Missing value estimation methods							
sizes	values	delition	R-RQ1	R-RQ3	AR- CRQ1	AR-CRQ3	AR-MRQ1	AR-MRQ3	AR-MCRQ1	AR-MCRQ3
20	5%	5.07	5.0133	5.0131	5.0132	5.013	**4.9689**	4.9689	4.916	**4.9148**
	10%	5.0714	4.9882	4.9921	4.9905	4.9941	**4.9344**	4.9344	**4.8476**	**4.8451**
	15%	5.1046	4.9505	4.9632	4.9579	4.9694	**4.9028**	4.9028	**4.7376**	**4.7339**
	20%	5.1802	4.9433	4.967	4.9572	4.9792	**4.933**	**4.933**	**4.7003**	**4.6957**
	30%	5.0881	4.6723	4.718	4.7002	4.744	4.8004	4.8004	4.4839	**4.477**
	40%	5.1664	4.4592	4.5265	4.5009	4.5681	4.8103	4.8103	4.3564	**4.3471**
40	5%	5.0605	4.998	4.9991	4.9986	4.9996	**4.9615**	**4.9615**	4.9077	**4.9064**
	10%	5.0876	4.9624	4.9688	4.9661	4.9719	**4.917**	4.917	**4.818**	**4.8154**
	15%	5.137	4.9601	4.975	4.9689	4.9826	**4.9311**	4.9311	**4.7733**	**4.7696**
	20%	5.1254	4.8718	4.8975	4.8871	4.911	**4.8831**	**4.8831**	**4.6574**	**4.6527**
	30%	5.1812	4.7267	4.7748	4.7557	4.8019	4.8809	4.8809	**4.5581**	**4.5513**
	40%	5.2041	4.4825	4.5474	4.5233	4.5888	4.8523	4.8523	**4.4208**	**4.4113**
60	5%	5.0534	4.9783	4.9799	4.9792	4.9806	**4.9418**	4.9419	**4.8798**	**4.8786**
	10%	5.0885	4.9505	4.9576	4.9546	4.9611	**4.9086**	4.9086	**4.8004**	**4.798**
	15%	5.1211	4.9384	4.9535	4.9473	4.9612	**4.9158**	4.9158	**4.7634**	**4.7597**
	20%	5.1182	4.8517	4.8774	4.8671	4.8911	**4.8696**	**4.8696**	**4.6526**	**4.6479**
	30%	5.1339	4.6857	4.7322	4.714	4.7588	4.842	4.8421	**4.5492**	**4.542**
	40%	5.1131	4.3994	4.4628	4.439	4.5032	4.7681	4.7682	**4.3559**	**4.3463**
80	5%	5.0823	4.9989	5.0007	4.9999	5.0016	**4.9629**	4.9629	4.9006	**4.8993**
	10%	5.1139	4.973	4.9805	4.9773	4.9842	**4.9324**	4.9324	**4.8197**	**4.8172**
	15%	5.1185	4.9131	4.9287	4.9222	4.9366	**4.893**	4.893	**4.7407**	**4.737**
	20%	5.1429	4.8677	4.8939	4.8833	4.9078	**4.8889**	**4.8889**	**4.6705**	**4.6658**
	30%	5.1603	4.7025	4.7501	4.7316	4.7775	4.8663	4.8663	**4.552**	**4.5451**
	40%	5.1406	4.4166	4.4802	4.4565	4.521	4.7922	4.7923	**4.3752**	**4.3655**
100	5%	5.05	4.9767	4.9785	4.9778	4.9794	**4.9444**	4.9444	**4.8926**	**4.8913**
	10%	5.0848	4.9574	4.9647	4.9617	4.9684	**4.9197**	4.9197	**4.8149**	**4.8125**
	15%	5.1508	4.9521	4.9679	4.9614	4.9759	**4.9336**	4.9337	**4.7755**	**4.772**
	20%	5.1363	4.8755	4.9006	4.8905	4.914	**4.8976**	4.8976	**4.7019**	**4.6972**
	30%	5.1292	4.6841	4.7308	4.7126	4.7577	4.8464	4.8465	**4.5425**	**4.5357**
	40%	5.1918	4.4588	4.5242	4.4999	4.566	4.845	4.845	**4.3999**	**4.3906**
120	5%	5.0791	5.0062	5.0081	5.0073	5.009	**4.9724**	4.9724	**4.9113**	**4.9101**
	10%	5.1243	4.9862	4.9938	4.9906	4.9976	**4.9489**	4.9489	**4.8439**	**4.8414**
	15%	5.107	4.9138	4.9295	4.923	4.9375	**4.8967**	4.8968	**4.7442**	**4.7407**
	20%	5.1528	4.8842	4.9097	4.8994	4.9232	**4.9079**	**4.9079**	**4.7106**	**4.7059**
	30%	5.1519	4.6843	4.7329	4.7139	4.7609	4.8542	4.8543	**4.535**	**4.528**
	40%	5.121	4.4068	4.4694	4.4462	4.5097	4.7816	4.7816	**4.3696**	**4.3601**
200	5%	5.0898	5.0105	5.0125	5.0116	5.0134	4.9805	4.9805	**4.9335**	**4.9322**
	10%	5.1154	4.9796	4.9873	4.9841	4.9911	4.9444	4.9444	**4.8403**	**4.8378**
	15%	5.136	4.9408	4.9571	4.9504	4.9654	4.9255	4.9256	**4.7621**	**4.7586**
	20%	5.1414	4.869	4.8948	4.8844	4.9085	4.8953	4.8954	**4.6997**	**4.6949**
	30%	5.1534	4.6887	4.7362	4.7176	4.7635	4.8576	4.8577	**4.5507**	**4.5437**
	40%	5.1211	4.4016	4.464	4.4408	4.5042	4.7784	4.7786	**4.3747**	**4.365**
500	5%	5.0789	5.0009	5.0031	5.0022	5.0041	4.9703	4.9704	**4.9151**	**4.9138**
	10%	5.1268	4.9898	4.9978	4.9945	5.0018	4.9562	4.9563	**4.8494**	**4.8469**
	15%	5.1254	4.9246	4.9411	4.9343	4.9496	4.9117	4.9118	**4.7487**	**4.7452**
	20%	5.1459	4.8709	4.8972	4.8866	4.9112	4.9004	4.9006	**4.6939**	**4.6892**
	30%	5.1658	4.7035	4.751	4.7325	4.7784	4.8758	4.876	**4.5697**	**4.5627**
	40%	5.1493	4.4198	4.4827	4.4594	4.5234	4.8035	4.8038	**4.389**	**4.3794**

**Table 5:** Mean square errors for the scenario where error variance was 7; sample sizes of 20, 40, 60, 80, 100, 120, 200 and 500; and missing value percentages of 5, 10, 15, 20, 30, and 40% with SRS in missing value estimation methods.

Sample	Missing	Listwise	Missing value estimation methods							
sizes	values	delition	R-RQ1	R-RQ3	AR- CRQ1	AR-CRQ3	AR-MRQ1	AR-MRQ3	AR-MCRQ1	AR-MCRQ3
20	5%	7.1099	7.0545	7.0544	7.0544	7.0543	**6.9904**	6.9904	**6.8634**	**6.8626**
	10%	7.0627	6.9451	6.9512	6.9486	6.9542	**6.8639**	6.8639	**6.6327**	**6.6311**
	15%	**6.9822**	6.7743	6.7921	6.7845	6.8008	**6.7091**	6.7091	**6.3703**	**6.3679**
	20%	7.0432	**6.7587**	6.7919	6.778	6.8088	**6.7451**	**6.7451**	**6.2704**	**6.2675**
	30%	7.2062	**6.5903**	6.6618	6.6342	6.7019	6.7901	6.7901	**6.0479**	**6.0436**
	40%	7.2022	**6.1587**	6.2664	6.2261	6.3316	6.6983	6.6983	**5.7046**	**5.6986**
40	5%	7.0911	7.029	7.0306	7.03	7.0314	**6.9766**	**6.9766**	**6.8514**	**6.8507**
	10%	7.0315	6.8913	6.9003	6.8965	6.9047	**6.8273**	6.8273	**6.5934**	**6.5917**
	15%	7.0286	6.8062	6.8274	6.8187	6.8382	**6.7648**	6.7648	**6.4116**	**6.4092**
	20%	7.1659	**6.821**	6.8582	6.8432	6.8777	**6.8379**	**6.838**	**6.3466**	**6.3437**
	30%	7.2192	**6.5727**	6.6469	6.6178	6.6884	6.8076	6.8077	**6.0744**	**6.0702**
	40%	7.1174	**6.0265**	6.1339	6.0951	6.201	6.609	6.609	**5.6049**	**5.599**
60	5%	7.1319	7.0543	7.0566	7.0556	7.0577	**7.0013**	7.0013	**6.8667**	**6.8659**
	10%	7.1549	7.0023	7.0124	7.0082	7.0174	**6.9412**	6.9412	**6.6945**	**6.6929**
	15%	7.1351	6.8942	6.9168	6.9075	6.9282	**6.8606**	6.8607	**6.4938**	**6.4916**
	20%	7.1836	**6.8282**	6.8673	6.8515	6.8878	**6.8557**	**6.8558**	**6.3498**	**6.347**
	30%	7.1156	**6.4784**	6.5524	6.5234	6.5939	6.7219	6.722	**6.001**	**5.9968**
	40%	7.12	**6.0185**	6.1273	6.0866	6.194	6.6098	6.6098	**5.6232**	**5.6171**
80	5%	7.0743	6.9964	6.9988	6.9978	7	**6.948**	**6.948**	**6.8254**	**6.8246**
	10%	7.0863	6.9479	6.9581	6.9539	6.9632	**6.892**	6.892	**6.6551**	**6.6535**
	15%	7.0993	6.8668	6.8895	6.8801	6.901	**6.8375**	6.8375	**6.4808**	**6.4786**
	20%	7.1073	**6.752**	6.7905	6.7749	6.8107	**6.7834**	**6.7835**	**6.3031**	**6.3003**
	30%	7.1654	**6.5152**	6.5895	6.5608	6.6317	6.766	6.7661	**6.0415**	**6.0372**
	40%	7.1406	**6.042**	6.1481	6.1091	6.2141	6.6311	6.6312	**5.6643**	**5.6582**
100	5%	7.1004	7.0167	7.0193	7.0182	7.0205	**6.9695**	6.9695	**6.8464**	**6.8456**
	10%	7.1808	7.0355	7.0463	7.0418	7.0517	**6.9796**	6.9796	**6.7283**	**6.7269**
	15%	7.1337	6.8828	6.9067	6.8968	6.9188	**6.8549**	6.855	**6.4746**	**6.4725**
	20%	7.1447	**6.7967**	6.8343	6.8191	6.8541	**6.83**	6.83	**6.3715**	**6.3685**
	30%	7.2589	**6.5751**	6.6519	6.6222	6.6954	6.8366	6.8367	**6.0833**	**6.0791**
	40%	7.1469	**6.0595**	6.166	6.1267	6.232	6.6507	6.6508	**5.676**	**5.6702**
120	5%	7.0733	6.9932	6.9958	6.9947	6.997	**6.9473**	6.9474	**6.8263**	**6.8255**
	10%	7.0984	6.944	6.9549	6.9504	6.9603	**6.89**	6.8901	**6.6475**	**6.6459**
	15%	7.1241	6.8901	6.9134	6.9038	6.9253	**6.8645**	6.8645	**6.4998**	**6.4976**
	20%	7.1579	**6.808**	6.8467	6.8311	6.8671	**6.8442**	**6.8442**	**6.3613**	**6.3585**
	30%	7.1285	**6.4725**	6.5468	6.5179	6.5889	6.7273	6.7274	**6.0123**	**6.0079**
	40%	7.1691	**6.0732**	6.1803	6.1411	6.247	6.6727	6.6728	**5.6867**	**5.6809**
200	5%	7.0838	6.9999	7.0027	7.0015	7.004	**6.9553**	6.9553	**6.8356**	**6.8348**
	10%	7.0504	6.8936	6.9049	6.9002	6.9106	**6.8412**	6.8413	**6.5932**	**6.5917**
	15%	**7.1243**	6.8789	6.9031	6.8932	6.9155	6.856	6.8561	**6.4773**	**6.4752**
	20%	7.121	6.7562	**6.7952**	6.7795	6.8158	6.7961	6.7962	**6.3241**	**6.3211**
	30%	7.1718	**6.5023**	6.5777	6.5484	6.6205	6.7647	6.7648	**6.0315**	**6.0274**
	40%	7.1896	**6.0734**	6.1806	6.1414	6.2476	6.6793	6.6795	**5.6957**	**5.6897**
500	5%	7.1041	7.0201	7.0231	7.0218	7.0246	**6.9759**	6.976	**6.8494**	**6.8486**
	10%	7.1159	6.9526	6.964	6.9593	6.9697	**6.9037**	6.9039	**6.662**	**6.6604**
	15%	7.1084	6.8592	6.8833	6.8734	6.8956	**6.8404**	6.8406	**6.4779**	**6.4756**
	20%	7.1465	**6.7808**	6.8203	6.8044	6.8413	6.8251	6.8255	**6.343**	**6.3402**
	30%	7.135	**6.4708**	6.5454	6.5166	6.5879	6.7355	6.7359	**6.0156**	**6.0113**
	40%	7.1735	**6.0708**	6.1775	6.1385	6.2442	6.679	6.6795	**5.6979**	**5.692**

**Table 6:** Mean square errors for the scenario where error variance was 9; sample sizes of 20, 40, 60, 80, 100, 120, 200 and 500; and missing value percentages of 5, 10, 15, 20, 25 and 30% with SRS in missing value estimation methods.

Sample	Missing	Listwise	Missing value estimation methods							
sizes	values	delition	R-RQ1	R-RQ3	AR- CRQ1	AR-CRQ3	AR-MRQ1	AR-MRQ3	AR-MCRQ1	AR-MCRQ3
20	5%	9.1658	9.1155	9.1154	9.1155	9.1153	**9.0266**	9.0266	8.8134	**8.8131**
	10%	9.1575	9.0251	9.0337	9.0301	9.0379	**8.9099**	8.9099	**8.5144**	**8.5137**
	15%	9.2442	9.024	9.0476	9.0379	9.0595	**8.9339**	8.9339	**8.3545**	**8.3536**
	20%	9.1821	8.8066	8.8512	8.833	8.8741	**8.789**	**8.789**	**8.0242**	**8.0231**
	30%	9.0696	8.2922	8.388	8.3517	8.442	8.5609	8.5609	7.379	**7.3778**
	40%	9.2087	7.7704	7.9247	7.8672	8.0169	8.5256	8.5257	6.9148	**6.913**
40	5%	9.0983	9.0152	9.0174	9.0165	9.0185	**8.9421**	**8.9421**	8.7381	**8.7377**
	10%	9.055	8.9144	8.9258	8.921	8.9314	**8.8319**	8.8319	**8.4716**	**8.4708**
	15%	9.1628	8.8982	8.9267	8.9149	8.941	**8.8432**	8.8433	**8.2802**	**8.2792**
	20%	9.1731	8.7396	8.79	8.7697	8.8164	**8.7631**	**8.7631**	**7.9761**	**7.9751**
	30%	9.1341	8.2855	8.3868	8.3471	8.443	8.6036	8.6036	**7.4512**	**7.4497**
	40%	9.064	7.6554	7.8016	7.7483	7.8909	8.4229	8.423	**6.9127**	**6.9104**
60	5%	9.1347	9.0503	9.0531	9.0519	9.0545	**8.9851**	8.9851	**8.7912**	**8.7908**
	10%	9.131	8.9562	8.9691	8.9637	8.9755	**8.8789**	8.8789	**8.507**	**8.5063**
	15%	9.1192	8.8321	8.8627	8.85	8.8781	**8.7865**	8.7866	**8.1963**	**8.1955**
	20%	9.2124	8.7744	8.826	8.8052	8.853	**8.811**	**8.811**	**8.0275**	**8.0266**
	30%	9.1888	8.3568	8.4571	8.418	8.513	8.6843	8.6844	**7.5453**	**7.544**
	40%	9.1625	7.7053	7.8571	7.8007	7.949	8.5092	8.5093	**6.9678**	**6.9654**
80	5%	9.0678	8.9816	8.9848	8.9835	8.9863	**8.9176**	8.9176	8.7206	**8.7203**
	10%	9.0619	8.8957	8.9095	8.9038	8.9163	**8.82**	8.8201	**8.4369**	**8.4362**
	15%	9.0816	8.7834	8.8135	8.8011	8.8288	**8.7444**	8.7444	**8.183**	**8.182**
	20%	9.1508	8.7015	8.7537	8.7326	8.781	**8.7443**	**8.7444**	**7.9726**	**7.9716**
	30%	9.1192	8.2634	8.3645	8.3255	8.4213	8.601	8.6011	**7.4572**	**7.4559**
	40%	9.0995	7.6533	7.8021	7.7476	7.8931	8.4547	8.4548	**6.9305**	**6.9282**
100	5%	9.0725	8.9862	8.9895	8.9881	8.9911	**8.925**	8.925	**8.7337**	**8.7333**
	10%	9.0981	8.9371	8.9507	8.9451	8.9576	**8.8661**	8.8661	**8.4946**	**8.4939**
	15%	9.1319	8.8225	8.854	8.841	8.87	**8.7856**	8.7856	**8.1968**	**8.196**
	20%	9.2084	8.7477	8.801	8.7795	8.8289	**8.7951**	8.7952	**8.0071**	**8.0062**
	30%	9.1818	8.306	8.4098	8.3696	8.468	8.6557	8.6559	**7.4841**	**7.4828**
	40%	9.1597	7.6988	7.8495	7.7946	7.942	8.5152	8.5154	**6.9602**	**6.9582**
120	5%	9.0252	8.9328	8.9363	8.9348	8.9379	**8.8723**	8.8723	**8.6807**	**8.6804**
	10%	9.0731	8.9116	8.926	8.92	8.9331	**8.8397**	8.8398	**8.4517**	**8.451**
	15%	9.0631	8.7726	8.8036	8.7909	8.8195	**8.7385**	8.7386	**8.1649**	**8.1641**
	20%	9.1355	8.69	8.7421	8.7211	8.7695	**8.7388**	**8.7389**	**7.9761**	**7.9751**
	30%	9.1794	8.3026	8.4051	8.3653	8.4627	8.6504	8.6506	**7.5096**	**7.5079**
	40%	9.1203	7.6519	7.8012	7.7468	7.893	8.4651	8.4652	**6.9317**	**6.9295**
200	5%	9.0581	8.9736	8.9773	8.9758	8.9791	8.9148	8.9148	**8.723**	**8.7227**
	10%	9.065	8.8903	8.9048	8.8988	8.9121	8.8228	8.8229	**8.4442**	**8.4436**
	15%	9.1	8.8009	8.8329	8.8198	8.8493	8.7705	8.7707	**8.1789**	**8.1782**
	20%	9.1051	8.6383	8.6923	8.6707	8.7208	8.6937	8.6939	**7.9083**	**7.9072**
	30%	9.1409	8.2658	8.3682	8.3286	8.4258	8.6185	8.6187	**7.475**	**7.4736**
	40%	9.1051	7.637	7.7856	7.7314	7.877	8.4522	8.4525	**6.9316**	**6.9293**
500	5%	9.0664	8.9758	8.9797	8.9781	8.9816	8.9183	8.9185	**8.7228**	**8.7225**
	10%	9.088	8.9034	8.9187	8.9123	8.9262	8.838	8.8382	**8.4495**	**8.4489**
	15%	9.0942	8.7907	8.8224	8.8094	8.8386	8.7658	8.7662	**8.1997**	**8.1988**
	20%	9.1933	8.736	8.7893	8.7679	8.8174	8.7958	8.7962	**8.0293**	**8.0283**
	30%	9.1229	8.2491	8.3518	8.3123	8.41	8.6098	8.6104	**7.4611**	**7.4596**
	40%	9.135	7.656	7.805	7.7509	7.897	8.4821	8.4827	**6.9543**	**6.9519**

**Table 7:** Mean absolute percentage errors for the scenario where error variance was 1; sample sizes of 20, 40, 60, 80, 100, 120, 200, and 500; and missing value percentages of 5, 10, 15, 20, 30, and 40% with SRS in missing value estimation methods.

Sample	Missing	Listwise	Missing value estimation methods							
sizes	values	delition	R-RQ1	R-RQ3	AR- CRQ1	AR-CRQ3	AR-MRQ1	AR-MRQ3	AR-MCRQ1	AR-MCRQ3
20	5%	357.0240	409.9905	409.9852	409.9874	409.9826	**409.4746**	409.4746	408.7430	**408.7323**
	10%	161.5125	145.8039	145.9150	145.8644	145.9650	**144.4226**	144.4227	**136.7948**	**136.8606**
	15%	195.3549	157.7165	157.9755	157.8668	158.1025	**156.6009**	156.6011	**146.8472**	**146.9226**
	20%	165.0662	151.6738	152.0258	151.8722	152.1969	**151.4220**	**151.4221**	**144.4557**	**144.4908**
	30%	150.5120	142.5156	143.0980	142.8585	143.4077	144.0135	144.0136	137.9127	**137.9066**
	40%	186.2471	149.8810	151.0837	150.6009	151.7644	155.3774	155.3776	142.6087	**142.6521**
40	5%	148.1718	142.1702	142.1816	142.1768	142.1871	**141.7056**	**141.7057**	140.7583	**140.7527**
	10%	332.7213	304.5201	304.6010	304.5674	304.6411	**303.8480**	303.8481	**300.8522**	**300.8560**
	15%	181.5588	172.8844	173.0961	173.0067	173.2009	**172.4254**	172.4256	**167.5177**	**167.5347**
	20%	208.7216	188.8416	189.2534	189.0736	189.4525	**189.0027**	**189.0029**	**181.8044**	**181.8443**
	30%	456.8280	358.6434	365.2686	362.2722	368.3630	376.5335	376.5363	**259.0079**	**260.5648**
	40%	990.4423	763.1088	764.3093	763.8936	765.0563	769.4204	769.4208	**754.9609**	**755.0088**
60	5%	278.8807	273.9939	274.0147	274.0060	274.0248	**273.4412**	273.4413	**271.5614**	**271.5667**
	10%	181.2294	170.4120	170.5020	170.4642	170.5461	**169.8508**	169.8511	**167.1597**	**167.1613**
	15%	890.4421	980.1620	980.8434	980.5731	981.1931	**978.9446**	978.9457	**952.7097**	**952.9737**
	20%	163.3203	157.5219	157.9053	157.7598	158.1143	**157.7790**	**157.7794**	**150.5265**	**150.5546**
	30%	547.7462	449.1250	454.3164	452.3545	457.1381	465.6979	465.7010	**364.3833**	**365.4964**
	40%	180.1938	60.5793	162.5951	161.8196	163.7451	170.2807	170.2816	**144.5299**	**144.7341**
80	5%	216.2313	199.7130	199.7292	199.7222	199.7368	**199.3701**	199.3702	198.7978	**198.7891**
	10%	209.7928	192.9521	193.1071	193.0453	193.1860	**192.0357**	192.0362	**184.9882**	**185.0391**
	15%	183.6553	171.5898	172.0165	171.8482	172.2376	**170.9791**	170.9799	**157.1203**	**157.2382**
	20%	167.7283	161.3135	161.9366	161.6819	162.2553	**161.7816**	**161.7825**	**147.8705**	**147.9872**
	30%	184.9231	174.2962	175.5289	175.0668	176.2204	178.3638	178.3648	**158.6837**	**158.8332**
	40%	332.4258	294.0354	296.0928	295.3839	297.3448	304.5341	304.5354	**276.4066**	**276.6173**
100	5%	419.3375	408.0261	408.0737	408.0525	408.0955	**407.0815**	407.0819	**402.0886**	**402.1348**
	10%	257.2928	226.9981	227.1258	227.0717	227.1880	**226.3215**	226.3220	**221.6669**	**221.6932**
	15%	201.5364	186.6597	187.1070	186.9322	187.3404	**186.0626**	186.0637	**171.3122**	**171.4387**
	20%	295.6438	278.7318	279.2318	279.0281	279.4889	**279.1539**	279.1548	**269.3752**	**269.4381**
	30%	211.8033	230.5168	233.7384	232.3486	235.3259	240.1146	240.1178	**185.5536**	**186.1916**
	40%	175.9687	151.8727	153.8010	153.1065	154.9501	161.6435	161.6450	**136.4368**	**136.6201**
120	5%	582.2329	592.3044	592.3369	592.3228	592.3521	**591.7209**	591.7212	**589.2743**	**589.2881**
	10%	510.5431	407.8482	408.0620	407.9718	408.1658	**406.7322**	406.7331	**396.5975**	**396.6896**
	15%	212.4910	266.3517	266.5953	266.4960	266.7193	**266.0657**	266.0664	**260.6137**	**260.6360**
	20%	234.3327	208.2549	209.4542	208.9768	210.0744	**209.3135**	**209.3159**	**177.5880**	**177.9164**
	30%	298.6394	277.1593	278.1674	277.7869	278.7335	280.5361	280.5374	**265.5794**	**265.6731**
	40%	229.5143	197.7167	200.3287	199.4316	201.9083	210.9107	210.9131	**173.7585**	**174.0799**
200	5%	186.1120	179.9175	179.9484	179.9353	179.9632	179.4110	179.4115	**177.3555**	**177.3640**
	10%	553.1657	483.2298	483.3648	483.3079	483.4306	482.5954	482.5964	**477.9329**	**477.9589**
	15%	258.5249	187.3378	187.7430	187.5703	187.9404	186.9562	186.9580	**175.6767**	**175.7732**
	20%	209.9852	194.7459	195.3659	195.1157	195.6862	195.3656	195.3676	**181.9872**	**182.0943**
	30%	248.5371	232.3416	233.6640	233.1352	234.3713	236.6477	236.6504	**216.4579**	**216.6256**
	40%	229.4328	206.4616	208.6076	207.8635	209.9099	217.5359	217.5392	**188.2519**	**188.4772**
500	5%	214.5356	214.4683	214.5014	214.4876	214.5175	213.9699	213.9711	**211.8940**	**211.9018**
	10%	213.5409	207.2012	207.3559	207.2924	207.4331	206.5123	206.5149	**200.7052**	**200.7433**
	15%	222.4501	206.2181	206.7256	206.5210	206.9842	205.8072	205.8129	**190.1129**	**190.2561**
	20%	196.8895	189.2922	189.7815	189.5879	190.0397	189.8393	189.8433	**180.4290**	**180.4858**
	30%	348.8762	361.3112	363.2223	362.4833	364.2601	367.6744	367.6841	**335.1211**	**335.4339**
	40%	262.2759	242.5020	246.5328	245.1165	248.9085	262.4728	262.4869	**203.8819**	**204.4593**

**Table 8:** Mean absolute percentage errors for the scenario where error variance was 3; sample sizes of 20, 40, 60, 80, 100, 120, 200, and 500; and missing value percentages of 5, 10, 15, 20, 30, and 40% with SRS in missing value estimation methods.

Sample	Missing	Listwise	Missing value estimation methods							
sizes	values	delition	R-RQ1	R-RQ3	AR- CRQ1	AR-CRQ3	AR-MRQ1	AR-MRQ3	AR-MCRQ1	AR-MCRQ3
20	5%	396.3733	422.8264	422.821	422.8233	422.8185	**421.9966**	421.9966	**419.1232**	**419.1328**
	10%	317.9099	307.931	308.1132	308.0258	308.1906	**305.8631**	305.8633	**291.9799**	**292.1166**
	15%	454.3682	434.9818	435.3811	435.2174	435.5794	**433.2143**	433.2146	**414.3878**	**414.5526**
	20%	254.2812	246.5314	247.3028	246.9751	247.6784	**246.107**	**246.1073**	**223.7344**	**223.931**
	30%	278.04	263.731	265.3545	264.641	266.1389	267.6247	267.6251	**240.4185**	**240.6636**
	40%	308.6102	262.9953	265.6628	264.6591	267.1647	274.707	274.7073	**238.0882**	**238.3747**
40	5%	266.4744	263.3253	263.35	263.3395	263.3617	**262.35**	**262.3501**	**257.7308**	**257.7634**
	10%	396.9011	372.1402	372.2718	372.2164	372.3357	**371.0895**	371.0898	**364.0336**	**364.0766**
	15%	316.0263	306.6974	307.0603	306.9088	307.2391	**305.9254**	305.9257	**293.7715**	**293.8603**
	20%	366.2173	354.3205	355.195	354.8227	355.622	**354.7096**	**354.7102**	**332.5847**	**332.7857**
	30%	330.9573	350.9561	354.4466	352.9707	356.1843	360.8083	360.8098	**298.3329**	**299.03**
	40%	2097.106	1859.346	1862.032	1861.061	1863.591	1872.011	1872.012	**1835.512**	**1835.786**
60	5%	349.1543	347.6565	347.6962	347.6794	347.7153	**346.6626**	346.6629	**341.4377**	**341.4786**
	10%	420.847	410.369	410.5406	410.4675	410.623	**409.3037**	409.3041	**400.7923**	**400.8544**
	15%	2305.664	2181.875	2182.851	2182.455	2183.341	**2180.143**	2180.144	**2140.96**	**2141.369**
	20%	278.576	271.2231	272.0026	271.7063	272.419	**271.7572**	**271.758**	**251.4015**	**251.5567**
	30%	318.7801	339.4706	342.4024	341.2751	343.9779	348.6231	348.6249	**294.6227**	**295.1527**
	40%	271.1867	248.4935	251.9869	250.711	253.9964	264.9377	264.9392	**216.2249**	**216.6472**
80	5%	397.8818	391.3425	391.3729	391.36	391.3874	**390.6676**	390.6679	**387.5581**	**387.5731**
	10%	335.6831	342.5455	342.7547	342.669	342.8587	**341.3178**	341.3185	**330.4685**	**330.5557**
	15%	328.6992	318.0031	318.7793	318.4818	319.1875	**316.8476**	316.8491	**286.7756**	**287.0621**
	20%	297.8024	280.6823	281.7066	281.2848	282.2214	**281.4747**	**281.4761**	**255.5061**	**255.7462**
	30%	340.2415	328.7046	331.1411	330.2098	332.4608	336.4465	336.4485	**292.7914**	**293.1976**
	40%	332.3621	331.4737	334.6426	333.5024	336.4863	346.6875	346.6893	**303.063**	**303.4233**
100	5%	362.0147	342.5573	342.6404	342.6025	342.6775	**340.9733**	340.9741	**331.1587**	**331.2629**
	10%	529.6419	500.5935	500.832	500.7322	500.9482	**499.3082**	499.3091	**487.1981**	**487.3011**
	15%	355.9075	360.7409	361.7501	361.3167	362.2337	**359.5388**	359.5412	**323.42**	**323.8102**
	20%	383.7087	381.2693	382.2711	381.8651	382.7811	**382.1268**	382.1285	**356.8614**	**357.0861**
	30%	366.27	377.8006	382.636	380.5946	385.0477	392.2941	392.2989	**306.6815**	**307.6715**
	40%	280.3766	245.8241	250.2469	248.6661	252.8106	266.9368	266.9399	**203.5037**	**204.1007**
120	5%	**509.8418**	526.3059	526.3526	526.3327	526.3749	**525.4324**	525.4329	**520.6026**	**520.6388**
	10%	456.7394	359.9311	360.2345	360.1058	360.3807	**358.3531**	358.3545	**342.5536**	**342.7033**
	15%	**587.473**	639.975	640.5456	640.3103	640.8294	**639.3123**	639.3139	**620.3177**	**620.4874**
	20%	358.1808	340.8846	342.6656	341.9535	343.5789	**342.4863**	**342.4899**	**293.1374**	**293.655**
	30%	415.3867	422.2891	424.7144	423.8027	426.0435	430.175	430.1779	**386.6414**	**387.0397**
	40%	356.1144	323.0185	329.0223	326.7624	332.369	350.6358	350.6406	**265.3109**	**266.2019**
200	5%	347.63	343.6034	343.6637	343.6386	343.6931	342.5732	342.5741	**336.4041**	**336.4548**
	10%	**645.4392**	689.0324	689.3093	689.1924	689.4434	687.7015	687.7035	**674.07**	**674.1946**
	15%	535.4903	557.1074	561.4151	559.4717	563.3826	553.4944	553.5148	**398.4252**	**400.3846**
	20%	359.2948	347.4576	348.534	348.1032	349.0875	348.5416	348.5451	**321.3754**	**321.6214**
	30%	406.0266	373.3965	376.0463	375.0006	377.4483	381.8634	381.8686	**335.7447**	**336.1896**
	40%	325.0766	304.4598	308.1997	306.8757	310.3892	322.6664	322.6716	**270.0919**	**270.5525**
500	5%	397.1003	387.9585	388.0188	387.9936	388.0481	387.0228	387.0251	**381.3677**	**381.4121**
	10%	422.2752	423.1033	423.3765	423.2655	423.5131	421.8598	421.8646	**408.4577**	**408.5738**
	15%	330.8041	319.7075	320.4169	320.1297	320.7752	319.1339	319.1419	**295.2779**	**295.5011**
	20%	386.4342	362.2357	363.2429	362.849	363.7705	363.3653	363.3734	**338.2637**	**338.48**
	30%	531.4369	520.1855	525.1385	523.2082	527.7705	536.2515	536.2755	**444.2679**	**445.2691**
	40%	560.9668	498.7476	511.2527	506.8453	518.4787	558.3848	558.4253	**368.1993**	**370.3058**

**Table 9:** Mean absolute percentage errors for the scenario where error variance was 5; sample sizes of 20, 40, 60, 80, 100, 120, 200 and 500; and missing value percentages of 5, 10, 15, 20, 30 and 40% with SRS in missing value estimation methods.

Sample	Missing	Listwise	Missing value estimation methods							
sizes	values	delition	R-RQ1	R-RQ3	AR- CRQ1	AR-CRQ3	AR-MRQ1	AR-MRQ3	AR-MCRQ1	AR-MCRQ3
20	5%	750.4612	693.8617	693.8554	693.8581	693.8525	**692.904**	692.9041	**689.2127**	**689.2302**
	10%	467.4288	452.7391	452.8756	452.8179	452.9412	**450.7353**	450.7355	**437.8525**	**437.9655**
	15%	607.3061	611.7478	612.1518	611.9836	612.3501	**610.017**	610.0173	**590.8113**	**590.9777**
	20%	382.3756	378.9746	380.1031	379.634	380.6615	**378.3465**	**378.347**	**341.5215**	**341.8813**
	30%	362.1214	347.8565	349.9756	349.0768	351.0247	353.0042	353.0047	**314.2307**	**314.597**
	40%	553.9524	553.7607	557.3752	556.0309	559.4029	569.4374	569.4379	**516.8758**	**517.3331**
40	5%	312.4886	312.9199	312.947	312.9358	312.9602	**311.8056**	**311.8057**	**306.1122**	**306.1558**
	10%	**639.3022**	658.7315	658.9114	658.8373	659.0001	**657.2774**	657.2777	**645.9464**	**646.0334**
	15%	463.3882	454.2956	454.8909	454.6412	455.1819	**453.0571**	453.0577	**430.0943**	**430.3065**
	20%	418.4452	402.5271	403.899	403.317	404.5676	**403.1531**	**403.1541**	**364.9162**	**365.3096**
	30%	522.2293	518.1702	526.5711	522.8652	530.5776	541.146	541.1494	**387.007**	**388.9696**
	40%	1267.334	1014.413	1018.478	1017.051	1020.849	1033.411	1033.413	**973.5068**	**974.0308**
60	5%	480.4941	473.2105	473.261	473.2403	473.2858	**471.8589**	471.8593	**464.0082**	**464.0746**
	10%	428.569	425.4493	425.6524	425.5673	425.7512	**424.1581**	424.1587	**413.0386**	**413.126**
	15%	1705.542	1589.961	1590.843	1590.486	1591.286	**1588.438**	1588.439	**1553.425**	**1553.777**
	20%	409.7187	397.9883	399.1266	398.6919	399.7308	**398.7725**	**398.7737**	**366.4374**	**366.7261**
	30%	561.1126	467.6295	471.6591	470.1273	473.8369	480.2411	480.2435	**402.7948**	**403.5847**
	40%	376.8161	342.9968	348.3318	346.3966	351.375	367.6689	367.6711	**289.5883**	**290.3519**
80	5%	445.5324	441.547	441.5882	441.5707	441.6078	**440.6295**	440.6298	**435.6045**	**435.6398**
	10%	552.3313	539.5132	539.7766	539.6675	539.9061	**537.9735**	537.9743	**523.3188**	**523.4479**
	15%	401.4017	393.0985	394.1194	393.7143	394.6414	**391.6475**	391.6495	**351.6491**	**352.0527**
	20%	408.7774	386.9152	388.5722	387.9032	389.4145	**388.1856**	**388.1879**	**341.7282**	**342.2018**
	30%	389.7421	368.7795	371.8356	370.7044	373.5209	378.6026	378.605	**320.6346**	**321.1863**
	40%	433.04	419.5373	424.6457	422.7895	427.5593	443.3487	443.3515	**369.7394**	**370.4442**
100	5%	743.1029	755.3779	755.5042	755.4452	755.5592	**752.9456**	752.9468	**736.8654**	**737.0511**
	10%	643.3278	656.4616	656.7896	656.6526	656.9494	**654.6904**	654.6916	**636.5673**	**636.7396**
	15%	474.185	440.3931	441.6299	441.0897	442.2129	**438.9609**	438.9638	**394.0767**	**394.578**
	20%	871.2665	864.3896	865.7652	865.2108	866.466	**865.5703**	865.5726	**828.2954**	**828.6572**
	30%	589.5559	497.0344	504.7744	501.4655	508.5787	519.9694	519.977	**380.2472**	**381.9385**
	40%	433.9685	364.4773	372.4158	369.665	377.0537	402.2187	402.224	**281.1981**	**282.4487**
120	5%	**1164.496**	1181.746	1181.817	1181.786	1181.85	**1180.445**	1180.446	**1172.303**	**1172.378**
	10%	**457.1772**	503.7146	504.1335	503.9612	504.3404	**501.4991**	501.501	**477.6804**	**477.918**
	15%	**532.175**	462.0255	462.7559	462.4563	463.1202	**461.1734**	461.1754	**435.1605**	**435.4066**
	20%	437.3237	413.6748	415.7798	414.9141	416.8338	**415.5819**	**415.5861**	**357.5116**	**358.1336**
	30%	756.5897	716.1417	719.7726	718.4079	721.7522	727.8758	727.88	**659.4141**	**660.0915**
	40%	450.8706	412.4233	417.0944	415.4411	419.8074	434.7024	434.7062	**367.0169**	**367.6471**
200	5%	446.1682	437.6951	437.7571	437.7307	437.7866	436.6481	436.6491	**430.2039**	**430.2583**
	10%	**700.1205**	749.8367	750.1511	750.0177	750.3024	748.3376	748.3399	**732.3101**	**732.4598**
	15%	493.4631	418.4324	419.7434	419.1822	420.3733	417.2309	417.237	**370.359**	**370.8765**
	20%	425.5464	412.6714	414.0686	413.5062	414.7818	414.0786	414.0832	**377.2279**	**377.5835**
	30%	612.7861	593.986	597.3977	596.0568	599.2017	604.8677	604.8744	**543.3552**	**543.9724**
	40%	471.3843	422.4269	428.4866	426.3523	432.0073	451.4099	451.418	**362.1695**	**363.0486**
500	5%	470.9242	460.2495	460.3311	460.2969	460.3706	458.9775	458.9806	**450.5111**	**450.5876**
	10%	459.4716	457.7515	458.1258	457.9734	458.3125	456.0438	456.0505	**436.2439**	**436.4307**
	15%	458.9581	438.2558	439.4288	438.9584	440.0242	437.2954	437.3087	**394.3314**	**394.7732**
	20%	565.0702	555.0596	556.4491	555.9068	557.1755	556.6179	556.6291	**519.5542**	**519.9027**
	30%	800.0828	766.4581	771.1895	769.3731	773.7293	781.9255	781.9484	**693.1935**	**694.13**
	40%	966.6159	821.1149	844.735	836.5476	858.4684	933.8673	933.9428	**565.5716**	**569.7543**

**Table 10:** Mean absolute percentage errors for the scenario where error variance was 7; sample sizes of 20, 40, 60, 80, 100, 120, 200 and 500; and missing value percentages of 5, 10, 15, 20, 25 and 40% with SRS in missing value estimation methods.

Sample	Missing	Listwise	Missing value estimation methods							
sizes	values	delition	R-RQ1	R-RQ3	AR- CRQ1	AR-CRQ3	AR-MRQ1	AR-MRQ3	AR-MCRQ1	AR-MCRQ3
20	5%	**726.4176**	751.6997	751.6923	751.6955	751.6888	**750.4558**	750.4559	**745.1667**	**745.1982**
	10%	370.051	381.2328	381.6068	381.4389	381.7766	**376.771**	376.7715	**342.5826**	**342.9597**
	15%	496.688	507.5724	508.314	508.0007	508.6727	**504.6236**	504.6241	**467.2244**	**467.6101**
	20%	437.6471	423.7297	424.849	424.3842	425.403	**423.1758**	**423.1763**	**386.6536**	**387.012**
	30%	403.1041	383.8389	386.6207	385.4362	387.9914	390.5875	390.5881	**337.1162**	**337.6714**
	40%	713.921	710.7703	715.4452	713.601	717.9474	730.1807	730.1813	**663.0215**	**663.676**
40	5%	424.043	420.741	420.7763	420.7615	420.7934	**419.3295**	**419.3297**	**411.5544**	**411.6235**
	10%	**478.5885**	460.0698	460.2943	460.2011	460.4041	**458.3072**	458.3076	**443.431**	**443.5615**
	15%	472.3669	453.4062	454.0093	453.7571	454.3047	**452.1407**	452.1413	**428.2866**	**428.507**
	20%	457.8988	438.3387	439.5158	439.0171	440.0903	**438.8387**	**438.8396**	**406.3538**	**406.6653**
	30%	679.4666	706.2843	711.9935	709.5707	714.8127	722.2921	722.2944	**615.6542**	**616.9101**
	40%	**5027.924**	5234.819	5239.465	5237.845	5242.178	5256.465	5256.466	**5186.624**	**5187.263**
60	5%	533.8603	534.2321	534.3058	534.2741	534.3406	**532.3139**	532.3144	**520.5379**	**520.6555**
	10%	502.5759	484.5606	484.8359	484.7211	484.9702	**482.7985**	482.7992	**466.3253**	**466.4758**
	15%	**2437.824**	2519.935	2521.066	2520.622	2521.648	**2517.948**	2517.949	**2470.512**	**2470.995**
	20%	453.0441	438.2988	439.5005	439.0319	440.1278	**439.133**	**439.1342**	**404.9905**	**405.2996**
	30%	1018.434	888.7062	905.8432	899.3943	915.125	943.254	943.264	**594.5933**	**598.5602**
	40%	446.7286	399.0657	404.8246	402.736	408.1035	425.6167	425.619	**340.7522**	**341.5877**
80	5%	541.0856	533.3067	533.3564	533.3351	533.38	**532.1881**	532.1885	**525.645**	**525.7**
	10%	435.0871	419.2938	419.6431	419.4966	419.8127	**417.2578**	417.2589	**396.8294**	**397.0263**
	15%	473.7543	451.6715	452.6873	452.2808	453.2029	**450.2224**	450.2244	**410.2552**	**410.6576**
	20%	454.2854	446.2367	448.31	447.4614	449.3505	**447.828**	**447.8308**	**388.632**	**389.2623**
	30%	448.827	431.4693	434.7924	433.5078	436.5671	441.8726	441.8752	**379.9776**	**380.586**
	40%	678.8082	665.4422	671.204	669.1992	674.5662	692.7515	692.7546	**606.405**	**607.2376**
100	5%	721.18	702.8082	702.9393	702.88	702.9982	**700.2079**	700.2092	**682.7908**	**682.9871**
	10%	810.4472	819.6088	820.0279	819.8487	820.2279	**817.3991**	817.4008	**793.6952**	**793.9399**
	15%	491.5639	472.4862	473.6803	473.1799	474.2642	**471.0325**	471.0353	**425.9613**	**426.4409**
	20%	594.4215	580.6282	582.5228	581.755	583.4824	**582.2525**	582.2557	**529.0572**	**529.6034**
	30%	478.7682	485.601	489.4081	487.8948	491.3984	497.4113	497.4151	**427.6206**	**428.3436**
	40%	456.1046	399.4679	406.9674	404.3423	411.3207	434.9045	434.9095	**321.5435**	**322.6979**
120	5%	**735.5547**	720.031	720.1208	720.0822	720.1632	**718.3842**	718.3852	**707.5568**	**707.6627**
	10%	**909.8261**	818.0973	818.7866	818.5038	819.1276	**814.4664**	814.4696	**773.2514**	**773.6944**
	15%	**576.4095**	510.5601	511.533	511.1318	512.0157	**509.4305**	509.4332	**473.3237**	**473.6878**
	20%	559.1264	527.0998	529.8384	528.7338	531.2296	**529.5389**	**529.5444**	**450.4983**	**451.3667**
	30%	788.557	722.9402	727.0996	725.5238	729.3508	736.2992	736.304	**656.9799**	**657.7865**
	40%	514.8918	450.7238	457.9915	455.4074	462.1729	484.8728	484.8786	**376.2669**	**377.3807**
200	5%	560.3821	559.4507	559.5467	559.5068	559.5935	557.8012	557.8027	**546.6495**	**546.7577**
	10%	**870.8277**	801.8949	802.239	802.0933	802.4049	800.2426	800.2451	**782.1006**	**782.2731**
	15%	546.5194	554.0526	555.6623	554.9659	556.4277	552.5758	552.5833	**494.2487**	**494.91**
	20%	487.4832	469.7138	471.208	470.6176	471.981	471.2288	471.2337	**430.5422**	**430.9327**
	30%	599.0403	558.966	563.4472	561.676	565.7992	573.1585	573.1672	**490.4093**	**491.2828**
	40%	529.7735	475.414	481.7525	479.5706	485.4759	505.9471	505.9555	**410.869**	**411.797**
500	5%	821.9789	830.0827	830.1782	830.1382	830.2245	828.5893	828.5929	**818.2699**	**818.3678**
	10%	549.0909	554.103	554.5535	554.3708	554.7786	552.0388	552.0469	**527.361**	**527.6041**
	15%	524.1909	521.7286	522.9995	522.4837	523.6385	520.7042	520.7186	**473.9867**	**474.4747**
	20%	514.5752	503.84	505.3422	504.7556	506.1266	505.5259	505.5379	**464.7822**	**465.1709**
	30%	760.8724	745.1218	751.5162	749.0342	754.9144	765.8352	765.8659	**644.3728**	**645.7092**
	40%	786.801	649.1244	662.591	657.8974	670.4082	713.4246	713.4679	**506.6956**	**508.959**

**Table 11:** Mean absolute percentage errors for the scenario where error variance was 9; sample sizes of 20, 40, 60, 80, 100, 120, 200 and 500; and missing value percentages of 5, 10, 15, 20, 30 and 40% with SRS in missing value estimation methods.

Sample	Missing	Listwise	Missing value estimation methods							
sizes	values	delition	R-RQ1	R-RQ3	AR- CRQ1	AR-CRQ3	AR-MRQ1	AR-MRQ3	AR-MCRQ1	AR-MCRQ3
20	5%	**603.6725**	570.9157	570.9088	570.9118	570.9056	**569.6877**	569.6878	**564.2018**	**564.2414**
	10%	449.4994	439.9817	440.2546	440.131	440.3776	**436.4939**	436.4942	**410.841**	**411.1112**
	15%	652.8954	621.6508	622.4758	622.1564	622.9035	**618.121**	618.1215	**572.6301**	**573.0768**
	20%	485.0231	481.6543	483.1272	482.5236	483.8624	**480.9152**	**480.9158**	**430.2197**	**430.7341**
	30%	468.5767	443.7278	447.4823	445.7304	449.1745	452.2996	452.3004	**383.966**	**384.7569**
	40%	747.7586	678.9428	684.2214	682.2591	687.1585	701.5145	701.5152	**620.911**	**621.7104**
40	5%	485.6536	485.941	485.9822	485.9648	486.002	**484.2917**	**484.2919**	**474.9915**	**475.0778**
	10%	**775.1819**	787.0131	787.2625	787.1584	787.384	**785.0115**	785.012	**767.8901**	**768.0496**
	15%	445.2454	441.4317	442.168	441.861	442.5291	**439.8805**	439.8813	**409.8568**	**410.1511**
	20%	668.0976	658.9483	660.5771	659.8865	661.3702	**659.6675**	**659.6687**	**612.6107**	**613.1039**
	30%	712.9221	726.7647	735.0896	731.5045	739.1452	749.8798	749.8832	**593.4361**	**595.3666**
	40%	**4318.264**	4490.4	4495.763	4493.863	4498.854	4515.066	4515.067	**4433.942**	**4434.723**
60	5%	511.6469	506.399	506.4667	506.4379	506.499	**504.6237**	504.6241	**493.7173**	**493.8234**
	10%	587.0515	572.5583	572.8415	572.7227	572.979	**570.7519**	570.7526	**553.587**	**553.7449**
	15%	**2672.749**	2743.382	2744.689	2744.154	2745.341	**2741.242**	2741.244	**2687.662**	**2688.236**
	20%	487.16	472.4826	473.8279	473.3066	474.5326	**473.3889**	**473.3903**	**433.9908**	**434.3586**
	30%	1364.768	1271.076	1275.14	1273.601	1277.337	1283.719	1283.722	**1204.347**	**1205.151**
	40%	490.7219	430.5766	437.3787	434.8645	441.1902	461.4485	461.4512	**361.2466**	**362.2772**
80	5%	715.9471	719.2025	719.2576	719.234	719.2837	**717.9766**	717.977	**710.6054**	**710.6704**
	10%	529.0046	516.5218	516.9212	516.7592	517.1207	**514.1495**	514.1508	**489.6722**	**489.9098**
	15%	516.9169	504.4084	505.5928	505.1309	506.2062	**502.6909**	502.6932	**454.3902**	**454.8814**
	20%	518.1969	497.3174	499.2072	498.4407	500.1627	**498.7775**	**498.7801**	**444.7729**	**445.3325**
	30%	522.3083	494.3585	498.877	497.1968	501.3505	508.839	508.8426	**420.1005**	**421.0098**
	40%	636.8692	609.9685	617.5	614.876	621.8816	645.4509	645.455	**530.4506**	**531.622**
100	5%	670.358	660.1401	660.2901	660.2218	660.3571	**657.1763**	657.1777	**637.0766**	**637.3092**
	10%	942.9645	956.5314	957.0261	956.8113	957.2589	**953.9479**	953.9498	**925.7504**	**926.0513**
	15%	630.7366	643.3765	645.2784	644.4414	646.1677	**641.1732**	641.1777	**569.8043**	**570.641**
	20%	785.7022	766.5944	768.6834	767.8261	769.7303	**768.399**	768.4025	**709.6924**	**710.3112**
	30%	823.4941	784.4692	797.2296	791.7518	803.4665	822.0874	822.0998	**588.0115**	**590.9292**
	40%	554.1761	473.2994	483.9635	480.2417	490.1458	523.5057	523.5127	**359.2338**	**360.9938**
120	5%	**846.3466**	828.597	828.6833	828.646	828.7239	**826.9885**	826.9895	**816.3837**	**816.4888**
	10%	**899.1226**	994.4357	995.0068	994.7668	995.2836	**991.4387**	991.4413	**957.8678**	**958.2258**
	15%	**689.8451**	739.3852	740.4189	739.9933	740.9319	**738.1888**	738.1917	**699.4441**	**699.8395**
	20%	568.7786	554.3665	556.4051	555.5826	557.4409	**556.2292**	**556.2333**	**498.5878**	**499.1883**
	30%	894.5608	878.6701	882.9708	881.3601	885.3164	892.5432	892.5482	**809.4516**	**810.2944**
	40%	586.1464	518.4325	527.8861	524.6473	533.4326	563.4489	563.4564	**416.9958**	**418.5304**
200	5%	562.7229	560.0087	560.1197	560.0739	560.174	558.0907	558.0925	**544.8119**	**544.943**
	10%	**1415.147**	1362.89	1363.285	1363.119	1363.475	1360.994	1360.997	**1339.548**	**1339.759**
	15%	866.8799	882.3174	888.8843	885.9287	891.889	876.8041	876.8352	**636.8848**	**639.9314**
	20%	575.0515	555.9877	557.76	557.0619	558.678	557.7898	557.7956	**508.2584**	**508.7507**
	30%	845.2901	812.0237	816.0417	814.4815	818.1783	824.8785	824.8863	**750.0429**	**750.809**
	40%	589.8235	532.9402	540.3069	537.7094	544.5651	567.8883	567.898	**457.7727**	**458.8919**
500	5%	653.686	657.5091	657.6161	657.5712	657.6677	655.8371	655.8412	**644.0459**	**644.1617**
	10%	645.0939	632.743	633.1995	633.0132	633.4265	630.665	630.6732	**605.6098**	**605.8576**
	15%	611.0222	597.5108	598.9397	598.3612	599.6593	596.3544	596.3706	**543.0303**	**543.5951**
	20%	694.4496	662.8342	665.3814	664.4174	666.7396	665.7081	665.7286	**591.3296**	**592.0963**
	30%	767.5472	731.8858	737.3545	735.2668	740.2963	749.791	749.8173	**645.4019**	**646.5143**
	40%	1164.152	948.3518	973.4455	964.6876	987.9658	1067.579	1067.659	**677.1057**	**681.5554**

As shown in Tables 2 and 3 for error variance of 1 and 3, most of the sample size and missing value percentage, the AR-MRQ1 method achieved the minimum mean square error, followed by AR-MRQ3. On the other hand, as can be observed in Tables 4, 5, and 6, for error variances of 5, 7, and 9, for every sample size and missing value percentage, the AR-MCRQ3 method achieved the minimum mean square error, followed by AR-MCRQ1.

Mean absolute percentage error for various simulation scenarios are the following: error variances of 1, 3, 5, 7, and 9; sample sizes of 20, 40, 60, 80, 100, 120, 200 and 500; and missing value percentages of 5, 10, 15, 20, 30, and 40%, with SRS in missing value estimation methods as shown in Tables 7, 8, 9, 10, and 11. As shown in Tables 7, 8, 9, 10, and 11, for error variance of 1, 3, 5, 7, and 9, every sample size and missing value percentage, AR-MCRQ1 method achieved the minimum mean absolute percentage error, followed by AR-MCRQ3.

Mean square error for various simulation scenarios are the following: error variances of 1, 3, 5, 7, and 9; sample sizes of 20, 40, 60, 80, 100, 120, 200, and 500; and missing value percentages of 5, 10, 15, 20, 30, and 40%, with RSS in missing value estimation methods as shown in Tables 12, 13, 14, 15, and 16.

**Table 12:** Mean square errors for the scenario where error variance was 1; sample sizes of 20, 40, 60, 80, 100, 120, 200 and 500; and missing value percentages of 5, 10, 15, 20, 30 and 40% with RSS in missing value estimation methods.

Sample	Missing	Listwise	Missing value estimation methods							
sizes	values	delition	R-RQ1	R-RQ3	AR- CRQ1	AR-CRQ3	AR-MRQ1	AR-MRQ3	AR-MCRQ1	AR-MCRQ3
20	5%	**1.0374**	1.0225	1.0224	1.0225	1.0224	**1.0129**	**1.0129**	**1.0757**	**1.0738**
	10%	1.0224	1.0012	1.0021	1.0017	1.0025	**0.9908**	0.9908	**1.1195**	**1.1157**
	15%	1.0415	1.0112	1.0135	1.0125	1.0146	**1.0037**	**1.0037**	**1.1947**	**1.1891**
	20%	1.0278	0.9918	0.9947	0.9934	0.9963	**0.9911**	**0.9911**	**1.2603**	**1.2528**
	30%	1.0528	1.0071	1.0085	1.0077	1.0100	**1.0151**	1.0151	**1.4203**	**1.4090**
	40%	1.0310	0.9788	0.9700	0.9727	0.9673	**0.9773**	0.9773	**1.5215**	**1.5065**
40	5%	1.0119	0.9999	1.0002	1.0001	1.0003	**0.9925**	**0.9925**	**1.0535**	**1.0517**
	10%	**1.0184**	1.0000	1.0012	1.0007	1.0019	**0.9923**	0.9923	**1.1214**	**1.1176**
	15%	1.0188	0.9956	0.9976	0.9968	0.9987	**0.9923**	**0.9923**	**1.1914**	**1.1859**
	20%	1.0122	0.9842	0.9865	0.9855	0.9880	**0.9855**	**0.9855**	**1.2570**	**1.2496**
	30%	1.0072	0.9577	0.9581	0.9577	0.9593	**0.9661**	0.9661	**1.3655**	**1.3545**
	40%	**1.0100**	0.9570	0.9468	0.9498	0.9434	**0.9544**	0.9544	**1.4808**	**1.4661**
60	5%	1.0119	1.0031	1.0034	1.0033	1.0036	**0.9963**	0.9963	**1.0569**	**1.0550**
	10%	1.0112	0.9975	0.9988	0.9983	0.9995	**0.9912**	0.9912	**1.1193**	**1.1157**
	15%	**1.0180**	0.9971	0.9993	0.9984	1.0005	**0.9944**	**0.9944**	**1.1939**	**1.1884**
	20%	1.0078	0.9760	0.9789	0.9777	0.9806	**0.9782**	**0.9782**	**1.2443**	**1.2368**
	30%	1.0194	0.9715	0.9714	0.9712	0.9724	**0.9794**	0.9794	**1.3812**	**1.3702**
	40%	1.0083	0.9570	0.9459	0.9493	0.9422	**0.9526**	0.9526	**1.4815**	**1.4667**
80	5%	1.0168	1.0086	1.0090	1.0088	1.0092	**1.0019**	1.0019	**1.0636**	**1.0617**
	10%	1.0130	1.0003	1.0016	1.0011	1.0023	**0.9940**	0.9940	**1.1229**	**1.1192**
	15%	1.0159	0.9938	0.9962	0.9952	0.9975	**0.9912**	0.9912	**1.1831**	**1.1776**
	20%	1.0145	0.9848	0.9873	0.9862	0.9889	**0.9870**	**0.9870**	**1.2522**	**1.2449**
	30%	1.0179	0.9671	0.9674	**0.9670**	0.9685	0.9764	0.9764	**1.3703**	**1.3593**
	40%	1.0162	0.9668	**0.9552**	0.9587	0.9512	0.9609	0.9609	**1.4908**	**1.4760**
100	5%	1.0048	0.9979	0.9983	0.9981	0.9985	**0.9918**	0.9918	**1.0539**	**1.0521**
	10%	1.0094	0.9975	0.9988	0.9983	0.9995	**0.9918**	0.9918	**1.1192**	**1.1155**
	15%	1.0054	0.9840	0.9863	0.9854	0.9876	**0.9818**	0.9818	**1.1756**	**1.1702**
	20%	1.0133	**0.9813**	0.9841	0.9829	0.9858	**0.9839**	0.9839	**1.2446**	**1.2373**
	30%	1.0040	**0.9538**	0.9539	0.9535	0.9550	0.9628	0.9628	**1.3546**	**1.3437**
	40%	1.0153	0.9667	0.9551	0.9586	**0.9510**	0.9606	0.9606	**1.4856**	**1.4709**
120	5%	**1.0106**	1.0044	1.0048	1.0046	1.0050	**0.9986**	0.9986	**1.0601**	**1.0583**
	10%	**1.0069**	0.9957	0.9970	0.9964	0.9976	**0.9904**	**0.9904**	**1.1190**	**1.1154**
	15%	**1.0128**	0.9932	0.9955	0.9945	0.9967	**0.9912**	0.9912	**1.1861**	**1.1806**
	20%	0.9996	0.9685	0.9713	0.9701	0.9730	**0.9712**	**0.9712**	**1.2320**	**1.2248**
	30%	1.0053	0.9538	0.9539	**0.9536**	0.9551	0.9632	0.9632	**1.3565**	**1.3455**
	40%	1.0100	0.9558	0.9446	0.9480	**0.9408**	0.9525	0.9525	**1.4699**	**1.4553**
200	5%	1.0065	1.0020	1.0024	1.0023	1.0026	**0.9968**	0.9968	**1.0599**	**1.0581**
	10%	**1.0007**	0.9909	0.9922	0.9917	0.9929	**0.9860**	0.9860	**1.1150**	**1.1114**
	15%	1.0047	0.9854	0.9876	0.9867	0.9888	**0.9836**	0.9836	**1.1794**	**1.1739**
	20%	0.9994	0.9690	0.9717	0.9705	0.9734	**0.9718**	0.9718	**1.2344**	**1.2271**
	30%	1.0070	0.9537	0.9539	**0.9535**	0.9551	0.9638	0.9638	**1.3521**	**1.3412**
	40%	1.0019	0.9503	**0.9385**	0.9420	0.9344	0.9451	0.9451	**1.4670**	**1.4523**
500	5%	1.0005	0.9976	0.9980	0.9978	0.9982	**0.9927**	0.9927	**1.0569**	**1.0550**
	10%	1.0054	0.9956	0.9969	0.9964	0.9976	**0.9909**	0.9909	**1.1208**	**1.1171**
	15%	1.0013	0.9815	0.9838	0.9828	0.9851	**0.9799**	0.9800	**1.1734**	**1.1680**
	20%	1.0075	**0.9762**	0.9789	0.9777	0.9807	0.9794	0.9794	**1.2384**	**1.2311**
	30%	1.0014	0.9492	0.9492	**0.9489**	0.9503	0.9589	0.9590	**1.3482**	**1.3372**
	40%	1.0060	0.9548	0.9429	0.9464	**0.9386**	0.9493	0.9493	**1.4677**	**1.4531**

Bold values indicate the best method from the comparison of the four methods with RSS.

**Table 13:** Mean square errors for the scenario where error variance was 3; sample sizes of 20, 40, 60, 80, 100, 120, 200 and 500; and missing value percentages of 5, 10, 15, 20, 30 and 40% with RSS in missing value estimation methods.

Sample	Missing	Listwise	Missing value estimation methods							
sizes	values	delition	R-RQ1	R-RQ3	AR- CRQ1	AR-CRQ3	AR-MRQ1	AR-MRQ3	AR-MCRQ1	AR-MCRQ3
20	5%	3.0436	3.0152	3.0152	3.0152	3.0151	**2.9864**	2.9864	2.9788	**2.9774**
	10%	3.0572	3.0201	3.0231	3.0218	3.0245	**2.9818**	2.9818	2.9639	**2.9611**
	15%	2.9942	2.9315	2.9388	2.9357	2.9425	**2.9052**	2.9052	2.9039	**2.8997**
	20%	3.0346	2.9292	2.9433	2.9374	2.9505	**2.9255**	**2.9255**	2.9158	**2.9103**
	30%	3.0473	**2.8243**	2.8501	2.8395	2.8646	2.8986	2.8986	2.9032	2.8948
	40%	3.0204	**2.6357**	2.6680	2.6554	2.6890	2.8227	2.8227	2.8150	2.8038
40	5%	2.9957	2.9780	2.9787	2.9784	2.9791	**2.9553**	**2.9553**	2.9509	**2.9495**
	10%	3.0000	2.9653	2.9693	2.9676	2.9713	**2.9376**	2.9376	2.9293	**2.9265**
	15%	2.9948	2.9278	2.9370	2.9332	2.9417	**2.9106**	2.9106	2.8957	**2.8916**
	20%	3.0166	2.9026	2.9179	2.9115	2.9259	**2.9102**	**2.9102**	2.8968	**2.8913**
	30%	3.0269	**2.7825**	2.8096	2.7988	2.8253	2.8729	2.8729	2.8549	2.8466
	40%	3.0436	**2.6369**	2.6690	2.6567	2.6904	2.8398	2.8398	2.8082	2.7973
60	5%	3.0090	2.9960	2.9970	2.9966	2.9975	**2.9743**	2.9743	2.9652	**2.9639**
	10%	2.9971	2.9635	2.9679	2.9661	2.9701	**2.9380**	2.9380	2.9255	**2.9228**
	15%	3.0108	2.9473	2.9564	2.9526	2.9611	**2.9343**	2.9344	2.9322	**2.9280**
	20%	3.0193	2.9011	2.9168	2.9103	2.9251	**2.9125**	**2.9125**	2.9042	**2.8985**
	30%	3.0257	**2.7737**	2.8010	2.7903	2.8171	2.8692	2.8693	2.8456	2.8374
	40%	2.9934	**2.5822**	2.6135	2.6015	2.6347	2.7878	2.7878	2.7624	2.7513
80	5%	2.9974	2.9861	2.9872	2.9867	2.9877	**2.9656**	2.9656	2.9593	**2.9580**
	10%	3.0075	2.9732	2.9779	2.9759	2.9802	**2.9487**	2.9487	2.9382	**2.9353**
	15%	3.0090	2.9431	2.9528	2.9488	2.9577	**2.9313**	2.9313	2.9189	**2.9148**
	20%	3.0009	2.8855	2.9011	2.8947	2.9094	**2.8985**	**2.8985**	2.8873	**2.8818**
	30%	3.0003	**2.7499**	2.7770	2.7662	2.7928	2.8460	2.8461	2.8297	2.8213
	40%	3.0102	**2.5947**	2.6259	2.6139	2.6471	2.8027	2.8027	2.7794	2.7682
100	5%	2.9963	2.9850	2.9861	2.9857	2.9867	**2.9646**	2.9646	2.9570	**2.9557**
	10%	3.0042	2.9728	2.9775	2.9755	2.9798	**2.9499**	2.9499	2.9389	**2.9362**
	15%	3.0136	2.9448	2.9546	2.9505	2.9596	**2.9338**	2.9339	2.9219	**2.9177**
	20%	3.0026	2.8839	2.8998	2.8933	2.9082	**2.8982**	2.8982	2.8799	**2.8745**
	30%	3.0042	**2.7524**	2.7796	2.7689	2.7956	2.8503	2.8504	2.8221	2.8140
	40%	3.0074	**2.5889**	2.6203	2.6083	2.6417	2.7992	2.7992	2.7687	2.7577
120	5%	3.0008	2.9914	2.9926	2.9921	2.9932	**2.9712**	2.9713	2.9615	**2.9602**
	10%	3.0019	2.9712	2.9757	2.9739	2.9780	**2.9497**	2.9497 48	2.9441	**2.9414**
	15%	3.0061	2.9359	2.9459	2.9418	2.9511	**2.9253**	2.9254	2.9107	**2.9065**
	20%	3.0059	2.8868	2.9027	2.8962	2.9112	**2.9018**	**2.9019**	2.8846	**2.8792**
	30%	3.0082	**2.7481**	2.7761	2.7650	2.7925	2.8493	2.8494	2.8185	2.8103
	40%	3.0012	**2.5815**	2.6131	2.6010	2.6345	2.7935	2.7935	2.7547	2.7438
200	5%	3.0032	2.9932	2.9945	2.9939	2.9951	2.9734	2.9734	2.9639	**2.9625**
	10%	3.0074	2.9765	2.9811	2.9792	2.9834	2.9559	2.9559	2.9511	**2.9483**
	15%	2.9955	2.9258	2.9358	2.9317	2.9410	2.9166	2.9166	2.9025	**2.8984**
	20%	2.9955	2.8743	2.8905	2.8839	2.8991	2.8910	2.8910	2.8730	**2.8675**
	30%	3.0040	**2.7464**	2.7738	2.7631	2.7901	2.8478	2.8479	2.8198	2.8116
	40%	3.0003	**2.5814**	2.6123	2.6005	2.6335	2.7930	2.7931	2.7612	2.7502
500	5%	3.0074	2.9991	3.0004	2.9998	3.0011	2.9804	2.9805	2.9743	**2.9730**
	10%	2.9993	2.9672	2.9722	2.9701	2.9746	2.9466	2.9467	2.9358	**2.9331**
	15%	3.0036	2.9335	2.9436	2.9395	2.9489	2.9257	2.9258	2.9100	**2.9059**
	20%	2.9929	2.8715	2.8876	2.8811	2.8963	2.8896	2.8898	2.8726	**2.8672**
	30%	2.9982	**2.7386**	2.7661	2.7553	2.7824	2.8421	2.8423	2.8123	2.8041
	40%	2.9924	**2.5700**	2.6011	2.5892	2.6224	2.7846	2.7848	2.7479	**2.7369**

**Table 14:** Mean square errors for the scenario where error variance was 5; sample sizes of 20, 40, 60, 80, 100, 120, 200 and 500; and missing value percentages of 5, 10, 15, 20, 30 and 40% with RSS in missing value estimation methods.

Sample	Missing	Listwise	Missing value estimation methods							
sizes	values	delition	R-RQ1	R-RQ3	AR- CRQ1	AR-CRQ3	AR-MRQ1	AR-MRQ3	AR-MCRQ1	AR-MCRQ3
20	5%	5.0711	5.0486	5.0485	5.0485	5.0485	**5.0014**	5.0014	4.9253	**4.9243**
	10%	5.0833	5.0433	5.0484	5.0462	5.0508	**4.9785**	4.9785	4.8089	**4.8072**
	15%	**2.9942**	5.0092	5.0235	5.0174	5.0305	**4.9572**	4.9572	4.7228	**4.7199**
	20%	4.9656	4.8056	4.8298	4.8196	4.8420	**4.7985**	**4.7985**	4.5367	**4.5329**
	30%	4.9580	**4.5395**	4.5930	4.5711	4.6219	4.6871	4.6871	4.2551	**4.2495**
	40%	4.9739	**4.2483**	4.3223	4.2937	4.3669	4.6243	4.6243	4.0785	**4.0709**
40	5%	4.9600	4.9390	4.9401	4.9396	4.9407	**4.9018**	**4.9018**	4.8327	**4.8317**
	10%	5.0206	4.9708	4.9777	4.9748	4.9811	**4.9231**	4.9232	4.7761	**4.7743**
	15%	5.0117	4.9051	4.9212	4.9145	4.9293	**4.8745**	4.8745	4.6487	**4.6460**
	20%	5.0357	4.8414	4.8690	4.8577	4.8833	**4.8552**	**4.8552**	4.5667	**4.5630**
	30%	5.0209	**4.5768**	4.6309	4.6095	4.6612	4.7498	4.7499	4.3145	**4.3089**
	40%	5.0459	**4.2739**	4.3493	4.3210	4.3960	4.6869	4.6869	4.0947	**4.0874**
60	5%	5.0170	4.9975	4.9992	4.9985	5.0000	**4.9597**	4.9597	4.8780	**4.8771**
	10%	5.0150	4.9615	4.9693	4.9660	4.9731	**4.9158**	4.9158	4.7593	**4.7574**
	15%	4.9993	4.8850	4.9017	4.8948	4.9103	**4.8606**	4.8606	4.6383	**4.6356**
	20%	4.9912	4.7922	4.8201	4.8087	4.8348	**4.8124**	**4.8125**	4.5317	**4.5279**
	30%	4.9690	**4.5187**	4.5726	4.5516	4.6032	4.6993	4.6993	4.2623	**4.2568**
	40%	4.9923	**4.2161**	4.2903	4.2627	4.3368	4.6340	4.6341	4.0528	**4.0454**
80	5%	5.0250	5.0101	5.0119	5.0111	5.0128	**4.9744**	4.9744	4.9001	**4.8991**
	10%	4.9893	4.9377	4.9454	4.9422	4.9492	**4.8963**	4.8964	4.7523	**4.7504**
	15%	5.0016	4.8845	4.9017	4.8946	4.9105	**4.8627**	4.8628	4.6361	**4.6334**
	20%	5.0137	4.8086	4.8375	4.8258	4.8527	**4.8327**	**4.8327**	4.5356	**4.5319**
	30%	5.0374	**4.5716**	4.6276	4.6057	4.6593	4.7615	4.7615	4.3053	**4.2997**
	40%	5.0519	**4.2559**	4.3317	4.3036	4.3792	4.6866	4.6867	4.0870	**4.0796**
100	5%	4.9946	4.9782	4.9801	4.9793	4.9810	**4.9435**	4.9435	4.8675	**4.8666**
	10%	4.9956	4.9441	4.9520	4.9487	4.9559	**4.9042**	4.9042	4.7573	**4.7555**
	15%	4.9684	4.8496	4.8673	4.8600	4.8762	**4.8295**	4.8295	4.5968	**4.5942**
	20%	4.9875	4.7809	4.8099	4.7982	4.8252	**4.8070**	4.8070	4.5095	**4.5059**
	30%	5.0313	**4.5690**	4.6241	4.6027	4.6556	4.7589	4.7590	4.3063	**4.3009**
	40%	4.9917	**4.1962**	4.2716	4.2436	4.3190	4.6278	4.6278	4.0290	**4.0216**
120	5%	5.0090	4.9946	4.9965	4.9957	4.9975	**4.9602**	4.9602	4.8830	**4.8821**
	10%	5.0158	4.9623	4.9705	4.9671	4.9745	**4.9222**	4.9223	4.7697	**4.7679**
	15%	5.0009	4.8816	4.8995	4.8921	4.9087	**4.8624**	4.8625	4.6311	**4.6284**
	20%	5.0228	4.8106	4.8403	4.8283	4.8560	**4.8386**	**4.8387**	4.5317	**4.5281**
	30%	5.0226	**4.5606**	4.6157	4.5943	4.6471	4.7516	4.7516	4.2997	**4.2943**
	40%	4.9806	**4.1899**	4.2644	4.2369	4.3114	4.6195	4.6196	4.0265	**4.0192**
200	5%	5.0232	5.0094	5.0115	5.0106	5.0125	4.9767	4.9767	4.9017	**4.9008**
	10%	4.9998	4.9477	4.9559	4.9525	4.9600	4.9103	4.9104	4.7636	**4.7618**
	15%	5.0208	4.9009	4.9187	4.9114	4.9279	4.8842	4.8843	4.6567	**4.6540**
	20%	5.0076	4.7998	4.8288	4.8171	4.8442	4.8297	4.8298	4.5419	**4.5381**
	30%	4.9620	**4.5005**	4.5551	4.5339	4.5863	4.6929	4.6930	4.2515	**4.2460**
	40%	4.9959	**4.2076**	4.2816	4.2542	4.3283	4.6375	4.6376	4.0465	**4.0392**
500	5%	5.0087	4.9952	4.9974	4.9965	4.9985	4.9636	4.9636	4.8889	**4.8880**
	10%	4.9972	4.9435	4.9520	4.9485	4.9562	4.9075	4.9077	4.7573	**4.7555**
	15%	4.9951	4.8747	4.8926	4.8853	4.9018	4.8607	4.8609	4.6368	**4.6341**
	20%	5.0088	4.7961	4.8257	4.8138	4.8414	4.8294	4.8296	4.5283	**4.5247**
	30%	4.9935	**4.5280**	4.5829	4.5616	4.6143	4.7246	4.7249	4.2787	**4.2732**
	40%	5.0199	**4.2188**	4.2936	4.2661	4.3410	4.6580	4.6584	4.0536	**4.0463**

**Table 15:** Mean square errors for the scenario where error variance was 7; sample sizes of 20, 40, 60, 80, 100, 120, 200, and 500; and missing value percentages of 5, 10, 15, 20, 30 and 40% with RSS in missing value estimation methods.

Sample	Missing	Listwise	Missing value estimation methods							
sizes	values	delition	R-RQ1	R-RQ3	AR- CRQ1	AR-CRQ3	AR-MRQ1	AR-MRQ3	AR-MCRQ1	AR-MCRQ3
20	5%	7.0695	7.0593	7.0592	7.0592	7.0592	**6.9882**	6.9882	6.8233	**6.8229**
	10%	6.9631	6.8924	6.8994	6.8964	6.9027	**6.8002**	6.8002	6.4964	**6.4955**
	15%	**7.0693**	6.9228	6.9434	6.9347	6.9536	**6.8483**	6.8483	6.3740	**6.3729**
	20%	7.0401	6.7909	6.8272	6.8121	6.8456	**6.7796**	**6.7796**	6.2163	**6.2144**
	30%	6.9827	**6.3827**	6.4620	6.4297	6.5046	6.5999	6.5999	5.7546	**5.7517**
	40%	7.0797	**5.9908**	6.1091	6.0643	6.1795	6.5724	6.5725	5.4332	**5.4295**
40	5%	6.9658	6.9448	6.9465	6.9457	6.9472	**6.8921**	**6.8921**	6.7583	**6.7577**
	10%	7.0406	6.9715	6.9818	6.9775	6.9868	**6.8987**	6.8988	6.5957	**6.5947**
	15%	7.0306	6.8693	6.8927	6.8830	6.9045	**6.8249**	6.8249	6.3813	**6.3800**
	20%	6.9718	6.6954	6.7351	6.7189	6.7556	**6.7150**	**6.7150**	6.1549	**6.1530**
	30%	6.9825	**6.3428**	6.4238	6.3919	6.4688	6.5987	6.5987	5.7377	**5.7350**
	40%	7.0371	**5.9162**	6.0328	5.9893	6.1035	6.5296	6.5296	5.4066	**5.4028**
60	5%	6.9832	6.9655	6.9677	6.9667	6.9688	**6.9142**	6.9142	6.7709	**6.7704**
	10%	7.0218	6.9521	6.9626	6.9582	6.9678	**6.8903**	6.8904	6.6076	**6.6067**
	15%	7.0291	6.8696	6.8937	6.8838	6.9059	**6.8344**	6.8345	6.4051	**6.4037**
	20%	6.9670	6.6782	6.7200	6.7030	6.7417	**6.7085**	**6.7085**	6.1321	**6.1302**
	30%	7.0818	**6.4360**	6.5162	6.4852	6.5614	6.7010	6.7011	5.8524	**5.8496**
	40%	7.0394	**5.8989**	6.0161	5.9730	6.0879	6.5301	6.5301	5.3841	**5.3805**
80	5%	6.9899	6.9697	6.9722	6.9711	6.9734	**6.9194**	6.9194	6.7732	**6.7728**
	10%	6.9777	6.9009	6.9123	6.9075	6.9179	**6.8393**	6.8393	6.5403	**6.5394**
	15%	7.0737	6.9145	6.9385	6.9286	6.9506	**6.8842**	6.8843	6.4678	**6.4664**
	20%	7.0118	6.7188	6.7608	6.7438	6.7828	**6.7537**	**6.7538**	6.1746	**6.1728**
	30%	6.9853	**6.3212**	6.4038	6.3716	6.4501	6.5975	6.5976	5.7268	**5.7241**
	40%	6.9988	**5.8673**	5.9827	5.9403	6.0536	6.4954	6.4955	5.3743	**5.3705**
100	5%	7.0047	6.9854	6.9881	6.9870	6.9895	**6.9345**	6.9345	6.7804	**6.7800**
	10%	7.0240	6.9497	6.9612	6.9564	6.9669	**6.8905**	6.8905	6.5914	**6.5906**
	15%	7.0274	6.8649	6.8896	6.8795	6.9022	**6.8364**	6.8365	6.4047	**6.4034**
	20%	7.0175	6.7189	6.7615	6.7443	6.7838	**6.7571**	6.7572	6.1720	**6.1703**
	30%	7.0203	**6.3592**	6.4412	6.4094	6.4874	6.6370	6.6371	5.7685	**5.7660**
	40%	7.0213	**5.8620**	5.9803	5.9367	6.0530	6.5077	6.5079	5.3489	**5.3454**
120	5%	7.0325	7.0119	7.0147	7.0135	7.0161	**6.9622**	6.9623	6.8126	**6.8121**
	10%	7.0585	6.9864	6.9978	6.9931	7.0035	**6.9297**	6.9298	6.6410	**6.6401**
	15%	7.0147	6.8496	6.8746	6.8643	6.8872	**6.8227**	6.8228	6.3931	**6.3918**
	20%	7.0363	6.7418	6.7839	6.7670	6.8061	**6.7815**	**6.7816**	6.2045	**6.2028**
	30%	6.9940	**6.3251**	6.4079	6.3758	6.4546	6.6075	6.6076	5.7314	**5.7289**
	40%	6.9648	**5.8211**	5.9368	5.8945	6.0082	6.4585	6.4587	5.3270	**5.3232**
200	5%	7.0262	7.0055	7.0086	7.0073	7.0101	6.9568	6.9568	6.8020	**6.8016**
	10%	6.9985	6.9262	6.9376	6.9328	6.9432	6.8737	6.8738	6.5958	**6.5949**
	15%	7.0163	6.8484	6.8737	6.8634	6.8867	6.8248	6.8249	6.3940	**6.3927**
	20%	6.9913	6.6910	6.7338	6.7165	6.7564	6.7351	6.7352	6.1578	**6.1560**
	30%	7.0136	**6.3414**	6.4240	6.3921	6.4707	6.6278	6.6280	5.7588	**5.7561**
	40%	7.0431	**5.8783**	5.9961	5.9530	6.0688	6.5305	6.5307	5.3722	**5.3687**
500	5%	6.9748	6.9558	6.9589	6.9576	6.9605	6.9105	6.9106	6.7653	**6.7648**
	10%	6.9991	6.9226	6.9348	6.9297	6.9408	6.8705	6.8707	6.5790	**6.5781**
	15%	7.0152	6.8441	6.8699	6.8594	6.8831	6.8239	6.8242	6.3879	**6.3866**
	20%	7.0106	6.7061	6.7493	6.7320	6.7721	6.7546	6.7550	6.1739	**6.1721**
	30%	6.9618	**6.2852**	6.3681	6.3361	6.4151	6.5772	6.5777	5.7068	**5.7041**
	40%	6.9924	**5.8204**	5.9382	5.8952	6.0111	6.4797	6.4802	5.3187	**5.3151**

**Table 16:** Mean square errors for the scenario where error variance was 9; sample sizes of 20, 40, 60, 80, 100, 120, 200, and 500; and missing value percentages of 5, 10, 15, 20, 30 and 40% with RSS in missing value estimation methods.

Sample	Missing	Listwise	Missing value estimation methods							
sizes	values	delition	R-RQ1	R-RQ3	AR- CRQ1	AR-CRQ3	AR-MRQ1	AR-MRQ3	AR-MCRQ1	AR-MCRQ3
20	5%	9.0487	9.0347	9.0346	9.0347	9.0346	**8.9512**	8.9512	8.7425	**8.7424**
	10%	9.1216	9.0419	9.0511	9.0471	9.0554	**8.9231**	8.9231	**8.4792**	**8.4792**
	15%	**9.1263**	8.9445	8.9705	8.9596	8.9835	**8.8513**	8.8514	**8.1871**	**8.1872**
	20%	9.1082	8.7787	8.8269	8.8068	8.8512	**8.7644**	**8.7644**	**7.9146**	**7.9147**
	30%	9.0519	**8.2758**	8.3804	8.3380	8.4363	8.5596	8.5596	7.2954	**7.2954**
	40%	9.0489	**7.6315**	7.7889	7.7305	7.8826	8.3975	8.3975	**6.7114**	**6.7115**
40	5%	9.0618	9.0374	9.0397	9.0387	9.0407	**8.9652**	**8.9652**	**8.7460**	**8.7460**
	10%	9.0585	8.9675	8.9805	8.9750	8.9868	**8.8758**	8.8758	8.4377	**8.4376**
	15%	9.1463	8.9434	8.9732	8.9610	8.9883	**8.8863**	8.8863	8.2465	**8.2465**
	20%	9.0675	8.7005	8.7543	8.7322	8.7820	**8.7272**	**8.7272**	**7.8576**	**7.8577**
	30%	8.9323	**8.1158**	8.2214	8.1798	8.2795	8.4463	8.4463	7.2014	**7.2013**
	40%	9.0316	**7.5425**	7.7022	7.6434	7.7986	8.3686	8.3687	6.6802	**6.6799**
60	5%	9.1024	9.0762	9.0791	9.0779	9.0805	**9.0099**	9.0099	**8.7948**	**8.7949**
	10%	9.0273	8.9356	8.9501	8.9440	8.9571	**8.8504**	8.8504	**8.4028**	**8.4028**
	15%	9.1263	8.9162	8.9483	8.9351	8.9645	**8.8688**	8.8688	8.2157	**8.2157**
	20%	9.0341	8.6471	8.7032	8.6803	8.7323	**8.6876**	**8.6877**	**7.8075**	**7.8075**
	30%	9.0503	**8.2133**	8.3191	8.2783	8.3783	8.5603	8.5604	7.3119	**7.3118**
	40%	8.9817	**7.4790**	7.6387	7.5800	7.7355	8.3201	8.3202	6.6286	**6.6285**
80	5%	9.0018	8.9773	8.9806	8.9792	8.9822	**8.9112**	8.9112	8.6939	**8.6938**
	10%	8.9581	8.8645	8.8788	8.8728	8.8858	**8.7871**	8.7871	8.3708	**8.3706**
	15%	9.0817	8.8641	8.8973	8.8837	8.9142	**8.8217**	8.8218	**8.1475**	**8.1477**
	20%	8.8724	8.4981	8.5522	8.5304	8.5805	**8.5431**	**8.5432**	7.7013	**7.7012**
	30%	9.0138	**8.1621**	8.2701	8.2281	8.3304	8.5209	8.5210	7.2562	**7.2561**
	40%	9.0067	**7.4864**	7.6472	7.5881	7.7448	8.3411	8.3412	6.6400	**6.6399**
100	5%	8.9524	8.9275	8.9307	8.9293	8.9322	**8.8680**	8.8681	8.6711	**8.6709**
	10%	8.9776	8.8862	8.9007	8.8947	8.9079	**8.8116**	8.8117	**8.3873**	**8.3874**
	15%	8.9764	8.7649	8.7972	8.7839	8.8136	**8.7274**	8.7275	**8.0858**	**8.0859**
	20%	9.0657	8.6792	8.7349	8.7124	8.7641	**8.7292**	8.7293	**7.8648**	**7.8649**
	30%	8.9626	**8.0959**	8.2053	8.1630	8.2666	8.4638	8.4639	**7.1761**	**7.1762**
	40%	9.0209	**7.4968**	7.6576	7.5987	7.7554	8.3561	8.3563	6.6504	**6.6504**
120	5%	9.0431	9.0188	9.0224	9.0209	9.0242	**8.9543**	8.9544	**8.7297**	**8.7298**
	10%	9.0522	8.9576	8.9726	8.9663	8.9800	**8.8834**	8.8834	**8.4518**	**8.4518**
	15%	8.9858	8.7703	8.8032	8.7896	8.8198	**8.7348**	8.7349	**8.0891**	**8.0892**
	20%	8.9380	8.5485	8.6043	8.5819	8.6337	**8.6011**	**8.6012**	**7.7381**	**7.7382**
	30%	8.9930	**8.1324**	8.2407	8.1988	8.3015	8.4994	8.4995	7.2338	**7.2337**
	40%	8.9832	**7.4734**	7.6318	7.5742	7.7286	8.3263	8.3265	6.6411	**6.6410**
200	5%	9.0187	8.9942	8.9980	8.9964	8.9998	8.9340	8.9341	8.7223	**8.7222**
	10%	9.0430	8.9460	8.9614	8.9550	8.9690	8.8749	8.8750	8.4466	**8.4465**
	15%	9.0068	8.7894	8.8224	8.8089	8.8393	8.7582	8.7584	8.1202	**8.1202**
	20%	9.0213	8.6313	8.6874	8.6648	8.7168	8.6890	8.6892	**7.8329**	**7.8330**
	30%	9.0458	**8.1704**	8.2801	8.2379	8.3419	8.5477	8.5479	**7.2603**	**7.2604**
	40%	8.9758	**7.4477**	7.6077	7.5494	7.7054	8.3143	8.3146	**6.6105**	**6.6105**
500	5%	9.0099	8.9859	8.9898	8.9882	8.9918	8.9279	8.9280	8.7172	**8.7172**
	10%	8.9914	8.8935	8.9092	8.9027	8.9170	8.8262	8.8264	**8.3968**	**8.3968**
	15%	8.9688	8.7504	8.7835	8.7700	8.8004	8.7244	8.7247	**8.0905**	**8.0905**
	20%	8.9864	8.5912	8.6479	8.6251	8.6777	8.6548	8.6553	**7.7911**	**7.7912**
	30%	9.0008	**8.1260**	8.2353	8.1932	8.2969	8.5079	8.5085	**7.2293**	**7.2294**
	40%	9.0098	**7.4692**	7.6297	7.5714	7.7280	8.3471	8.3478	**6.6334**	**6.6334**

**Table 17:** Mean absolute percentage errors for the scenario where error variance was 1; sample sizes of 20, 40, 60, 80, 100, 120, 200, and 500; and missing value percentages of 5, 10, 15, 20, 30, and 40% with RSS in missing value estimation methods.

Sample	Missing	Listwise	Missing value estimation methods							
sizes	values	delition	R-RQ1	R-RQ3	AR- CRQ1	AR-CRQ3	AR-MRQ1	AR-MRQ3	AR-MCRQ1	AR-MCRQ3
20	5%	95.3747	94.7604	94.7627	94.7618	94.7638	**94.4482**	94.4482	94.7862	**94.7666**
	10%	99.6573	96.8960	96.9349	96.9184	96.9537	**96.5133**	96.5133	**97.4364**	**97.3920**
	15%	**77.0905**	77.0238	77.1048	77.0702	77.1448	**76.7960**	76.7961	**79.1349**	**79.0612**
	20%	75.8631	**75.0395**	75.2156	75.1380	75.3015	**75.0672**	**75.0673**	**76.8335**	**76.7519**
	30%	80.7803	**78.2789**	78.7097	78.5346	78.9515	79.6078	79.6079	79.0293	**78.9406**
	40%	110.4068	**106.7198**	107.1859	107.0251	107.5248	109.8223	109.8224	**109.9464**	**109.8046**
40	5%	111.2744	110.2455	110.2557	110.2512	110.2605	**109.9917**	**109.9917**	**110.4391**	**110.4185**
	10%	86.3833	85.8688	85.9092	85.8923	85.9293	**85.6247**	85.6247	87.2885	**87.2359**
	15%	92.5528	93.6412	93.8916	93.7866	94.0159	**93.3115**	93.3117	**88.8422**	**88.8538**
	20%	73.2236	**71.7732**	71.9195	71.8592	71.9991	**71.8785**	**71.8786**	**74.4370**	**74.3458**
	30%	93.7663	**85.9955**	87.0085	86.5975	87.5523	89.2722	89.2727	**77.9898**	**78.0354**
	40%	84.6630	**78.4458**	78.9665	78.7490	79.3038	81.7218	81.7221	81.5996	**81.4704**
60	5%	86.5444	85.8510	85.8616	85.8571	85.8667	**85.6399**	85.6400	**86.3362**	**86.3120**
	10%	88.3480	89.5389	89.5872	89.5670	89.6111	**89.2925**	89.2925	**90.5641**	**90.5172**
	15%	110.6973	112.6806	113.3106	113.0526	113.6269	**112.0611**	112.0619	**92.7444**	**92.9263**
	20%	86.0313	**85.4402**	85.6186	85.5447	85.7139	**85.5969**	**85.5971**	**87.5550**	**87.4670**
	30%	72.7656	**68.9958**	69.2683	69.1587	69.4367	70.0357	70.0359	73.0802	**72.9461**
	40%	79.1220	**73.6668**	74.0693	73.8928	74.3378	76.4506	76.4510	77.9946	**77.8465**
80	5%	226.0563	222.6887	222.7046	222.6980	222.7123	**222.4059**	**222.4059**	222.4830	**222.4655**
	10%	169.0548	171.0901	171.1397	171.1187	171.1643	**170.8545**	170.8547	172.1611	**172.1130**
	15%	76.3482	74.9303	75.0295	74.9885	75.0809	**74.8255**	74.8257	**76.8243**	**76.7502**
	20%	81.0644	**78.4086**	78.7821	78.6291	78.9763	**78.7734**	**78.7739**	74.8995	**74.8836**
	30%	71.2061	**68.1303**	68.4284	68.3086	68.6115	69.2638	69.2642	71.7409	**71.6106**
	40%	97.9712	**89.8632**	90.6752	90.3651	91.1942	94.6932	94.6938	89.2844	**89.2156**
100	5%	108.4177	107.9050	107.9182	107.9127	107.9246	**107.6892**	107.6893	108.2595	**108.2365**
	10%	69.5005	68.9233	68.9777	68.9548	69.0047	**68.6761**	68.6762	**69.6230**	**69.5800**
	15%	74.2229	72.8707	72.9958	72.9431	73.0593	**72.7547**	72.7550	**73.8859**	**73.8233**
	20%	104.0180	**102.9827**	103.1763	103.0983	103.2813	**103.1754**	103.1758	**104.8035**	**104.7176**
	30%	137.3405	**134.3159**	134.7270	134.5703	134.9775	135.8635	135.8641	**135.9066**	**135.8021**
	40%	75.6245	**70.7699**	71.0220	70.9180	71.2294	73.0642	73.0647	76.2107	**76.0358**
120	5%	117.4171	110.8555	111.0346	110.9610	111.1227	**107.9014**	107.9031	**87.4125**	**87.6371**
	10%	75.8867	75.2628	75.3161	75.2939	75.3428	**75.0275**	75.0277	**76.0948**	**76.0498**
	15%	89.8514	86.4218	86.5842	86.5176	86.6674	**86.2737**	86.2741	**85.7168**	**85.6747**
	20%	74.7574	**73.1423**	73.3105	73.2414	73.4024	**73.3142**	**73.3146**	**75.7144**	**75.6202**
	30%	79.5506	**75.8699**	76.2900	76.1173	76.5315	77.3816	77.3822	77.6837	**77.5819**
	40%	77.2808	**71.7693**	72.0881	71.9619	72.3365	74.4156	74.4162	76.5590	**76.3962**
200	5%	80.3350	80.1389	80.1524	80.1467	80.1589	**79.9440**	79.9442	80.6090	**80.5847**
	10%	91.9970	91.6636	91.7127	91.6922	91.7373	**91.4564**	91.4567	92.8283	**92.7799**
	15%	**95.0977**	95.5675	95.7074	95.6501	95.7799	95.4504	95.4510	95.9424	**95.8873**
	20%	82.1227	80.6674	**80.8233**	80.7594	80.9089	80.8356	80.8361	**83.5682**	**83.4690**
	30%	141.3563	**138.8085**	139.0878	138.9755	139.2628	139.9241	139.9249	**142.8774**	**142.7394**
	40%	79.7663	**74.5680**	74.8671	74.7435	75.1004	77.1151	77.1161	**79.6292**	**79.4607**
500	5%	90.2270	89.7014	89.7164	89.7101	89.7236	**89.4990**	89.4994	90.0571	**90.0338**
	10%	77.4495	76.9062	76.9657	76.9409	76.9956	**76.6642**	76.6651	**77.4929**	**77.4490**
	15%	120.0434	119.3034	119.4234	119.3741	119.4859	**119.2154**	119.2166	**120.5326**	**120.4656**
	20%	77.2466	**75.6143**	75.8153	75.7340	75.9250	75.8454	75.8471	**77.2497**	**77.1649**
	30%	149.4028	**146.9771**	147.2354	147.1310	147.3982	148.0304	148.0323	**151.3856**	**151.2424**
	40%	115.3632	**105.2425**	106.1978	105.8445	106.8095	110.9156	110.9203	**103.0001**	**102.9567**

**Table 18:** Mean absolute percentage errors for the scenario where error variance was 3; sample sizes of 20, 40, 60, 80, 100, 120, 200, and 500; and missing value percentages of 5, 10, 15, 20, 30, and 40% with RSS in missing value estimation methods.

Sample	Missing	Listwise	Missing value estimation methods							
sizes	values	delition	R-RQ1	R-RQ3	AR- CRQ1	AR-CRQ3	AR-MRQ1	AR-MRQ3	AR-MCRQ1	AR-MCRQ3
20	5%	148.7004	147.4176	147.4267	147.4225	147.4307	**146.6588**	146.6589	**144.1128**	**144.12**
	10%	148.2063	146.6777	146.7414	146.7146	146.7722	**146.0009**	146.001	**143.7579**	**143.7333**
	15%	**131.9026**	129.9733	130.1293	130.063	130.2052	**129.5127**	129.5128	**126.9238**	**126.8864**
	20%	123.6774	**120.8053**	121.2421	121.0556	121.4557	**120.882**	**120.8822**	**113.0267**	**113.0287**
	30%	139.765	**132.5015**	133.4867	133.1049	134.0208	135.3684	135.3686	**122.4487**	**122.4526**
	40%	224.5832	**210.2758**	211.6033	211.1259	212.3962	216.7174	216.7176	**203.8819**	**203.8312**
40	5%	216.3412	215.8688	215.8878	215.8797	215.8968	**215.3702**	**215.3703**	**213.8529**	**213.8445**
	10%	143.3276	142.7209	142.8026	142.7686	142.8429	**142.1898**	142.1899	140.0794	**140.0546**
	15%	149.2463	145.4361	145.8217	145.6613	146.0123	**144.883**	144.8833	**133.8658**	**133.9255**
	20%	134.3183	**131.7462**	132.1147	131.9636	132.3024	**131.9882**	**131.9885**	**127.0279**	**126.9879**
	30%	150.9869	**138.8453**	140.5751	139.8736	141.4759	144.1968	144.1975	**118.7213**	**118.8908**
	40%	170.0963	**156.3779**	158.0601	157.4238	159.0274	164.5536	164.5541	**146.9916**	**147.0083**
60	5%	188.0755	187.9218	187.9416	187.9332	187.9511	**187.5072**	187.5073	**186.3435**	**186.3325**
	10%	216.0167	213.7736	213.8692	213.8296	213.9163	**213.2418**	213.2421	**210.671**	**210.6512**
	15%	178.455	178.1401	178.9928	178.6437	179.4192	**177.2653**	177.2665	**147.9714**	**148.248**
	20%	149.6323	**146.4814**	146.9416	146.7571	147.1795	**146.88**	**146.8804**	**139.3871**	**139.3746**
	30%	166.8697	**161.9126**	162.7194	162.4096	163.168	164.568	164.5685	156.3299	**156.2768**
	40%	163.3673	**143.6991**	146.3582	145.2955	147.8135	156.0269	156.028	**125.2971**	**125.4925**
80	5%	192.3991	189.5935	189.6167	189.607	189.628	**189.1522**	**189.1523**	187.7387	**187.7305**
	10%	148.9303	150.4853	150.5843	150.5433	150.6332	**149.9741**	149.9744	147.4152	**147.3932**
	15%	130.0289	128.7169	128.9364	128.8461	129.0468	**128.4726**	128.473	**124.7379**	**124.7095**
	20%	135.0307	**132.0583**	132.7596	132.4725	133.1151	**132.7214**	**132.7224**	**118.4923**	**118.5653**
	30%	118.0557	**112.0078**	112.8233	112.5106	113.2766	114.7215	114.7222	106.4259	**106.3691**
	40%	172.0073	**154.5334**	156.7095	155.9134	157.9804	165.2652	165.2664	**139.9372**	**140.0389**
100	5%	153.285	153.356	153.3806	153.3702	153.3924	**152.9225**	152.9227	151.5672	**151.5574**
	10%	121.301	120.5148	120.6154	120.5735	120.6648	**120.0171**	120.0175	**117.4126**	**117.393**
	15%	125.0877	122.8623	123.1318	123.0193	123.2651	**122.5969**	122.5976	**117.1411**	**117.1325**
	20%	184.055	**179.833**	180.2444	180.0813	180.4598	**180.2335**	180.2342	**174.2523**	**174.222**
	30%	271.7702	**265.8026**	266.6883	266.349	267.1789	268.7607	268.7616	**259.0056**	**258.9684**
	40%	130.6342	**117.6341**	119.1487	118.6001	120.0562	125.4038	125.4049	110.0513	**110.0294**
120	5%	157.4864	151.45	151.5896	151.5322	151.6581	**149.1282**	149.1295	**133.1545**	**133.3172**
	10%	130.2704	129.4296	129.5362	129.4919	129.5888	**128.9172**	128.9177	**126.0356**	**126.0213**
	15%	165.86	160.5148	160.9261	160.7637	161.1384	**160.1022**	160.1033	**148.4212**	**148.4798**
	20%	144.6316	**142.3017**	142.7067	142.543	142.9166	**142.7079**	**142.7087**	**137.0833**	**137.0507**
	30%	135.7536	**129.3199**	130.1618	129.8328	130.6232	132.1191	132.1202	123.6873	**123.6338**
	40%	127.3288	**114.9126**	116.331	115.8186	117.1866	122.2779	122.2792	108.2373	**108.1999**
200	5%	144.883	144.4322	144.4575	144.4468	144.4696	**144.0323**	144.0327	142.8001	**142.7888**
	10%	148.8196	148.0708	148.1723	148.1302	148.2225	**147.6081**	147.6088	145.1979	**145.1765**
	15%	**173.2969**	170.5753	171.1511	170.9203	171.4447	170.0699	170.0725	**152.2027**	**152.3367**
	20%	140.0196	137.4428	**137.8296**	137.6736	138.0303	137.8534	137.8547	**132.9558**	**132.9127**
	30%	189.0135	**183.2443**	184.0676	183.753	184.5276	186.0512	186.053	**177.6797**	**177.6249**
	40%	145.2864	**133.1912**	134.5667	134.0675	135.3964	140.3636	140.3658	**127.1514**	**127.103**
500	5%	165.2871	164.8702	164.8965	164.8854	164.9093	**164.4794**	164.4803	163.26	**163.2485**
	10%	153.5105	152.5538	152.6609	152.6166	152.7139	**152.0908**	152.0926	**149.4424**	**149.4222**
	15%	290.5566	287.9215	288.1717	288.0699	288.2987	**287.73**	287.7328	**283.1057**	**283.084**
	20%	140.6797	**137.9772**	138.4022	138.2325	138.624	138.4612	138.4646	**132.4623**	**132.4306**
	30%	143.7811	**138.2499**	139.0236	138.727	139.4562	140.9199	140.924	**133.6638**	**133.5954**
	40%	160.6603	**147.5575**	149.0514	148.5117	149.9505	155.3527	155.3585	**140.2399**	**140.2145**

As shown in Table 12, for an error variance of 1, for every sample size and missing value percentage, the AR-MRQ1 method achieved the minimum mean square error, followed by AR-MRQ3. On the other hand, as shown in Tables 13, 14, and 15, for error variances of 3, 5, and 7, every sample size and missing value percentage, the AR-MCRQ3 method achieved the minimum mean square error, followed by AR-MCRQ1. As shown in Table 16, for an error variance of 9 and most of the sample sizes and missing value percentage, the AR-MCRQ1 method achieved the minimum mean square error, followed by AR-MCRQ3.

Mean absolute percentage error for various simulation scenarios are the following: error variances of 1, 3, 5, 7, and 9; sample sizes of 20, 40, 60, 80, 100, 120, 200, and 500; and missing value percentages of 5, 10, 15, 20, 30, and 40% with RSS in missing value estimation methods as shown in Tables 17, 18, 19, 20, and 21.

As shown in Table 17, for error variance of 1, for most of the sample size and missing value percentages, the AR-MRQ1 method achieved the minimum mean square error, followed by AR-MRQ3. On the other hand, as shown in Tables 19, 20, and 21, for error variances of 5, 7, and 9, most of the sample size and missing value percentage, the AR-MCRQ1 method achieved the minimum mean absolute percentage error, followed by AR-MCRQ3. As shown in Table 18, for an error variance of 3 and most of the sample sizes and missing value percentage, the AR-MCRQ3 method achieved the minimum mean square error, followed by AR-MCRQ1.

**Table 19:** Mean absolute percentage errors for the scenario where error variance was 5; sample sizes of 20, 40, 60, 80, 100, 120, 200, and 500; and missing value percentages of 5, 10, 15, 20, 30, and 40% with RSS in missing value estimation methods.

Sample	Missing	Listwise	Missing value estimation methods							
sizes	values	delition	R-RQ1	R-RQ3	AR- CRQ1	AR-CRQ3	AR-MRQ1	AR-MRQ3	AR-MCRQ1	AR-MCRQ3
20	5%	203.4985	203.6339	203.6412	203.6383	203.6448	**202.8757**	202.8757	**200.0515**	**200.0527**
	10%	232.8664	233.0204	233.1128	233.0747	233.1582	**232.0337**	232.0338	**226.3982**	**226.4118**
	15%	**172.9467**	171.1848	171.394	171.3073	171.4973	**170.5421**	170.5423	**164.5503**	**164.5355**
	20%	161.2985	**159.1848**	159.7018	159.4849	159.9567	**159.244**	**159.2442**	**147.859**	**147.8945**
	30%	181.5617	**172.2612**	173.3872	172.9302	173.9723	175.3947	175.395	**159.5117**	**159.5387**
	40%	418.1684	**407.0691**	408.9114	408.2453	409.9874	415.691	415.6912	**393.9917**	**394.0337**
40	5%	250.7755	251.1941	251.2146	251.2058	251.2243	**250.6466**	**250.6467**	**248.5604**	**248.5564**
	10%	165.4405	163.9509	164.0606	164.0146	164.1139	**163.2386**	163.2388	158.7511	**158.7482**
	15%	177.7255	178.142	178.5985	178.4099	178.8251	**177.4586**	177.4591	**162.6021**	**162.6984**
	20%	170.865	**167.9483**	168.4887	168.2686	168.7632	**168.3049**	**168.3053**	**157.2995**	**157.3227**
	30%	176.3063	**166.4843**	168.2611	167.5666	169.2101	172.026	172.0267	**143.5701**	**143.749**
	40%	193.1219	**175.6379**	177.8022	176.9891	179.0332	185.8455	185.8461	**160.4514**	**160.5423**
60	5%	192.0564	191.1502	191.1809	191.1681	191.1958	**190.5018**	190.502	**187.5034**	**187.5107**
	10%	215.7246	213.4402	213.5543	213.5066	213.6099	**212.7942**	212.7945	**208.6318**	**208.6247**
	15%	232.7915	230.6326	231.8077	231.3271	232.3945	**229.4203**	229.4219	**186.4093**	**186.8393**
	20%	170.5855	**166.4239**	167.0154	166.7763	167.3177	**166.9298**	**166.9304**	**154.6705**	**154.709**
	30%	182.8097	**176.044**	177.1218	176.7111	177.7131	179.5262	179.5269	**164.9662**	**164.975**
	40%	177.3301	**158.4101**	160.9448	159.9731	162.3604	170.2739	170.275	**139.9159**	**140.0728**
80	5%	463.1782	466.5835	466.6235	466.6069	466.643	**465.8154**	**465.8156**	**461.801**	**461.8195**
	10%	292.6208	293.7304	293.8661	293.8094	293.9324	**293.028**	293.0284	**287.8018**	**287.807**
	15%	170.6454	168.5636	168.8625	168.7398	169.0122	**168.2237**	168.2243	**160.6225**	**160.6296**
	20%	175.6086	**168.6279**	169.5391	169.1633	169.9959	**169.4835**	**169.4847**	**148.2162**	**148.3669**
	30%	165.8679	**156.9776**	158.1874	157.7253	158.8495	160.9189	160.9199	**143.9081**	**143.9383**
	40%	201.0647	**177.4169**	180.4935	179.3782	182.2689	192.1717	192.1734	**152.2446**	**152.4981**
100	5%	250.8865	250.8178	250.8485	250.8356	250.8633	**250.2654**	250.2656	**247.7555**	**247.7569**
	10%	159.4151	158.2652	158.3919	158.3391	158.454	**157.6333**	157.6338	**152.9668**	**152.968**
	15%	151.9349	150.5516	150.8445	150.7237	150.9907	**150.2453**	150.2459	**142.9845**	**142.9888**
	20%	270.4243	**268.3731**	268.9347	268.7133	269.228	**268.9192**	268.9202	**257.5925**	**257.613**
	30%	243.1709	**232.1772**	233.6273	233.0676	234.4113	236.8885	236.89	**215.1595**	**215.2528**
	40%	177.694	**159.4658**	161.6839	160.8883	162.9878	170.3973	170.3989	**143.4997**	**143.5939**
120	5%	215.7559	209.1628	209.298	209.2423	209.3643	**206.9038**	206.9051	**191.2773**	**191.4333**
	10%	162.2093	161.1332	161.2809	161.2196	161.3534	**160.4132**	160.4139	**154.6231**	**154.6364**
	15%	229.0953	226.5701	227.3629	227.0551	227.7757	**225.7649**	225.7671	**197.4147**	**197.6589**
	20%	172.8289	**169.0928**	169.6121	169.404	169.88	**169.6111**	**169.6121**	**159.864**	**159.8679**
	30%	199.4369	**190.9045**	192.0939	191.6382	192.7429	194.8145	194.8159	**178.3088**	**178.3383**
	40%	175.7893	**158.7317**	160.8544	160.09	162.0986	169.1913	169.1931	**144.061**	**144.1354**
200	5%	162.4653	162.2933	162.3253	162.3118	162.3407	**161.7804**	161.7809	159.4553	**159.4551**
	10%	184.3058	183.6324	183.7619	183.7082	183.8257	**183.0328**	183.0338	178.4344	**178.4342**
	15%	**322.1418**	318.833	319.2925	319.1073	319.5257	318.4248	318.4269	**304.6485**	**304.7275**
	20%	170.9484	167.3597	**167.922**	167.6961	168.2115	167.9577	167.9595	**157.0452**	**157.0634**
	30%	191.3155	**182.3733**	183.5299	183.0896	184.1655	186.2291	186.2314	**170.3903**	**170.4096**
	40%	180.6005	**164.487**	166.4627	165.7535	167.6262	174.3219	174.3247	**151.2512**	**151.3016**
500	5%	187.7353	187.3017	187.34	187.3239	187.3585	**186.7269**	186.7283	**183.8233**	**183.8293**
	10%	183.5493	182.3278	182.4947	182.4256	182.577	**181.5998**	181.6027	**174.9747**	**174.9977**
	15%	324.4419	321.636	321.9534	321.8245	322.114	**321.3908**	321.3943	**313.4787**	**313.4879**
	20%	196.1359	**192.4507**	193.0014	192.782	193.2871	193.0766	193.081	**182.5253**	**182.5387**
	30%	206.956	**198.5238**	199.7337	199.2726	200.3978	202.5976	202.6037	**185.8272**	**185.8588**
	40%	201.1646	**180.8262**	183.3337	182.4421	184.8089	193.2659	193.2747	**161.7957**	**161.9435**

**Table 20:** Mean absolute percentage errors for the scenario where error variance was 7; sample sizes of 20, 40, 60, 80, 100, 120, 200, and 500; and missing value percentages of 5, 10, 15, 20, 30, and 40% with RSS in missing value estimation methods.

Sample	Missing	Listwise	Missing value estimation methods							
sizes	values	delition	R-RQ1	R-RQ3	AR- CRQ1	AR-CRQ3	AR-MRQ1	AR-MRQ3	AR-MCRQ1	AR-MCRQ3
20	5%	228.2015	228.1494	228.1575	228.154	228.1614	**227.2854**	227.2855	**223.6198**	**223.636**
	10%	223.0908	223.707	223.81	223.7677	223.8607	**222.5859**	222.586	**215.6303**	**215.6536**
	15%	**234.7358**	233.1497	233.4115	233.3033	233.541	**232.3515**	232.3517	**223.2659**	**223.2915**
	20%	192.5133	**187.9682**	188.624	188.3506	188.9485	**188.0363**	**188.0366**	**171.4458**	**171.5334**
	30%	205.2531	**194.8874**	196.3403	195.7612	197.1004	198.938	198.9383	**175.3498**	**175.4657**
	40%	240.3467	**220.2805**	222.5567	221.7191	223.8538	230.5951	230.5954	**201.6883**	**201.8144**
40	5%	196.2122	195.2874	195.3122	195.3015	195.324	**194.6167**	**194.6168**	**191.6135**	**191.6195**
	10%	222.0807	221.4443	221.5652	221.5145	221.6239	**220.6377**	220.6379	**215.0701**	**215.077**
	15%	306.2351	297.7138	298.7151	298.3097	299.219	**296.2675**	296.2684	**257.6727**	**258.0466**
	20%	195.8845	**192.3611**	192.9172	192.692	193.2001	**192.7225**	**192.7228**	**180.4368**	**180.4716**
	30%	202.577	**188.2687**	190.3526	189.5568	191.4807	194.8509	194.8517	**158.7188**	**158.9845**
	40%	224.9357	**205.4141**	207.9515	207.0064	209.3896	217.2406	217.2413	**185.4819**	**185.6424**
60	5%	210.8323	209.8095	209.8415	209.8277	209.8566	**209.1425**	209.1426	**205.8679**	**205.8791**
	10%	228.619	229.5535	229.7035	229.6401	229.776	**228.7226**	228.723	**221.9457**	**221.9686**
	15%	228.9357	231.0276	231.7774	231.4731	232.1542	**230.1854**	230.1865	**203.5412**	**203.7727**
	20%	201.9847	**196.5299**	197.2961	196.9914	197.6915	**197.1897**	**197.1904**	**178.8487**	**178.9491**
	30%	239.0615	**228.0592**	229.4294	228.9078	230.1762	232.452	232.4528	**211.1345**	**211.2211**
	40%	220.0078	**194.3276**	197.6428	196.3703	199.4769	209.5758	209.5771	**167.037**	**167.3451**
80	5%	523.0761	526.462	526.5049	526.487	526.5257	**525.6366**	**525.6369**	**521.0491**	**521.0743**
	10%	333.9213	330.858	331.0087	330.9462	331.0828	**330.0621**	330.0626	**323.3813**	**323.4026**
	15%	204.2614	202.0052	202.3434	202.2045	202.5123	**201.6174**	201.618	**192.0286**	**192.0597**
	20%	188.7285	**184.3244**	185.074	184.7688	185.4542	**185.0166**	**185.0176**	**167.7243**	**167.8206**
	30%	189.5829	**179.6569**	181.045	180.5198	181.8045	184.1634	184.1645	**162.6624**	**162.7469**
	40%	203.1449	**180.5601**	183.4352	182.3991	185.101	194.3648	194.3664	**156.6919**	**156.9185**
100	5%	436.6739	433.5618	433.5977	433.5826	433.615	**432.9114**	432.9117	**429.5484**	**429.559**
	10%	194.7727	193.8534	194.0193	193.9502	194.1004	**193.0255**	193.0262	**185.6125**	**185.6444**
	15%	188.6218	186.627	187.0203	186.857	187.215	**186.2265**	186.2274	**174.6678**	**174.7232**
	20%	365.7963	**361.0408**	361.7025	361.4396	362.0451	**361.6817**	361.6828	**346.8159**	**346.8781**
	30%	228.324	**215.5626**	217.2728	216.6235	218.2045	221.144	221.1457	**193.2026**	**193.3652**
	40%	202.1106	**181.044**	183.7219	182.7645	185.2817	194.0028	194.0047	**159.3862**	**159.5721**
120	5%	234.3666	227.9744	228.1387	228.071	228.2192	**225.2223**	225.2239	**205.754**	**205.9539**
	10%	196.7276	195.72	195.8797	195.8133	195.9581	**194.9351**	194.9358	**188.1226**	**188.1463**
	15%	265.5601	257.7207	258.5367	258.2177	258.9592	**256.8947**	256.897	**227.2362**	**227.4953**
	20%	222.5236	**218.369**	219.0118	218.7553	219.3432	**219.0127**	**219.014**	**204.906**	**204.961**
	30%	206.4988	**195.8267**	197.3552	196.7709	198.1859	200.8182	200.82	**176.8137**	**176.9302**
	40%	220.9084	**198.6209**	201.4449	200.4479	203.1005	212.3688	212.3711	**175.1843**	**175.3978**
200	5%	202.7456	202.5678	202.6066	202.5903	202.6254	**201.9404**	201.941	**198.5833**	**198.5945**
	10%	233.0575	231.9901	232.1433	232.0799	232.2188	**231.2765**	231.2776	**224.9141**	**224.9333**
	15%	**345.1219**	342.4259	343.015	342.7776	343.3135	341.9037	341.9064	**322.4493**	**322.5944**
	20%	221.5589	216.8441	**217.5607**	217.273	217.9284	217.6054	217.6077	**201.6991**	**201.7768**
	30%	444.2255	**433.2253**	434.6992	434.1408	435.5053	438.1062	438.1091	**415.1635**	**415.2635**
	40%	202.0543	**183.0794**	185.4587	184.6067	186.848	194.7104	194.7137	**164.8232**	**164.9529**
500	5%	225.5124	224.9936	225.0379	225.0193	225.0593	**224.3253**	224.3269	**220.5358**	**220.5518**
	10%	206.9888	205.5938	205.7859	205.7065	205.8806	**204.7512**	204.7545	**196.3273**	**196.37**
	15%	575.1941	572.5294	572.9279	572.7663	573.1291	**572.221**	572.2255	**560.6603**	**560.7124**
	20%	219.0306	**213.8623**	214.6288	214.3254	215.0264	214.7357	214.7418	**197.1743**	**197.2675**
	30%	246.7447	**236.4599**	237.9434	237.3793	238.7536	241.4184	241.4257	**218.4774**	**218.5783**
	40%	282.9756	**255.2549**	258.767	257.5235	260.8159	272.3538	272.3656	**224.6084**	**224.9469**

**Table 21:** Mean absolute percentage errors for the scenario where error variance was 9; sample sizes of 20, 40, 60, 80, 100, 120, 200, and 500; and missing value percentages of 5, 10, 15, 20, 30, and 40% with RSS in missing value estimation methods.

Sample	Missing	Listwise	Missing value estimation methods							
sizes	values	delition	R-RQ1	R-RQ3	AR- CRQ1	AR-CRQ3	AR-MRQ1	AR-MRQ3	AR-MCRQ1	AR-MCRQ3
20	5%	308.8765	308.197	308.211	308.2049	308.2176	**307.0716**	307.0717	**301.5634**	**301.6033**
	10%	282.5199	283.1238	283.2537	283.199	283.3165	**281.7664**	281.7666	**272.3811**	**272.4397**
	15%	**263.0108**	260.6264	260.9332	260.8052	261.0838	**259.7081**	259.7083	**248.0169**	**248.0747**
	20%	232.7102	**228.1869**	228.9492	228.6252	229.3197	**228.2706**	**228.2709**	**207.8136**	**207.9582**
	30%	232.3853	**221.2005**	222.8405	222.1925	223.7028	225.7808	225.7812	**197.391**	**197.5702**
	40%	406.5708	**387.9422**	390.7279	389.7766	392.3832	400.9869	400.9873	**361.3455**	**361.5868**
40	5%	379.8662	380.452	380.4787	380.4672	380.4913	**379.7216**	**379.7217**	**376.0797**	**376.0945**
	10%	248.4955	247.1534	247.307	247.2429	247.3819	**246.1384**	246.1387	**237.9563**	**237.9977**
	15%	265.2678	257.0216	257.8201	257.4933	258.2182	**255.8598**	255.8605	**225.72**	**225.9935**
	20%	234.4496	**229.6977**	230.4186	230.1257	230.7837	**230.1827**	**230.1832**	**212.4326**	**212.5365**
	30%	267.904	**245.9249**	249.1849	247.8945	250.8937	256.0055	256.0068	**197.1349**	**197.6947**
	40%	256.4665	**230.9655**	234.3916	233.1152	236.3205	246.7354	246.7363	**200.6448**	**200.9831**
60	5%	274.0201	274.4118	274.4555	274.4367	274.4761	**273.5131**	273.5133	**268.3973**	**268.4315**
	10%	297.0196	293.776	293.9538	293.8793	294.0403	**292.779**	292.7794	**283.9151**	**283.9634**
	15%	246.4003	236.9198	237.5005	237.265	237.7926	**236.2123**	236.2132	**216.0172**	**216.1702**
	20%	230.9762	**225.1471**	225.9894	225.6529	226.4216	**225.868**	**225.8689**	**204.7622**	**204.9003**
	30%	231.9347	**222.197**	223.707	223.1347	224.53	227.0241	227.025	**202.3304**	**202.4565**
	40%	251.4197	**221.6351**	225.5718	224.0549	227.7331	239.533	239.5346	**187.396**	**187.8294**
80	5%	967.226	963.3419	963.3946	963.3726	963.4201	**962.3332**	**962.3335**	**956.3012**	**956.3435**
	10%	294.4182	291.3296	291.501	291.4297	291.5848	**290.4254**	290.426	**282.2979**	**282.3367**
	15%	219.777	217.1951	217.5889	217.4274	217.7856	**216.741**	216.7418	**204.398**	**204.4622**
	20%	235.5731	**228.5523**	229.7202	229.2459	230.3115	**229.654**	**229.6555**	**199.6527**	**199.9009**
	30%	214.2518	**202.8536**	204.4767	203.8616	205.3603	208.096	208.0973	**181.2805**	**181.4309**
	40%	234.6107	**211.7121**	214.5894	213.5512	216.2473	225.4171	225.4187	**187.4886**	**187.7172**
100	5%	266.2074	263.5141	263.5554	263.538	263.5753	**262.7665**	262.7669	**258.5331**	**258.5553**
	10%	222.9288	222.0292	222.2086	222.1338	222.2964	**221.1271**	221.1278	**212.6534**	**212.6984**
	15%	209.4874	207.3642	207.7896	207.6133	208.0004	**206.9251**	206.926	**193.7235**	**193.7994**
	20%	247.7401	**243.7068**	244.4218	244.1387	244.7918	**244.3955**	244.3967	**227.4555**	**227.5433**
	30%	337.7131	**325.6391**	327.4766	326.7791	328.4748	331.607	331.6088	**300.6692**	**300.8706**
	40%	247.8758	**219.6567**	223.2796	221.9976	225.3865	237.0336	237.0361	**186.9572**	**187.327**
120	5%	298.5901	302.1416	302.4685	302.3342	302.6291	**296.6876**	296.6908	**256.314**	**256.7603**
	10%	218.5231	217.5994	217.7944	217.7135	217.8902	**216.64**	216.6409	**207.2759**	**207.3301**
	15%	262.6312	255.2925	256.0624	255.7573	256.457	**254.5206**	254.5227	**226.7402**	**226.9829**
	20%	221.1238	**216.0352**	216.7419	216.4616	217.1074	**216.7425**	**216.7439**	**200.2304**	**200.3137**
	30%	228.8371	**214.5518**	216.7147	215.8572	217.8526	221.4415	221.444	**185.3284**	**185.6018**
	40%	235.3645	**209.4075**	212.7492	211.5557	214.6846	225.4279	225.4306	**180.0424**	**180.3591**
200	5%	231.7475	231.4468	231.4882	231.4707	231.508	**230.7783**	230.779	**227.0022**	**227.0184**
	10%	293.9669	292.9684	293.1504	293.075	293.2399	**292.1197**	292.121	**283.8424**	**283.8848**
	15%	**339.4221**	334.7767	335.5938	335.2666	336.009	334.0473	334.0511	**305.0469**	**305.3043**
	20%	251.2568	246.4437	**247.2226**	246.9104	247.6223	247.2704	247.2729	**228.9295**	**229.0375**
	30%	280.9567	**268.8168**	270.4407	269.8256	271.3272	274.1788	274.182	**247.7143**	**247.861**
	40%	223.1637	**200.3712**	203.2759	202.241	204.9658	214.4283	214.4322	**175.8**	**176.0366**
500	5%	262.8758	262.4849	262.5314	262.5119	262.5539	**261.7805**	261.7823	**257.6132**	**257.6336**
	10%	239.9691	238.7844	239.0186	238.9218	239.1339	**237.7572**	237.7613	**226.5883**	**226.6647**
	15%	381.6172	378.5317	379.005	378.8127	379.2434	**378.1661**	378.1714	**363.3493**	**363.4415**
	20%	263.5808	**258.5161**	259.3197	259.0003	259.7348	259.4297	259.4361	**240.3806**	**240.4947**
	30%	263.3312	**251.0471**	252.7271	252.0891	253.6423	256.6418	256.65	**229.1672**	**229.327**
	40%	334.5533	**297.1716**	302.0105	300.2928	304.8091	320.4186	320.4345	**251.6578**	**252.2517**

### 3.2. Results of a study on actual data

Mean square errors and mean absolute percentage errors of gold loss for the scenarios where error variance was 1, 3, 5, 7, and 9; a sample of 48; and missing value percentages of 5, 10, 15, 20, 30 and 40% with SRS and RSS are shown in Tables 22, 23, 24, and 25.

Table 22 shows that for all error variances and missing value percentages of gold loss, the AR-MRQ3 method achieved the minimum mean square error with SRS.

**Table 22:** Mean square errors of gold loss for error variances of 1, 3, 5, 7, and 9; a sample sizes of 48; and missing value percentages of 5, 10, 15, 20, 30, and 40% with SRS in missing value estimation methods.

Sample	Missing	Listwise	Missing value estimation methods							
sizes	values	delition	R-RQ1	R-RQ3	AR- CRQ1	AR-CRQ3	AR-MRQ1	AR-MRQ3	AR-MCRQ1	AR-MCRQ3
1	5%	110539.1	111617.2	111578.1	111609.6	111574.6	111602	**111571.1**	116864.3	115628.8
	10%	110213.2	111703.1	111554.1	111672.5	111541.4	111640.9	**111528.5**	121832.1	119349.5
	15%	110230	111887.5	111531.1	111808.7	111503.4	111726.5	**111475.4**	128070.6	123903.1
	20%	110120.6	112524.2	111776.4	112354.5	111718.9	112172.2	**111660.7**	132961.2	127571.5
	30%	109975.4	114727.7	112404.6	114183.3	112222.4	113552.9	**112033.4**	143917.3	135609
	40%	110159	119747	114128.1	118429.6	113634.4	116734.2	**113092.9**	155642.5	144289.8
3	5%	110535.6	111613.7	111574.6	111606.1	111571.1	111598.4	**111567.5**	116860.7	115625.2
	10%	110226.5	111717.9	111568.8	111687.3	111556.1	111655.7	**111543.2**	121847.6	119365
	15%	110229	111886.5	111530.1	111807.7	111502.3	111725.5	**111474.3**	128068.7	123901.6
	20%	110121.2	112523.9	111776.2	112354.2	111718.7	112171.9	**111660.5**	132960.2	127570.5
	30%	109964.6	114716.1	112393.1	114171.7	112210.9	113541.4	**112022**	143906.2	135597.6
	40%	110163.3	119749.3	114131.1	118432	113637.4	116736.8	**113096**	155644.8	144291.9
5	5%	110531.4	111608.8	111569.7	111601.2	111566.2	111593.6	**111562.7**	116856.1	115620.5
	10%	110223.1	111712	111562.9	111681.4	111550.2	111649.8	**111537.3**	121842	119359.6
	15%	110237.3	111895.8	111539.6	111817.1	111511.8	111734.9	**111483.8**	128076.4	123909.4
	20%	110116.3	112520.3	111772.5	112350.6	111715	112168.2	**111656.8**	132957.8	127568
	30%	109981.3	114731	112409.3	114186.9	112227.3	113556.9	**112038.6**	143917.6	135609.3
	40%	110177.5	119762.1	114144.4	118444.9	113650.8	116749.8	**113109.4**	155654.4	144302.5
7	5%	110545.1	111622.8	111583.7	111615.2	111580.2	111607.5	**111576.7**	116870	115634.3
	10%	110235.6	111724	111575	111693.4	111562.3	111661.8	**111549.4**	121853.3	119370.4
	15%	110224.8	111883	111526.4	111804	111498.6	111721.9	**111470.6**	128067.6	123900.2
	20%	110129.1	112531.5	111783.6	112361.8	111726.1	112179.4	**111667.9**	132968.9	127579.2
	30%	109972.3	114720.6	112397.6	114176.3	112215.4	113545.9	**112026.4**	143911.3	135602.6
	40%	110157.6	119747.2	114128.7	118429.8	113635.1	116734.6	**113093.6**	155640.4	144288.4
9	5%	110559.8	111637.8	111598.7	111630.2	111595.2	111622.6	**111591.7**	116883.8	115648.4
	10%	110229.2	111719.6	111570.7	111689	111558	111657.4	**111545.1**	121847.3	119364.9
	15%	110231.8	111887.7	111531.8	111809	111504	111726.9	**111476.1**	128069.2	123901.6
	20%	110116.2	112517.6	111769.9	112347.9	111712.5	112165.6	**111654.3**	132955.1	127565
	30%	109972.1	114726.9	112403.4	114182.5	112221.1	113552	**112032.1**	143915.3	135607.7
	40%	110160.5	119746.8	114129.1	118429.6	113635.5	116734.5	**113094.2**	155641.8	144288.8

**Table 23:** Mean absolute percentage errors of gold loss for error variances of 1, 3, 5, 7, and 9; a sample sizes of 48; and missing value percentages of 5, 10, 15, 20, 30 and 40% with SRS in missing value estimation methods.

Sample	Missing	Listwise	Missing value estimation methods							
sizes	values	delition	R-RQ1	R-RQ3	AR- CRQ1	AR-CRQ3	AR-MRQ1	AR-MRQ3	AR-MCRQ1	AR-MCRQ3
1	5%	285.1246	285.1777	285.2769	285.1965	285.2861	285.216	**285.2954**	**281.706**	281.7864
	10%	285.3053	283.9008	284.5208	284.0185	284.5801	284.1446	**284.6415**	**274.7975**	275.5009
	15%	285.7429	280.8366	282.7458	281.1991	282.9354	281.6043	**283.1359**	**263.2475**	265.1126
	20%	285.516	277.7929	281.2114	278.4422	281.5653	279.2013	**281.9482**	**255.5784**	258.3216
	30%	285.1613	266.1675	274.5077	267.75	275.4565	269.7966	**276.5385**	**234.0563**	239.1611
	40%	285.6655	248.2271	263.2748	251.0756	265.1752	255.2599	**267.4938**	**210.3753**	218.1984
3	5%	285.1099	285.1622	285.2614	285.1811	285.2706	285.2006	**285.2799**	**281.692**	281.7702
	10%	285.3448	283.9411	284.5604	284.0586	284.6196	284.1846	**284.6809**	**274.8503**	275.55
	15%	285.7356	280.8257	282.735	281.188	282.9247	281.5933	**283.1253**	**263.2339**	265.0998
	20%	285.5071	277.7861	281.2037	278.4353	281.5574	279.1942	**281.9404**	**255.5789**	258.3219
	30%	285.154	266.1587	274.4994	267.7414	275.4483	269.7878	**276.5304**	**234.0478**	239.1522
	40%	285.6614	248.2252	263.2711	251.0734	265.1712	255.2572	**267.4901**	**210.3772**	218.1984
5	5%	285.0905	285.1424	285.2415	285.1612	285.2506	285.1807	**285.26**	**281.6761**	281.7555
	10%	285.3196	283.913	284.5331	284.0306	284.5924	284.1569	**284.6538**	**274.8077**	275.5096
	15%	285.7736	280.8668	282.7767	281.2295	282.9664	281.6348	**283.1669**	**263.2731**	265.1403
	20%	285.5079	277.7852	281.2038	278.4346	281.5577	279.1935	**281.9405**	**255.5704**	258.3132
	30%	285.2048	266.2111	274.5524	267.7935	275.5013	269.8399	**276.5835**	**234.0905**	239.198
	40%	285.7268	248.2759	263.3302	251.1268	265.2314	255.3128	**267.5509**	**210.4076**	218.235
7	5%	285.0567	285.107	285.2061	285.1258	285.2153	285.1453	**285.2246**	**281.6403**	281.7193
	10%	285.3658	283.9562	284.5771	284.074	284.6364	284.2003	**284.6979**	**274.8411**	275.5445
	15%	285.739	280.8343	282.7435	281.1968	282.9332	281.6021	**283.1337**	**263.2471**	265.1097
	20%	285.5208	277.8032	281.2194	278.4524	281.573	279.2111	**281.9556**	**255.6106**	258.3523
	30%	285.2083	266.2109	274.5525	267.7932	275.5015	269.8402	**276.5836**	**234.0954**	239.2002
	40%	285.7007	248.2489	263.3029	251.0994	265.2036	255.2851	**267.5225**	**210.3813**	218.2099
9	5%	285.1167	285.1667	285.2657	285.1855	285.2749	285.2049	**285.2842**	**281.6925**	281.7723
	10%	285.3274	283.9242	284.544	284.0418	284.6033	284.1679	**284.6647**	**274.8204**	275.5217
	15%	285.7304	280.8239	282.7339	281.1863	282.9236	281.5917	**283.1241**	**263.232**	265.0998
	20%	285.4967	277.7729	281.1929	278.4226	281.5469	279.1819	**281.93**	**255.5557**	258.2999
	30%	285.1467	266.1515	274.4927	267.7347	275.4417	269.7814	**276.5239**	**234.0298**	239.1381
	40%	285.7007	248.2634	263.3119	251.1127	265.2123	255.2982	**267.5305**	**210.4157**	218.24

Table 23 shows that for all error variances and missing value percentages of gold loss, the AR-MCRQ1 method achieved the minimum mean absolute percentage error with SRS.

**Table 24:** Mean square error of gold loss for error variances of 1, 3, 5, 7, 9; a sample sizes of 48; and missing value percentages of 5, 10, 15, 20, 30, and 40% with RSS in missing value estimation methods.

Sample	Missing	Listwise	Missing value estimation methods							
sizes	values	delition	R-RQ1	R-RQ3	AR- CRQ1	AR-CRQ3	AR-MRQ1	AR-MRQ3	AR-MCRQ1	AR-MCRQ3
1	5%	8132.28	**6509.063**	6547.956	6516.225	6551.708	6523.778	**6555.552**	**10515.27**	8676.545
	10%	11209.49	**9193.611**	9355.857	9220.557	9373.608	9250.729	**9392.239**	**21081.36**	17194.74
	15%	13114.38	11824.79	11812.56	11804.77	11823.21	**11791.35**	**11836.47**	**34381.03**	27846.01
	20%	13818.25	13271.89	12795.94	13137.57	12778.42	13006.91	**12765.64**	**42837.51**	34457.66
	30%	14427.01	18398.93	15338.08	17662.06	15116.68	16828.53	**14894.06**	**62723.06**	49943.09
	40%	14822.68	26974.59	18967.22	25087.09	18281.32	22688.67	**17538.7**	**80708.14**	63702.5
3	5%	8132.056	**6509.366**	6548.306	6516.538	6552.063	6524.099	**6555.912**	**10514.69**	8675.995
	10%	11210.62	**9196.7**	9358.917	9223.64	9376.665	9253.807	**9395.293**	**21084.63**	17198.19
	15%	13116.64	11824.58	11812.6	11804.6	11823.28	**11791.23**	**11836.57**	**34380.82**	27845.39
	20%	13818.3	13274.73	12798.71	13140.41	12781.18	13009.73	**12768.38**	**42839.67**	34460.12
	30%	14428.71	18400.03	15339.22	17663.17	15117.82	16829.65	**14895.21**	**62723.7**	49943.86
	40%	14824.26	26974.46	18967.38	25087.01	18281.53	22688.66	**17538.97**	**80708.25**	63702.38
5	5%	8133.49	**6511.043**	6549.97	6518.212	6553.725	6525.771	**6557.573**	**10518.57**	8679.683
	10%	11218	**9199.959**	9362.35	9226.931	9380.115	9257.133	**9398.761**	**21086.45**	17199.65
	15%	13118.3	11827.29	11815.36	11807.33	11826.05	**11793.97**	**11839.34**	**34382.94**	27847.65
	20%	13823.85	13277.13	12801.48	13142.87	12784	13012.28	**12771.25**	**42840.99**	34461.35
	30%	14425.81	18398.11	15337.19	17661.23	15115.78	16827.68	**14893.16**	**62722.83**	49942.7
	40%	14826.93	26976.23	18969.36	25088.82	18283.53	22690.52	**17541**	**80709.47**	63703.72
7	5%	8135.977	**6513.756**	6552.663	6520.921	6556.417	6528.477	**6560.263**	**10520.57**	8681.81
	10%	11217.2	**9204.012**	9366.461	9230.996	9384.232	9261.209	**9402.883**	**21089.44**	17202.98
	15%	13120.25	11830.71	11818.57	11810.71	11829.23	**11797.31**	**11842.5**	**34386.7**	27851.76
	20%	13826.98	13280.2	12803.98	13145.83	12786.43	13015.12	**12773.61**	**42845.55**	34465.99
	30%	14432.89	18402.81	15342.5	17666.04	15121.16	16832.63	**14898.62**	**62725.77**	49945.84
	40%	14820.91	26973.43	18966.22	25085.96	18280.36	22687.58	**17537.77**	**80706.98**	63701.26
9	5%	8140.484	**6514.446**	6553.318	6521.604	6557.069	6529.153	**6560.911**	**10521.31**	8682.343
	10%	11214.84	**9203.122**	9365.429	9230.08	9383.186	9260.264	**9401.822**	**21088.46**	17202.15
	15%	13121.63	11831.1	11819.09	11811.12	11829.77	**11797.74**	**11843.05**	**34385.98**	27851.02
	20%	13828.86	13280.39	12805.35	13146.24	12787.94	13015.77	**12775.26**	**42842.92**	34462.94
	30%	14438.27	18407.43	15346.9	17670.62	15125.53	16837.16	**14902.96**	**62730.56**	49950.7
	40%	14824.25	26976.96	18969.71	25089.51	18283.81	22691.13	**17541.18**	**80708.5**	63703.58

Table 24 shows that for all error variances and lower missing value percentages of gold loss with RSS, the AR-RQ1 method achieved the minimum mean square error. For all error variances and higher missing value percentages, the AR-MRQ1 and AR-MRQ3 methods achieved the same minimum mean square error.

**Table 25:** Mean absolute percentage error of gold loss for error variances of 1, 3, 5, 7, 9; a sample sizes of 48; and missing value percentages of 5, 10, 15, 20, 30 and 40% with RSS in missing value estimation methods.

Sample	Missing	Listwise	Missing value estimation methods							
sizes	values	delition	R-RQ1	R-RQ3	AR- CRQ1	AR-CRQ3	AR-MRQ1	AR-MRQ3	AR-MCRQ1	AR-MCRQ3
1	5%	34.4174	**36.6773**	36.9467	36.7283	36.9717	36.7812	**36.9972**	**27.0932**	28.2219
	10%	43.2773	**46.0735**	46.8215	46.2151	46.8934	46.3676	**46.9678**	**35.9682**	37.1316
	15%	46.8906	52.5821	53.9486	52.8364	54.0864	**53.1254**	**54.2324**	**43.7127**	44.6636
	20%	48.3325	52.6713	54.4667	52.9971	54.6650	53.3872	**54.8810**	**46.6301**	47.1979
	30%	49.2486	54.8575	56.8205	55.1613	57.0924	55.6140	**57.4147**	**54.4431**	**54.1486**
	40%	49.9838	55.2443	55.8013	**55.1992**	56.0337	55.2763	**56.3694**	**59.9973**	58.5468
3	5%	34.4396	**36.7275**	36.9969	36.7785	37.0219	36.8315	**37.0474**	**27.1402**	28.2693
	10%	43.3262	**46.1239**	46.8722	46.2656	46.9440	46.4181	**47.0185**	**36.0163**	37.1802
	15%	46.9147	52.6462	54.0133	52.9007	54.1511	**53.1898**	**54.2972**	**43.7745**	44.7256
	20%	48.3648	52.7304	54.5269	53.0563	54.7252	53.4469	**54.9411**	**46.6839**	47.2534
	30%	49.2656	54.9136	56.8760	55.2171	57.1482	55.6694	**57.4709**	**54.5012**	**54.2067**
	40%	49.9831	55.2646	55.8177	**55.2180**	56.0491	55.2936	**56.3830**	**60.0345**	58.5788
5	5%	34.4258	**36.7361**	37.0056	36.7872	37.0306	36.8401	**37.0561**	**27.1479**	28.2776
	10%	43.3273	**46.1545**	46.9026	46.2962	46.9745	46.4487	**47.0490**	**36.0412**	37.2056
	15%	46.8975	52.6742	54.0412	52.9289	54.1790	**53.2179**	**54.3249**	**43.8047**	44.7545
	20%	48.3321	52.7786	54.5749	53.1045	54.7731	53.4947	**54.9891**	**46.7334**	47.3020
	30%	49.2513	54.9284	56.8892	55.2315	57.1613	55.6841	**57.4829**	**54.5209**	**54.2250**
	40%	50.0131	55.3143	55.8697	**55.2687**	56.1007	55.3460	**56.4358**	**60.0728**	58.6211
7	5%	34.4869	**36.8254**	37.0948	36.8764	37.1198	36.9294	**37.1452**	**27.2414**	28.3703
	10%	43.3570	**46.2198**	46.9684	46.3615	47.0403	46.5141	**47.1148**	**36.1018**	37.2668
	15%	46.9252	52.7117	54.0794	52.9665	54.2175	**53.2554**	**54.3636**	**43.8272**	44.7811
	20%	48.3800	52.8015	54.5950	53.1271	54.7929	53.5170	**55.0085**	**46.7813**	47.3469
	30%	49.2409	54.9379	56.8992	55.2412	57.1711	55.6933	**57.4936**	**54.5257**	**54.2308**
	40%	49.9735	55.3449	55.9017	**55.2993**	56.1331	55.3766	**56.4695**	**60.1048**	58.6520
9	5%	34.4467	**36.8363**	37.1057	36.8873	37.1307	36.9402	**37.1562**	**27.2566**	28.3848
	10%	43.3663	**46.2847**	47.0328	46.4264	47.1046	46.5788	**47.1790**	**36.1767**	37.3405
	15%	46.9642	52.7755	54.1409	53.0297	54.2788	**53.3184**	**54.4249**	**43.9039**	44.8543
	20%	48.3892	52.8512	54.6454	53.1767	54.8437	53.5665	**55.0596**	**46.8185**	47.3855
	30%	49.3259	54.9726	56.9331	55.2755	57.2049	55.7263	**57.5270**	**54.5818**	**54.2828**
	40%	50.0526	55.3765	55.9356	**55.3315**	56.1676	55.4091	**56.5038**	**60.1177**	58.6709

Table 25 shows that for all error variances and missing value percentages of gold loss with RSS, the AR-MCRQ1 method achieved the minimum mean absolute percentage error. The AR-CRQ1 and AR-MCRQ3 methods achieved the minimum mean absolute percentage error for all error variances and higher missing value percentages.

Mean square errors and mean absolute percentage errors of platinum loss for the scenarios where error variances were 1, 3, 5, 7, and 9; a sample size of 12; and missing value percentages of 5, 10, 15, and 20% with SRS and RSS are shown in Tables 26, 27, 28, and 29.

**Table 26:** Mean square errors of platinum loss for the scenarios with error variance of 1, 3, 5, 7, and 9; a sample sizes of 48; and missing value percentages of 5, 10, 15, 20, 30, and 40% with SRS in missing value estimation methods.

Sample	Missing	Listwise	Missing value estimation methods							
sizes	values	delition	R-RQ1	R-RQ3	AR- CRQ1	AR-CRQ3	AR-MRQ1	AR-MRQ3	AR-MCRQ1	AR-MCRQ3
1	5%	7670.53	**7569.60**	7562.75	7568.27	7562.46	7566.97	**7562.19**	**9054.11**	8396.74
	10%	7669.48	**7578.04**	7538.82	7569.90	7537.40	7561.80	**7536.05**	**10455.59**	9161.83
	15%	7669.62	7681.90	7540.65	7652.41	7535.47	**7621.87**	**7530.51**	**12501.24**	10294.60
	20%	7667.36	7867.43	7559.70	7803.34	7547.94	7733.80	**7536.54**	**13833.33**	11025.02
	30%	7666.01	8759.05	7711.70	8546.41	7664.95	8289.20	**7617.40**	**17263.53**	**12926.55**
	40%	7670.16	10612.20	8157.01	**10125.40**	8027.39	9459.66	**7887.72**	**20870.95**	14918.74
3	5%	7673.16	**7572.00**	7565.17	7570.67	7564.88	7569.38	**7564.61**	**9056.19**	8398.88
	10%	7669.61	**7578.28**	7538.85	7570.11	7537.42	7561.98	**7536.06**	**10456.04**	9162.64
	15%	7672.13	7684.51	7543.23	7655.01	7538.05	**7624.46**	**7533.09**	**12503.74**	10297.16
	20%	7670.08	7870.63	7562.84	7806.53	7551.07	7736.98	**7539.66**	**13836.23**	11028.13
	30%	7669.02	8762.02	7714.56	8549.37	7667.80	8292.14	**7620.24**	**17266.53**	**12929.59**
	40%	7673.71	10614.88	8159.92	**10128.11**	8030.31	9462.42	**7890.68**	**20873.34**	14921.25
5	5%	7674.97	**7574.03**	7567.15	7572.70	7566.87	7571.40	**7566.59**	**9058.39**	8401.17
	10%	7671.16	**7579.72**	7540.42	7571.56	7538.99	7563.46	**7537.64**	**10457.51**	9163.75
	15%	7673.01	7685.79	7544.40	7656.28	7539.20	**7625.71**	**7534.23**	**12505.55**	10298.90
	20%	7671.81	7872.26	7564.52	7808.17	7552.76	7738.62	**7541.35**	**13838.01**	11029.75
	30%	7669.11	8760.65	7713.94	8548.10	7667.24	8291.00	**7619.76**	**17264.77**	**12927.62**
	40%	7672.27	10614.80	8159.43	**10127.96**	8029.79	9462.17	**7890.12**	**20874.47**	14921.71
7	5%	7676.67	**7576.30**	7569.45	7574.96	7569.16	7573.67	**7568.89**	**9060.22**	8402.99
	10%	7672.48	**7580.58**	7541.43	7572.44	7540.02	7564.36	**7538.68**	**10458.07**	9164.10
	15%	7673.02	7685.82	7544.48	7656.32	7539.29	**7625.76**	**7534.32**	**12504.37**	10298.20
	20%	7670.95	7871.37	7563.53	7807.27	7551.76	7737.71	**7540.34**	**13836.47**	11028.67
	30%	7672.18	8764.70	7717.71	8552.11	7670.98	8294.96	**7623.48**	**17268.89**	**12931.85**
	40%	7675.23	10616.85	8162.22	**10130.12**	8032.65	9464.50	**7893.06**	**20875.20**	14923.05
9	5%	7677.90	**7576.63**	7569.77	7575.30	7569.49	7574.00	**7569.21**	**9060.94**	8403.68
	10%	7673.86	**7581.91**	7542.72	7573.77	7541.30	7565.68	**7539.96**	**10458.87**	9165.22
	15%	7677.05	7689.32	7548.37	7659.88	7543.21	**7629.38**	**7538.27**	**12507.88**	10301.18
	20%	7677.31	7876.64	7569.43	7812.62	7557.71	7743.17	**7546.35**	**13841.15**	11033.01
	30%	7673.94	8765.93	7718.76	8553.32	7672.02	8296.14	**7624.49**	**17270.03**	**12933.14**
	40%	7677.31	10614.87	8161.61	**10128.32**	8032.18	9463.00	**7892.74**	**20871.48**	14919.99

Table 26 shows that, for all error variances and missing value percentages of platinum loss with SRS, the AR-MRQ3 method achieved the minimum mean square error.

**Table 27:** Mean absolute percentage errors of platinum loss for the scenarios with error variances of 1, 3, 5, 7, and 9; a sample sizes of 48; and missing value percentages of 5, 10, 15, 20, 30, and 40% with SRS in missing value estimation methods.

Sample	Missing	Listwise	Missing value estimation methods							
sizes	values	delition	R-RQ1	R-RQ3	AR- CRQ1	AR-CRQ3	AR-MRQ1	AR-MRQ3	AR-MCRQ1	AR-MCRQ3
1	5%	1845.38	**1920.83**	1921.92	1921.00	1921.99	1921.17	**1922.05**	**1858.38**	1874.67
a	10%	1860.95	**1916.41**	1924.01	1917.59	1924.48	1918.85	**1924.95**	**1793.11**	1827.52
	15%	1878.49	1898.22	1918.64	1901.38	1919.93	**1904.91**	**1921.29**	**1715.01**	1769.94
	20%	1879.69	1875.52	1915.06	1881.59	1917.70	1888.77	**1920.52**	**1637.16**	1714.64
	30%	1880.03	1775.83	1865.33	1789.46	1871.99	1807.46	**1879.46**	**1474.10**	**1591.35**
	40%	1893.33	1660.80	1804.83	**1682.64**	1816.94	1715.29	**1831.37**	**1354.14**	1502.12
3	5%	1845.35	**1920.77**	1921.86	1920.94	1921.93	1921.12	**1921.99**	**1858.34**	1874.61
	10%	1860.37	**1915.75**	1923.34	1916.93	1923.80	1918.19	**1924.28**	**1792.74**	1827.06
	15%	1880.44	1900.05	1920.47	1903.20	1921.77	**1906.74**	**1923.12**	**1716.79**	1771.73
	20%	1881.02	1876.82	1916.38	1882.90	1919.02	1890.09	**1921.84**	**1638.34**	1715.87
	30%	1881.76	1777.34	1866.96	1790.99	1873.63	1809.01	**1881.11**	**1475.21**	**1592.61**
	40%	1894.12	1661.68	1805.65	**1683.51**	1817.76	1716.14	**1832.18**	**1355.06**	1503.04
5	5%	1845.91	**1921.58**	1922.67	1921.75	1922.74	1921.92	**1922.80**	**1859.31**	1875.55
	10%	1861.13	**1916.61**	1924.21	1917.79	1924.67	1919.05	**1925.15**	**1793.35**	1827.74
	15%	1878.94	1898.78	1919.18	1901.93	1920.47	**1905.47**	**1921.83**	**1715.75**	1770.61
	20%	1879.91	1875.90	1915.50	1881.98	1918.14	1889.17	**1920.96**	**1637.16**	1714.77
	30%	1882.68	1777.99	1867.66	1791.65	1874.34	1809.68	**1881.82**	**1475.71**	**1593.17**
	40%	1891.96	1660.09	1803.85	**1681.89**	1815.94	1714.48	**1830.33**	**1353.96**	1501.72
7	5%	1846.99	**1922.85**	1923.95	1923.02	1924.02	1923.20	**1924.08**	**1859.96**	1876.37
	10%	1861.99	**1917.55**	1925.15	1918.73	1925.61	1919.99	**1926.09**	**1794.28**	1828.69
	15%	1880.43	1900.11	1920.63	1903.28	1921.94	**1906.84**	**1923.30**	**1715.87**	1771.10
	20%	1877.04	1872.98	1912.40	1879.04	1915.03	1886.20	**1917.84**	**1635.42**	1712.64
	30%	1883.56	1779.74	1869.08	1793.35	1875.74	1811.32	**1883.20**	**1478.54**	**1595.58**
	40%	1894.91	1661.91	1806.06	**1683.77**	1818.19	1716.44	**1832.62**	**1354.92**	1503.09
9	5%	1845.53	**1921.05**	1922.14	1921.22	1922.21	1921.39	**1922.27**	**1858.69**	1874.95
	10%	1861.10	**1916.38**	1923.97	1917.56	1924.43	1918.82	**1924.90**	**1793.26**	1827.63
	15%	1879.45	1899.40	1919.76	1902.55	1921.06	**1906.08**	**1922.41**	**1716.61**	1771.46
	20%	1877.75	1874.18	1913.58	1880.24	1916.22	1887.39	**1919.02**	**1636.70**	1713.91
	30%	1880.98	1776.73	1866.15	1790.35	1872.81	1808.33	**1880.27**	**1475.27**	**1592.41**
	40%	1898.07	1664.39	1808.93	**1686.31**	1821.08	1719.07	**1835.56**	**1356.58**	1505.16

Table 27 shows that, for all error variances and missing value percentages of platinum loss with SRS, the AR-MCRQ1 method achieved the minimum mean absolute percentage error.

**Table 28:** Mean square errors of platinum loss for the scenarios with error variance of 1, 3, 5, 7, and 9; a sample sizes of 48; and missing value percentages of 5, 10, 15, 20, 30, and 40% with RSS in missing value estimation methods.

Sample	Missing	Listwise	Missing value estimation methods							
sizes	values	delition	R-RQ1	R-RQ3	AR- CRQ1	AR-CRQ3	AR-MRQ1	AR-MRQ3	AR-MCRQ1	AR-MCRQ3
1	5%	2373.82	**2442.95**	2474.57	2447.80	2476.48	2452.90	**2478.42**	**2969.90**	**2231.39**
	10%	3260.79	**3059.68**	3161.23	3074.23	3167.98	3090.26	**3174.95**	**5190.85**	3804.00
	15%	3693.78	**3478.05**	3550.00	3483.29	3557.50	**3491.61**	**3565.67**	**8092.53**	5660.11
	20%	3786.14	3742.59	**3685.02**	3719.13	3688.95	3698.31	**3694.14**	**9854.84**	6758.91
	30%	3894.93	4880.93	4094.55	4710.90	4066.46	4515.63	**4039.74**	**14005.80**	**9286.46**
	40%	3956.21	6918.86	4704.15	**6471.52**	4595.95	5882.26	**4482.23**	**17908.44**	11576.70
3	5%	2373.80	**2443.80**	2475.41	2448.65	2477.32	2453.75	**2479.26**	**2971.61**	**2233.14**
	10%	3263.38	**3063.04**	3164.54	3077.58	3171.27	3093.60	**3178.25**	**5194.48**	3807.80
	15%	3696.23	**3479.94**	3551.85	3485.17	3559.35	**3493.48**	**3567.52**	**8094.40**	5662.01
	20%	3789.05	3746.26	**3688.72**	3722.80	3692.65	3702.00	**3697.84**	**9857.93**	6762.27
	30%	3896.49	4881.42	4095.32	4711.43	4067.26	4516.21	**4040.56**	**14006.41**	**9286.82**
	40%	3959.76	6920.67	4706.60	**6473.39**	4598.47	5884.26	**4484.85**	**17910.51**	11578.35
5	5%	2376.11	**2445.32**	2476.88	2450.17	2478.79	2455.25	**2480.72**	**2974.28**	**2235.78**
	10%	3265.05	**3063.84**	3165.38	3078.39	3172.12	3094.41	**3179.10**	**5195.01**	3808.22
	15%	3697.42	**3481.13**	3552.92	3486.34	3560.41	**3494.64**	**3568.57**	**8096.06**	5663.66
	20%	3789.58	3746.13	**3688.61**	3722.67	3692.54	3701.87	**3697.74**	**9858.19**	6762.33
	30%	3898.07	4883.10	4097.11	4713.12	4069.05	4517.92	**4042.36**	**14007.75**	**9288.30**
	40%	3960.17	6921.84	4707.64	**6474.56**	4599.50	5885.41	**4485.84**	**17911.08**	11579.38
7	5%	2379.02	**2449.04**	2480.67	2453.89	2482.58	2458.99	**2484.53**	**2975.79**	**2237.33**
	10%	3268.44	**3067.32**	3168.91	3081.87	3175.66	3097.91	**3182.64**	**5198.32**	3811.59
	15%	3698.20	**3483.60**	3555.33	3488.80	3562.81	**3497.09**	**3570.97**	**8098.29**	5666.24
	20%	3796.36	3750.80	**3694.19**	3727.48	3698.19	3706.83	**3703.46**	**9861.54**	6765.16
	30%	3900.56	4884.47	4098.96	4714.56	4070.94	4519.46	**4044.29**	**14008.12**	**9288.92**
	40%	3963.45	6924.75	4710.74	**6477.50**	4602.61	5888.40	**4488.98**	**17913.57**	11582.08
9	5%	2380.44	**2450.02**	2481.58	2454.86	2483.49	2459.95	**2485.43**	**2976.62**	**2238.14**
	10%	3268.43	**3068.27**	3169.98	3082.85	3176.74	3098.90	**3183.72**	**5198.24**	3811.56
	15%	3701.49	**3485.34**	3557.65	3490.63	3565.18	**3499.01**	**3573.38**	**8098.33**	5665.96
	20%	3796.18	3750.64	**3693.81**	3727.28	3697.79	3706.60	**3703.04**	**9861.21**	6765.22
	30%	3904.09	4886.82	4101.58	4716.94	4073.60	4521.87	**4046.99**	**14011.71**	**9291.64**
	40%	3965.08	6926.78	4712.75	**6479.54**	4604.61	5890.44	**4490.97**	**17915.13**	11583.97

Table 28 shows that, for all error variances and missing value percentages of platinum loss of 10, and 15% with RSS, the AR-RQ1 method achieved the minimum mean square error. For all error variances and missing value percentages of platinum loss of 20%, the AR-RQ3 method achieved the minimum mean square error. For all error variances and missing value percentage of platinum loss of 5%, the AR-MCRQ3 method achieved the minimum mean square error. Finally, the AR-MRQ3 method achieved the minimum mean square error for all error variances and higher missing value percentages.

**Table 29:** Mean absolute percentage errors of platinum loss for the scenarios with error variances of 1, 3, 5, 7, and 9; a sample sizes of 48; and missing value percentages of 5, 10, 15, 20, 30, and 40% with RSS in missing value estimation methods.

Sample	Missing	Listwise	Missing value estimation methods							
sizes	values	delition	R-RQ1	R-RQ3	AR- CRQ1	AR-CRQ3	AR-MRQ1	AR-MRQ3	AR-MCRQ1	AR-MCRQ3
1	5%	183.017	**520.402**	529.401	521.807	529.933	523.275	**530.475**	**296.273**	**356.122**
	10%	223.352	**592.429**	614.515	595.864	615.880	599.622	**617.292**	**332.981**	407.068
	15%	234.951	**597.476**	632.030	602.826	634.268	**608.970**	**636.629**	**343.127**	421.180
	20%	237.266	596.707	**642.304**	603.718	645.406	612.201	**648.749**	**359.848**	438.498
	30%	240.547	550.734	615.430	560.562	620.354	573.896	**625.943**	**353.594**	**431.945**
	40%	240.453	445.285	515.798	**455.866**	521.941	472.220	**529.335**	**306.219**	374.997
3	5%	187.325	**524.570**	533.571	525.976	534.104	527.444	**534.645**	**300.393**	**360.255**
	10%	225.155	**597.388**	619.506	600.828	620.873	604.592	**622.288**	**337.555**	411.753
	15%	236.375	**600.725**	635.266	606.072	637.503	**612.214**	**639.863**	**346.481**	424.501
	20%	239.059	598.453	**644.127**	605.474	647.235	613.972	**650.583**	**361.192**	439.975
	30%	241.799	553.824	618.577	563.660	623.506	577.004	**629.100**	**356.483**	**434.912**
	40%	241.671	445.618	515.946	**456.171**	522.073	472.481	**529.449**	**306.962**	375.532
5	5%	190.114	**528.231**	537.225	529.636	537.757	531.103	**538.298**	**304.232**	**364.046**
	10%	226.087	**602.624**	624.721	606.061	626.087	609.821	**627.500**	**343.038**	417.165
	15%	235.732	**605.057**	639.507	610.391	641.738	**616.517**	**644.092**	**351.484**	429.295
	20%	240.317	603.248	**648.883**	610.264	651.988	618.755	**655.334**	**366.197**	444.907
	30%	243.332	557.000	621.785	566.843	626.716	580.195	**632.313**	**359.584**	**438.042**
	40%	242.974	449.679	520.144	**460.254**	526.283	476.595	**533.673**	**310.741**	379.446
7	5%	196.337	**532.991**	541.989	534.396	542.521	535.864	**543.063**	**308.878**	**368.722**
	10%	231.359	**604.975**	627.098	608.415	628.465	612.180	**629.880**	**345.077**	419.293
	15%	238.882	**607.415**	641.932	612.759	644.168	**618.897**	**646.526**	**353.323**	431.296
	20%	243.216	605.512	**651.190**	612.535	654.298	621.034	**657.647**	**368.226**	447.014
	30%	245.866	557.648	622.258	567.463	627.177	580.777	**632.758**	**360.786**	**439.020**
	40%	244.057	453.691	524.394	**464.300**	530.553	480.693	**537.967**	**314.331**	383.248
9	5%	201.096	**537.112**	546.105	538.516	546.638	539.984	**547.179**	**313.109**	**372.923**
	10%	234.887	**606.881**	628.984	610.318	630.350	614.080	**631.764**	**347.217**	421.366
	15%	244.796	**611.062**	645.651	616.417	647.891	**622.567**	**650.254**	**356.446**	434.579
	20%	243.653	609.860	**655.501**	616.877	658.607	625.370	**661.953**	**372.774**	451.495
	30%	248.577	561.162	625.799	570.983	630.721	584.303	**636.305**	**364.196**	**442.473**
	40%	250.278	455.222	526.100	**465.859**	532.272	482.294	**539.704**	**315.507**	384.585

Table 29 shows that, for all error variances and missing value percentages of platinum loss with RSS, the AR-MCRQ1 method achieved the minimum mean absolute percentage error.

**Table 30:** The methods provided the minimum mean square error and mean absolute percentage error with SRS and RSS for each error variance and sample size condition.

		SRS		RSS	
Error variances	Sample sizes	MSE	MAPE	MSE	MAPE
1	Small	AR-MRQ1	AR-MCRQ1	AR-MRQ1	AR-MRQ1
	Middle	AR-MRQ1	AR-MCRQ1	AR-MRQ1	AR-MRQ1
	Large	AR-MRQ1	AR-MCRQ1	AR-MRQ1	AR-RQ1
	Huge	AR-MRQ1	AR-MCRQ1	AR-MRQ1	AR-MRQ1
3	Small	AR-MRQ1	AR-MCRQ1	AR-MCRQ3	AR-MCRQ3
	Middle	AR-MRQ1	AR-MCRQ1	AR-MCRQ3	AR-MCRQ3
	Large	AR-MRQ1	AR-MCRQ1	AR-MCRQ3	AR-MCRQ3
	Huge	AR-MRQ1	AR-MCRQ1	AR-MCRQ3	AR-MCRQ3
5	Small	AR-MCRQ3	AR-MCRQ1	AR-MCRQ3	AR-MCRQ1
	Middle	AR-MCRQ3	AR-MCRQ1	AR-MCRQ3	AR-MCRQ1
	Large	AR-MCRQ3	AR-MCRQ1	AR-MCRQ3	AR-MCRQ1
	Huge	AR-MCRQ3	AR-MCRQ1	AR-MCRQ3	AR-MCRQ1
7	Small	AR-MCRQ3	AR-MCRQ1	AR-MCRQ3	AR-MCRQ1
	Middle	AR-MCRQ3	AR-MCRQ1	AR-MCRQ3	AR-MCRQ1
	Large	AR-MCRQ3	AR-MCRQ1	AR-MCRQ3	AR-MCRQ1
	Huge	AR-MCRQ3	AR-MCRQ1	AR-MCRQ3	AR-MCRQ1
9	Small	AR-MCRQ3	AR-MCRQ1	AR-MCRQ3	AR-MCRQ1
	Middle	AR-MCRQ3	AR-MCRQ1	AR-MCRQ3	AR-MCRQ1
	Large	AR-MCRQ3	AR-MCRQ1	AR-MCRQ1	AR-MCRQ1
	Huge	AR-MCRQ3	AR-MCRQ1	AR-MCRQ1	AR-MCRQ1

Table 30 is a summary of the comparative results between the four methods. The best method, which provided the minimum MSE and MAPE for each kind of error variance and sample size, is tabulated in two columns, SRS and RSS.

AR-MRQ1 with SRS achieved the minimum mean square error for a small error variance. However, AR-MCRQ3 achieved the minimum mean square error for a large error variance. AR-MCRQ1 achieved the minimum mean absolute percentage error for all kinds of error variances.

AR-MRQ1 with RSS achieved the minimum mean square error for a small error variance. However, AR-MCRQ3 and AR-MCRQ1 achieved the minimum mean square error for a larger error variance. AR-MRQ1 achieved the minimum mean absolute percentage error for a small error variance. However, AR-MCRQ1 achieved the minimum mean absolute percentage error for a larger error variance.

**Table 31:** The methods provided the minimum mean square error and mean absolute percentage error with SRS and RSS for each error variance condition.

Error variances	SRS		RSS	
	MSE	MAPE	MSE	MAPE
Small	AR-MRQ1	AR-MCRQ1	AR-MRQ1	AR-MRQ1
Middle	AR-MCRQ3	AR-MCRQ1	AR-MCRQ3	AR-MCRQ1
Large	AR-MCRQ3	AR-MCRQ1	AR-MCRQ1, AR-MCRQ3	AR-MCRQ1

Table 31, for a small error variance, the AR-MRQ1 method with SRS and RSS provided the minimum MSE. However, for middle and large error variances, AR-MCRQ3 provided the minimum MSE. Regarding the MAPE measure, for a small error variance, AR-MCRQ1 and AR-MRQ1 with SRS and RSS provided the minimum MAPE, respectively. However, AR-MCRQ1 with SRS and RSS provided the minimum MAPE for middle and large error variances. Finally, AR-MCRQ1 was the best method for missing value imputation in multiple regression analysis, followed by AR-MCRQ3.

**Table 32:** The number of times for methods provided the smaller mean square error and mean absolute percentage error with SRS and RSS for each error variance condition.

			Smaller MSE		Smaller MAPE	
Error variances	Sample sizes	Scenarios in total	SRS	RSS	SRS	RSS
1	Small	96	11	**85**	0	**96**
	Middle	96	1	**95**	0	**96**
	Large	96	2	**94**	0	**96**
	Huge	96	0	**96**	0	**96**
3	Small	96	20	**76**	0	**96**
	Middle	96	0	**96**	0	**96**
	Large	96	5	**91**	0	**96**
	Huge	96	10	**86**	0	**96**
5	Small	96	26	**70**	0	**96**
	Middle	96	15	**81**	0	**96**
	Large	96	4	**92**	0	**96**
	Huge	96	0	**96**	0	**96**
7	Small	96	32	**64**	0	**96**
	Middle	96	8	**88**	0	**96**
	Large	96	12	**84**	0	**96**
	Huge	96	14	**82**	8	**88**
9	Small	96	26	**70**	0	**96**
	Middle	96	22	**74**	0	**96**
	Large	96	17	**79**	0	**96**
	Huge	96	20	**76**	0	**96**

Bold values indicate the better SRS or RSS estimators when comparing the smaller MSE and MAPE. Each sample size provided two values, the proportions of missing values provided six values, and missing value estimation methods provided eight values. There were 2x6x8 = 96 scenarios in total for each sampling.

Table 32, for all error variances, the sample sizes were increased, and the number of times for methods was increased with the RSS estimators providing smaller MSE than the SRS estimators. Moreover, the RSS estimators provided smaller MSE and MAPE than the SRS estimators for all error variances and sample sizes.

## 4. Discussion

This research compared the efficiency of four new adjusted missing value imputations in multiple regression analysis. The study results show that AR-MRQ1 with SRS achieved the minimum mean square error for a small error variance. However, AR-MCRQ3 achieved the minimum mean square error for a large error variance. For all error variances in mean absolute percentage error, AR-MCRQ1 achieved the minimum mean absolute percentage error. The results of this study were similar to those reported in [14, 15]. In those papers, the proposed estimator was a multivariate chain ratio (MCR) and a regression estimator that used a linear combination of two auxiliary variables. The MCR and regression estimators that used the two auxiliary variables were equally highly effective, followed by the traditional multivariate ratio (MR) and regression estimators that used the two auxiliary variables. This study was also consistent with [11] in that the chain ratio (CR) estimator with SRS was more efficient than the traditional ratio estimator under certain conditions. Our results may be contrasted to [22] which reported that the regression-ratio Q1 (R-RQ1) estimator was the most efficient. This discrepancy was because the study did not include AR-CRQ1,3, AR-MRQ1,3, and AR-MCRQ1,3 estimators in the test.

AR-MRQ1 achieved the minimum mean square error for RSS and small error variance. However, AR-MCRQ3 achieved the minimum mean square error for a large error variance, followed by AR-MCRQ1. AR-MRQ1 achieved the minimum mean absolute percentage error for a small error variance. However, AR-MCRQ1 achieved the minimum mean absolute percentage error for a large error variance. To the best of the author’s knowledge, RSS has not been studied before.

Moreover, the RSS estimators provided smaller mean square error and mean absolute percentage error than the SRS estimators for the same error variances, sample sizes, and missing value percentages. Therefore, the RSS estimators were more efficient than the SRS estimators. Our results agree well with those reported by [3–5, 13]: the RSS estimators were more efficient than the SRS estimators for the same quartile, coefficient of correlation, and sample size.

## 5. Conclusion

This study compared the efficiencies of four new adjusted missing value imputation methods in multiple regression analysis. The four estimation methods were the following: a regression-ratio quartile1,3 (R-RQ1,3) imputation of Al-Omari, Jemain, and Ibrahim; an adjusted regression-chain ratio quartile1,3 (AR-CRQ1,3) imputation of Kadilar and Cinji; an adjusted regression-multivariate ratio quatile1,3 (AR-MRQ1,3) imputation of Feng, Ni, and Zou; and an adjusted regression-multivariate chain ratio quartile1,3 (AR-MCRQ1,3) imputation of Lu for each simple random sampling (SRS) and rank set sampling (RSS). The measures for comparing the performance were mean square error (MSE) and mean absolute percentage error (MAPE).

## Future recommendations

5.1 The random forest imputation may be a suitable method for estimating missing values imputations in multiple regression analysis [21]. A comparative study should be conducted between this imputation method and the author’s method.

5.2 Many kinds of variables can have missing values. We may consider missing value imputation for the dependent variable or that for both the independent and dependent variables [30]. An attempt at imputation of missing values for the dependent variable and for both the independent and dependent variables, although it will be extensive work, should be considered.

## Supporting information

S1 Code The data used to support this study were simulated from a normal distribution using the R studio program. (ZIP)

S2 Code The data used to support this study were simulated from a normal distribution using the R studio program. (ZIP)

Actual Data The data used for gold and platinum loss by customer groups. (PDF)
