# Supplementary material for: New adjusted missing value imputation in multiple regression with simple random sampling and rank set sampling methods
Source: PLoS One. 2025 Mar 17;20(3):e0316641. doi: 10.1371/journal.pone.0316641 (PMC11913305; doi:10.1371/journal.pone.0316641)
Supplement: S1 Code — (ZIP) [file pone.0316641.s001.zip › Simulation code1 - SRS.pdf]

```
# Sample size

# E=1,2,3,4,5

# Missing

set.seed(5)

N=20

e=1

# Missing value percentages

PMD = 5

# Alpha, R_CRQ1,R_CRQ3

Alpha_R_CRQ=0.9

# e, R_MRQ1,R_MRQ3

e1_R_MRQ=0.9

e2_R_MRQ=1-e1_R_MRQ

# Alpha, R_MCRQ1,R_MCRQ3

Alpha_R_MCRQ=0.1

W1_R_MCRQ=0.1

W2_R_MCRQ=1-W1_R_MCRQ


m=1000

sum_mse_REG <- 0

sum_mse_RRQ1 <- 0

sum_mse_RRQ3 <- 0

sum_mse_CRQ1 <- 0
```

```
sum_mse_CRQ3 <- 0

sum_mse_MRQ1 <- 0

sum_mse_MRQ3 <- 0

sum_mse_MCRQ1 <- 0

sum_mse_MCRQ3 <- 0


sum_mape_REG <- 0

sum_mape_RRQ1 <- 0

sum_mape_RRQ3 <- 0

sum_mape_CRQ1 <- 0

sum_mape_CRQ3 <- 0

sum_mape_MRQ1 <- 0

sum_mape_MRQ3 <- 0

sum_mape_MCRQ1 <- 0

sum_mape_MCRQ3 <- 0
```

```
for (j in 1:m) {

  # Construct population X1
  X1 = rnorm(100000,3,sqrt(1.5))
  print(X1)
  # Construct population X2
  X2 = rnorm(100000,5,sqrt(2))
  print(X2)
  # Construct population E
  Data_e = sqrt(e)
  E = rnorm(100000,mean=0,sd=Data_e)
  print(E)

  # Random variable X1
  Sample_X1 = sample(X1, size=N,)
  print(Sample_X1)
  Data_X1=Sample_X1
  # Random variable X2
  Sample_X2 = sample(X2, size=N,)
  print(Sample_X2)
  Data_X2=Sample_X2
```

```
# Random variable E
Sample_E = sample(E, size=N,)
print(Sample_E)
Data_E=Sample_E

# Correlation
X_correlation = cbind(Data_X1,Data_X2)
cor(X_correlation)

# Construct variable Y
Y = function(Data_X1,Data_X2,Data_e) {
  0.5+(1*(Data_X1))+(-0.3*(Data_X2)+Data_e)
}
Y(Data_X1,Data_X2,Data_e)

# Calculate count of missing value
NMD = function(N,PMD) {
  (N)*(PMD)/100
}
NMD(N,PMD)# Count of missing value

# Calculate position of missing value
N_MD = NMD(N,PMD)
PM=sample(N,size = N_MD)
PM
```

```
# Esimate missing value_RRQ1

Data_Xi_REG=rep(1,N)

Data_Xi_REG

Data_Y_REG = Y(Data_X1,Data_X2,Data_e)

Data_X_REG = c(Data_Xi_REG, Data_X1, Data_X2)

matrix_X_REG <- matrix(Data_X_REG,nrow = N,ncol = 3)

matrix_X_REG

matrix_Y_REG <- matrix(Data_Y_REG,nrow = N,ncol = 1)

matrix_Y_REG

# Estimate missing value

New_Estimate_REG = (sum(Data_X2)-(matrix_X_REG[PM,3]))/(N-N_MD)

New_Estimate_REG

Data_X2_REG_New=replace(Data_X2,list= PM,values = New_Estimate_REG)

Data_X2_REG_New

# Construct matrix after estimate missing value

Data_X_REG_New = c(Data_Xi_REG, Data_X1, Data_X2_REG_New)

matrix_X_REG_New <- matrix(Data_X_REG_New,nrow = N,ncol = 3)

matrix_X_REG_New

# X'_REG_New

Transpose_X_REG_New = t(matrix_X_REG_New)

Transpose_X_REG_New

# X'X_REG_New

TransposeX_X_REG_New = Transpose_X_REG_New%*%matrix_X_REG_New

TransposeX_X_REG_New

# X'Y_REG_New
```

```
TransposeX_Y_REG_New = Transpose_X_REG_New%*%matrix_Y_REG
TransposeX_Y_REG_New
# Inverse(X'X)_REG_New
Inverse_TransposeX_X_REG_New = solve(TransposeX_X_REG_New)
Inverse_TransposeX_X_REG_New
# B_REG_New
B_REG_New = Inverse_TransposeX_X_REG_New%*%TransposeX_Y_REG_New
B_REG_New
# Equation model after estimate missing value
cat("Yhat=",B_REG_New[1,1],"+",B_REG_New[2,1],"X1+",B_REG_New[3,1],"X2+e")
# Y after estimate missing value
Y_REG_New = function(B_REG_New,Data_X1,Data_X2_REG_New,Data_E) {
  B_REG_New[1,1]+(B_REG_New[2,1]*Data_X1)+(B_REG_New[3,1]*Data_X2_REG_New)+Data_E
}
Y_REG_New(B_REG_New,Data_X1,Data_X2_REG_New,Data_E)
matrix_Y_REG_New <- matrix(Y_REG_New(B_REG_New,Data_X1,Data_X2_REG_New,Data_E),nrow =
N,ncol = 1)
matrix_Y_REG_New
Data_Y_REG_New=Y_REG_New(B_REG_New,Data_X1,Data_X2_REG_New,Data_E)
matrix_Y_REG_New <- matrix(Data_Y_REG_New,nrow = N,ncol = 1)
matrix_Y_REG_New
# Position of Q1
R1=1
RRQ1=function(N,R1) {
  (R1*(N+1))/4
}
```

```
RRQ1(N,R1)

# Data position of Q1

P_RRQ1=RRQ1(N,R1)

Data_X2_Sort=sort(Data_X2)

Data_X2_Sort

matrix_Data_X2_Sort <- matrix(Data_X2_Sort,nrow = N,ncol = 1)

matrix_Data_X2_Sort

Data_X2_RRQ1 = matrix_Data_X2_Sort[P_RRQ1,1]

Data_X2_RRQ1

New_Estimate_RRQ1 = (matrix_Y_REG_New[PM,1])*((mean(Data_X2)+Data_X2_RRQ1)/(((sum(Data_X2)-
(matrix_X_REG[PM,3]))/(N-N_MD))+Data_X2_RRQ1))

New_Estimate_RRQ1

Data_Y_RRQ1_New=replace(Data_Y_REG_New,list= PM,values = New_Estimate_RRQ1)

Data_Y_RRQ1_New

# MSE_RRQ1

matrix_Y_RRQ1_New <- matrix(Data_Y_RRQ1_New,nrow = N,ncol = 1)

matrix_Y_RRQ1_New

MSE_RRQ1 = function(N,matrix_Y_REG,matrix_Y_RRQ1_New) {

  (1/N)*(sum((matrix_Y_REG-matrix_Y_RRQ1_New)^2))

}

MSE_RRQ1(N,matrix_Y_REG,matrix_Y_RRQ1_New)

# MAPE_RRQ1

MAPE_RRQ1 = function(N,matrix_Y_REG,matrix_Y_RRQ1_New) {
```

```
  (1/N)*(sum((abs(matrix_Y_REG-matrix_Y_RRQ1_New)/abs(matrix_Y_REG))*100))
}
MAPE_RRQ1(N,matrix_Y_REG,matrix_Y_RRQ1_New)



# Estimate missing value_RRQ3
Data_Y_REG_New=Y_REG_New(B_REG_New,Data_X1,Data_X2_REG_New,Data_E)
matrix_Y_REG_New <- matrix(Data_Y_REG_New,nrow = N,ncol = 1)
matrix_Y_REG_New
# Position of Q3
R3=3
RRQ3=function(N,R3) {
  (R3*(N+1))/4
}
RRQ3(N,R3)
# Data position of Q3
P_RRQ3=RRQ3(N,R3)
Data_X2_Sort=sort(Data_X2)
Data_X2_Sort
matrix_X2_Sort <- matrix(Data_X2_Sort,nrow = N,ncol = 1)
matrix_X2_Sort
Data_X2_RRQ3 = matrix_X2_Sort[P_RRQ3,1]
Data_X2_RRQ3
```

```
New_Estimate_RRQ3 = (matrix_Y_REG_New[PM,1])*((mean(Data_X2)+Data_X2_RRQ3)/(((sum(Data_X2)-(matrix_X_REG[PM,3]))/(N-N_MD))+Data_X2_RRQ3))

New_Estimate_RRQ3

Data_Y_RRQ3_New=replace(Data_Y_REG_New,list= PM,values = New_Estimate_RRQ3)

Data_Y_RRQ3_New

# MSE_RRQ3

matrix_Y_RRQ3_New <- matrix(Data_Y_RRQ3_New,nrow = N,ncol = 1)

matrix_Y_RRQ3_New

MSE_RRQ3 = function(N,matrix_Y_REG,matrix_Y_RRQ3_New) {

  (1/N)*(sum((matrix_Y_REG-matrix_Y_RRQ3_New)^2))

}

MSE_RRQ3(N,matrix_Y_REG,matrix_Y_RRQ3_New)

# MAPE_RRQ3

MAPE_RRQ3 = function(N,matrix_Y_REG,matrix_Y_RRQ3_New) {

  (1/N)*(sum((abs(matrix_Y_REG-matrix_Y_RRQ3_New)/abs(matrix_Y_REG))*100))

}

MAPE_RRQ3(N,matrix_Y_REG,matrix_Y_RRQ3_New)
```

```
# Estimate missing value_R_CRQ1

Data_Y_REG_New=Y_REG_New(B_REG_New,Data_X1,Data_X2_REG_New,Data_E)

matrix_Y_REG_New <- matrix(Data_Y_REG_New,nrow = N,ncol = 1)

matrix_Y_REG_New

# Position of Q1

R1=1

RRQ1=function(N,R1) {

  (R1*(N+1))/4

}

RRQ1(N,R1)

# Data position of Q1

P_RRQ1=RRQ1(N,R1)

Data_X2_Sort=sort(Data_X2)

Data_X2_Sort

matrix_Data_X2_Sort <- matrix(Data_X2_Sort,nrow = N,ncol = 1)

matrix_Data_X2_Sort

Data_X2_RRQ1 = matrix_Data_X2_Sort[P_RRQ1,1]

Data_X2_RRQ1

New_Estimate_R_CRQ1 =
(matrix_Y_REG_New[PM,1])*(((mean(Data_X2)+Data_X2_RRQ1)/(((sum(Data_X2)-
(matrix_X_REG[PM,3]))/(N-N_MD))+Data_X2_RRQ1))^Alpha_R_CRQ)

New_Estimate_R_CRQ1
```

```
Data_Y_R_CRQ1_New=replace(Data_Y_REG,list= PM,values = New_Estimate_R_CRQ1)

Data_Y_R_CRQ1_New

# MSE_R_CRQ1

matrix_Y_R_CRQ1_New <- matrix(Data_Y_R_CRQ1_New,nrow = N,ncol = 1)

matrix_Y_R_CRQ1_New

MSE_R_CRQ1 = function(N,matrix_Y_REG,matrix_Y_R_CRQ1_New) {

  (1/N)*(sum((matrix_Y_REG-matrix_Y_R_CRQ1_New)^2))

}

MSE_R_CRQ1(N,matrix_Y_REG,matrix_Y_R_CRQ1_New)

# MAPE_CRQ1

MAPE_R_CRQ1 = function(N,matrix_Y_REG,matrix_Y_R_CRQ1_New) {

  (1/N)*(sum((abs(matrix_Y_REG-matrix_Y_R_CRQ1_New)/abs(matrix_Y_REG))*100))

}

MAPE_R_CRQ1(N,matrix_Y_REG,matrix_Y_R_CRQ1_New)
```

```
# Estimate missing value_R_CRQ3

Data_Y_REG_New=Y_REG_New(B_REG_New,Data_X1,Data_X2_REG_New,Data_E)

matrix_Y_REG_New <- matrix(Data_Y_REG_New,nrow = N,ncol = 1)

matrix_Y_REG_New

# Position of Q3

R3=3

RRQ3=function(N,R3) {

  (R3*(N+1))/4

}

RRQ3(N,R3)

# Data position of Q3

P_RRQ3=RRQ3(N,R3)

Data_X2_Sort=sort(Data_X2)

Data_X2_Sort

matrix_X2_Sort <- matrix(Data_X2_Sort,nrow = N,ncol = 1)

matrix_X2_Sort

Data_X2_RRQ3 = matrix_X2_Sort[P_RRQ3,1]

Data_X2_RRQ3

New_Estimate_R_CRQ3 =
(matrix_Y_REG_New[PM,1])*(((mean(Data_X2)+Data_X2_RRQ3)/(((sum(Data_X2)-
(matrix_X_REG[PM,3]))/(N-N_MD))+Data_X2_RRQ3))^Alpha_R_CRQ)

New_Estimate_R_CRQ3
```

```
Data_Y_R_CRQ3_New=replace(Data_Y_REG,list= PM,values = New_Estimate_R_CRQ3)

Data_Y_R_CRQ3_New

# MSE_R_CRQ3

matrix_Y_R_CRQ3_New <- matrix(Data_Y_R_CRQ3_New,nrow = N,ncol = 1)

matrix_Y_R_CRQ3_New

MSE_R_CRQ3 = function(N,matrix_Y_REG,matrix_Y_R_CRQ3_New) {

  (1/N)*(sum((matrix_Y_REG-matrix_Y_R_CRQ3_New)^2))

}

MSE_R_CRQ3(N,matrix_Y_REG,matrix_Y_R_CRQ3_New)

# MAPE_CRQ3

MAPE_R_CRQ3 = function(N,matrix_Y_REG,matrix_Y_R_CRQ3_New) {

  (1/N)*(sum((abs(matrix_Y_REG-matrix_Y_R_CRQ3_New)/abs(matrix_Y_REG))*100))

}

MAPE_R_CRQ3(N,matrix_Y_REG,matrix_Y_R_CRQ3_New)
```

```
# Estimate missing value_R_MRQ1

Data_Y_REG_New=Y_REG_New(B_REG_New,Data_X1,Data_X2_REG_New,Data_E)

matrix_Y_REG_New <- matrix(Data_Y_REG_New,nrow = N,ncol = 1)

matrix_Y_REG_New

# Position of Q1

R1=1

RRQ1=function(N,R1) {

  (R1*(N+1))/4

}

RRQ1(N,R1)

# Data position of Q1

P_RRQ1=RRQ1(N,R1)

Data_X1_Sort=sort(Data_X1)

Data_X1_Sort

matrix_Data_X1_Sort <- matrix(Data_X1_Sort,nrow = N,ncol = 1)

Data_X1_RRQ1 = matrix_Data_X1_Sort[P_RRQ1,1]

Data_X1_RRQ1

Data_X2_Sort=sort(Data_X2)

Data_X2_Sort

matrix_Data_X2_Sort <- matrix(Data_X2_Sort,nrow = N,ncol = 1)

matrix_Data_X2_Sort

Data_X2_RRQ1 = matrix_Data_X2_Sort[P_RRQ1,1]
```

```
Data_X2_RRQ1

New_Estimate_R_MRQ1 =
(matrix_Y_REG_New[PM,1])*((e2_R_MRQ*((mean(X2)+Data_X2_RRQ1)/(((sum(X2)-
(matrix_X_REG[PM,3]))/(N-
N_MD))+Data_X2_RRQ1)))+(e1_R_MRQ*((mean(X1)+Data_X1_RRQ1)/((mean(X1)+Data_X1_RRQ1)))))

Data_Y_R_MRQ1_New=replace(Data_Y_REG,list= PM,values = New_Estimate_R_MRQ1)

Data_Y_R_MRQ1_New

# MSE_R_MRQ1

matrix_Y_R_MRQ1_New <- matrix(Data_Y_R_MRQ1_New,nrow = N,ncol = 1)

matrix_Y_R_MRQ1_New

MSE_R_MRQ1 = function(N,matrix_Y_REG,matrix_Y_R_MRQ1_New) {

  (1/N)*(sum((matrix_Y_REG-matrix_Y_R_MRQ1_New)^2))

}

MSE_R_MRQ1(N,matrix_Y_REG,matrix_Y_R_MRQ1_New)

# MAPE_MRQ1

MAPE_R_MRQ1 = function(N,matrix_Y_REG,matrix_Y_R_MRQ1_New) {

  (1/N)*(sum((abs(matrix_Y_REG-matrix_Y_R_MRQ1_New)/abs(matrix_Y_REG))*100))

}

MAPE_R_MRQ1(N,matrix_Y_REG,matrix_Y_R_MRQ1_New)
```

```
# Estimate missing value_R_MRQ3

Data_Y_REG_New=Y_REG_New(B_REG_New,Data_X1,Data_X2_REG_New,Data_E)

matrix_Y_REG_New <- matrix(Data_Y_REG_New,nrow = N,ncol = 1)

matrix_Y_REG_New

# Position of Q3

R3=3

RRQ3=function(N,R3) {

  (R3*(N+1))/4

}

RRQ3(N,R3)

# Data position of Q3

P_RRQ3=RRQ3(N,R3)

Data_X1_Sort=sort(Data_X1)

Data_X1_Sort

matrix_Data_X1_Sort <- matrix(Data_X1_Sort,nrow = N,ncol = 1)

Data_X1_RRQ3 = matrix_Data_X1_Sort[P_RRQ3,1]

Data_X1_RRQ3

Data_X2_Sort=sort(Data_X2)

Data_X2_Sort

matrix_Data_X2_Sort <- matrix(Data_X2_Sort,nrow = N,ncol = 1)

matrix_Data_X2_Sort

Data_X2_RRQ3 = matrix_Data_X2_Sort[P_RRQ3,1]
```

```
Data_X2_RRQ3

New_Estimate_R_MRQ3 =
(matrix_Y_REG_New[PM,1])*((e2_R_MRQ*((mean(X2)+Data_X2_RRQ3)/(((sum(X2)-
(matrix_X_REG[PM,3]))/(N-
N_MD))+Data_X2_RRQ3)))+(e1_R_MRQ*((mean(X1)+Data_X1_RRQ3)/((mean(X1)+Data_X1_RRQ3)))))

Data_Y_R_MRQ3_New=replace(Data_Y_REG,list= PM,values = New_Estimate_R_MRQ3)

Data_Y_R_MRQ3_New

# MSE_R_MRQ3

matrix_Y_R_MRQ3_New <- matrix(Data_Y_R_MRQ3_New,nrow = N,ncol = 1)

matrix_Y_R_MRQ3_New

MSE_R_MRQ3 = function(N,matrix_Y_REG,matrix_Y_R_MRQ3_New) {

  (1/N)*(sum((matrix_Y_REG-matrix_Y_R_MRQ3_New)^2))

}

MSE_R_MRQ3(N,matrix_Y_REG,matrix_Y_R_MRQ3_New)

# MAPE_MRQ3

MAPE_R_MRQ3 = function(N,matrix_Y_REG,matrix_Y_R_MRQ3_New) {

  (1/N)*(sum((abs(matrix_Y_REG-matrix_Y_R_MRQ3_New)/abs(matrix_Y_REG))*100))

}

MAPE_R_MRQ3(N,matrix_Y_REG,matrix_Y_R_MRQ3_New)
```

```
# Estimate missing value_R_MCRQ1

Data_Y_REG_New=Y_REG_New(B_REG_New,Data_X1,Data_X2_REG_New,Data_E)

matrix_Y_REG_New <- matrix(Data_Y_REG_New,nrow = N,ncol = 1)

matrix_Y_REG_New
# Position of Q1

R1=1

RRQ1=function(N,R1) {

  (R1*(N+1))/4

}

RRQ1(N,R1)

# Data position of Q1

P_RRQ1=RRQ1(N,R1)

Data_X1_Sort=sort(Data_X1)

Data_X1_Sort

matrix_Data_X1_Sort <- matrix(Data_X1_Sort,nrow = N,ncol = 1)

Data_X1_RRQ1 = matrix_Data_X1_Sort[P_RRQ1,1]

Data_X1_RRQ1

Data_X2_Sort=sort(Data_X2)

Data_X2_Sort

matrix_Data_X2_Sort <- matrix(Data_X2_Sort,nrow = N,ncol = 1)

matrix_Data_X2_Sort

Data_X2_RRQ1 = matrix_Data_X2_Sort[P_RRQ1,1]
```

```
Data_X2_RRQ1

New_Estimate_R_MCRQ1 =
(matrix_Y_REG_New[PM,1])*((((W2_R_MCRQ*(mean(X2)+Data_X2_RRQ1))+(W1_R_MCRQ*((mean(X1)+
Data_X1_RRQ1))))/((W2_R_MCRQ*(sum(X2)-
(matrix_X_REG[PM,3])))+(W1_R_MCRQ*(mean(X1)+Data_X1_RRQ1))))^Alpha_R_MCRQ)

Data_Y_R_MCRQ1_New=replace(Data_Y_REG,list= PM,values = New_Estimate_R_MCRQ1)

Data_Y_R_MCRQ1_New

# MSE_R_MCRQ1

matrix_Y_R_MCRQ1_New <- matrix(Data_Y_R_MCRQ1_New,nrow = N,ncol = 1)

matrix_Y_R_MCRQ1_New

MSE_R_MCRQ1 = function(N,matrix_Y_REG,matrix_Y_R_MCRQ1_New) {

  (1/N)*(sum((matrix_Y_REG-matrix_Y_R_MCRQ1_New)^2))

}

MSE_R_MCRQ1(N,matrix_Y_REG,matrix_Y_R_MCRQ1_New)

# MAPE_MCRQ1

MAPE_R_MCRQ1 = function(N,matrix_Y_REG,matrix_Y_R_MCRQ1_New) {

  (1/N)*(sum((abs(matrix_Y_REG-matrix_Y_R_MCRQ1_New)/abs(matrix_Y_REG))*100))

}

MAPE_R_MCRQ1(N,matrix_Y_REG,matrix_Y_R_MCRQ1_New)
```

```
# Estimate missing value_R_MCRQ3

Data_Y_REG_New=Y_REG_New(B_REG_New,Data_X1,Data_X2_REG_New,Data_E)

matrix_Y_REG_New <- matrix(Data_Y_REG_New,nrow = N,ncol = 1)

matrix_Y_REG_New

# Position of Q3

R3=3

RRQ3=function(N,R3) {

  (R3*(N+1))/4

}

RRQ3(N,R3)

# Data position of Q3

P_RRQ3=RRQ3(N,R3)

Data_X1_Sort=sort(Data_X1)

Data_X1_Sort

matrix_Data_X1_Sort <- matrix(Data_X1_Sort,nrow = N,ncol = 1)

Data_X1_RRQ3 = matrix_Data_X1_Sort[P_RRQ3,1]

Data_X1_RRQ3

Data_X2_Sort=sort(Data_X2)

Data_X2_Sort

matrix_Data_X2_Sort <- matrix(Data_X2_Sort,nrow = N,ncol = 1)

matrix_Data_X2_Sort

Data_X2_RRQ3 = matrix_Data_X2_Sort[P_RRQ3,1]
```

```
Data_X2_RRQ3

New_Estimate_R_MCRQ3
=(matrix_Y_REG_New[PM,1])*((((W2_R_MCRQ*(mean(X2)+Data_X2_RRQ3))+(W1_R_MCRQ*((mean(X1)+
Data_X1_RRQ3))))/((W2_R_MCRQ*(sum(X2)-
(matrix_X_REG[PM,3])))+(W1_R_MCRQ*(mean(X1)+Data_X1_RRQ3))))^Alpha_R_MCRQ)

Data_Y_R_MCRQ3_New=replace(Data_Y_REG,list= PM,values = New_Estimate_R_MCRQ3)

Data_Y_R_MCRQ3_New

# MSE_R_MCRQ3

matrix_Y_R_MCRQ3_New <- matrix(Data_Y_R_MCRQ3_New,nrow = N,ncol = 1)

matrix_Y_R_MCRQ3_New

MSE_R_MCRQ3 = function(N,matrix_Y_REG,matrix_Y_R_MCRQ3_New) {

  (1/N)*(sum((matrix_Y_REG-matrix_Y_R_MCRQ3_New)^2))

}

MSE_R_MCRQ3(N,matrix_Y_REG,matrix_Y_R_MCRQ3_New)

# MAPE_MCRQ3

MAPE_R_MCRQ3 = function(N,matrix_Y_REG,matrix_Y_R_MCRQ3_New) {

  (1/N)*(sum((abs(matrix_Y_REG-matrix_Y_R_MCRQ3_New)/abs(matrix_Y_REG))*100))

}

MAPE_R_MCRQ3(N,matrix_Y_REG,matrix_Y_R_MCRQ3_New)
```

```
RRQ1_MSE=MSE_RRQ1(N,matrix_Y_REG,matrix_Y_RRQ1_New)

sum_mse_RRQ1 = sum_mse_RRQ1+RRQ1_MSE

RRQ3_MSE=MSE_RRQ3(N,matrix_Y_REG,matrix_Y_RRQ3_New)

sum_mse_RRQ3 = sum_mse_RRQ3+RRQ3_MSE

CRQ1_MSE=MSE_R_CRQ1(N,matrix_Y_REG,matrix_Y_R_CRQ1_New)

sum_mse_CRQ1 = sum_mse_CRQ1+CRQ1_MSE

CRQ3_MSE=MSE_R_CRQ3(N,matrix_Y_REG,matrix_Y_R_CRQ3_New)

sum_mse_CRQ3 = sum_mse_CRQ3+CRQ3_MSE

MRQ1_MSE=MSE_R_MRQ1(N,matrix_Y_REG,matrix_Y_R_MRQ1_New)

sum_mse_MRQ1 = sum_mse_MRQ1+MRQ1_MSE

MRQ3_MSE=MSE_R_MRQ3(N,matrix_Y_REG,matrix_Y_R_MRQ3_New)

sum_mse_MRQ3 = sum_mse_MRQ3+MRQ3_MSE

MCRQ1_MSE=MSE_R_MCRQ1(N,matrix_Y_REG,matrix_Y_R_MCRQ1_New)

sum_mse_MCRQ1 = sum_mse_MCRQ1+MCRQ1_MSE

MCRQ3_MSE=MSE_R_MCRQ3(N,matrix_Y_REG,matrix_Y_R_MCRQ3_New)

sum_mse_MCRQ3 = sum_mse_MCRQ3+MCRQ3_MSE


RRQ1_MAPE=MAPE_RRQ1(N,matrix_Y_REG,matrix_Y_RRQ1_New)

sum_mape_RRQ1 = sum_mape_RRQ1+RRQ1_MAPE

RRQ3_MAPE=MAPE_RRQ3(N,matrix_Y_REG,matrix_Y_RRQ3_New)

sum_mape_RRQ3 = sum_mape_RRQ3+RRQ3_MAPE

CRQ1_MAPE=MAPE_R_CRQ1(N,matrix_Y_REG,matrix_Y_R_CRQ1_New)
```

```
  sum_mape_CRQ1 = sum_mape_CRQ1+CRQ1_MAPE

  CRQ3_MAPE=MAPE_R_CRQ3(N,matrix_Y_REG,matrix_Y_R_CRQ3_New)

  sum_mape_CRQ3 = sum_mape_CRQ3+CRQ3_MAPE

  MRQ1_MAPE=MAPE_R_MRQ1(N,matrix_Y_REG,matrix_Y_R_MRQ1_New)

  sum_mape_MRQ1 = sum_mape_MRQ1+MRQ1_MAPE

  MRQ3_MAPE=MAPE_R_MRQ3(N,matrix_Y_REG,matrix_Y_R_MRQ3_New)

  sum_mape_MRQ3 = sum_mape_MRQ3+MRQ3_MAPE

  MCRQ1_MAPE=MAPE_R_MCRQ1(N,matrix_Y_REG,matrix_Y_R_MCRQ1_New)

  sum_mape_MCRQ1 = sum_mape_MCRQ1+MCRQ1_MAPE

  MCRQ3_MAPE=MAPE_R_MCRQ3(N,matrix_Y_REG,matrix_Y_R_MCRQ3_New)

  sum_mape_MCRQ3 = sum_mape_MCRQ3+MCRQ3_MAPE


  cat(c("loop :",j),fill=T)
}



AVR_MSE_RRQ1 = sum_mse_RRQ1/m

AVR_MSE_RRQ1

AVR_MSE_RRQ3 = sum_mse_RRQ3/m

AVR_MSE_RRQ3

AVR_MSE_CRQ1 = sum_mse_CRQ1/m

AVR_MSE_CRQ1

AVR_MSE_CRQ3 = sum_mse_CRQ3/m
```

AVR_MSE_CRQ3

AVR_MSE_MRQ1 = sum_mse_MRQ1/m

AVR_MSE_MRQ1

AVR_MSE_MRQ3 = sum_mse_MRQ3/m

AVR_MSE_MRQ3

AVR_MSE_MCRQ1 = sum_mse_MCRQ1/m

AVR_MSE_MCRQ1

AVR_MSE_MCRQ3 = sum_mse_MCRQ3/m

AVR_MSE_MCRQ3

AVR_MAPE_RRQ1 = sum_mape_RRQ1/m

AVR_MAPE_RRQ1

AVR_MAPE_RRQ3 = sum_mape_RRQ3/m

AVR_MAPE_RRQ3

AVR_MAPE_CRQ1 = sum_mape_CRQ1/m

AVR_MAPE_CRQ1

AVR_MAPE_CRQ3 = sum_mape_CRQ3/m

AVR_MAPE_CRQ3

AVR_MAPE_MRQ1 = sum_mape_MRQ1/m

AVR_MAPE_MRQ1

AVR_MAPE_MRQ3 = sum_mape_MRQ3/m

AVR_MAPE_MRQ3

AVR_MAPE_MCRQ1 = sum_mape_MCRQ1/m

AVR_MAPE_MCRQ1

AVR_MAPE_MCRQ3 = sum_mape_MCRQ3/m

AVR_MAPE_MCRQ3
