# Supplementary material for: New adjusted missing value imputation in multiple regression with simple random sampling and rank set sampling methods
Source: PLoS One. 2025 Mar 17;20(3):e0316641. doi: 10.1371/journal.pone.0316641 (PMC11913305; doi:10.1371/journal.pone.0316641)
Supplement: S2 Code — (ZIP) [file pone.0316641.s002.zip › Simulation code2 - RRS.pdf]

```r
# Sample size
# E=1,2,3,4,5
# Missing
set.seed(5)
N=20
e=1
# Missing value percentages
PMD = 5
# Alpha, R_CRQ1,R_CRQ3
Alpha_R_CRQ=0.9
# e, R_MRQ1,R_MRQ3
e1_R_MRQ=0.9
e2_R_MRQ=1-e1_R_MRQ
# Alpha, R_MCRQ1,R_MCRQ3
Alpha_R_MCRQ=0.1
W1_R_MCRQ=0.1
W2_R_MCRQ=1-W1_R_MCRQ


m=1000
sum_mse_REG <- 0
sum_mse_RRQ1 <- 0
sum_mse_RRQ3 <- 0
sum_mse_CRQ1 <- 0
sum_mse_CRQ3 <- 0
sum_mse_MRQ1 <- 0
sum_mse_MRQ3 <- 0
sum_mse_MCRQ1 <- 0
sum_mse_MCRQ3 <- 0
```

```
sum_mape_REG <- 0
sum_mape_RRQ1 <- 0
sum_mape_RRQ3 <- 0
sum_mape_CRQ1 <- 0
sum_mape_CRQ3 <- 0
sum_mape_MRQ1 <- 0
sum_mape_MRQ3 <- 0
sum_mape_MCRQ1 <- 0
sum_mape_MCRQ3 <- 0


for (j in 1:m) {

  # Construct population X1
  Data_random_X1_RRSS = rnorm(100000,3,sqrt(1.5))
  X1 = Data_random_X1_RRSS
  print(Data_random_X1_RRSS)
  # Construct population X2
  Data_random_X2_RRSS = rnorm(100000,5,sqrt(2))
  X2 = Data_random_X2_RRSS
  print(Data_random_X2_RRSS)
  # Construct population E
  Data_e = sqrt(e)
  E = rnorm(100000,mean=0,sd=Data_e)
  print(E)
```

```
# Random variable X1
Data_RRSS_X1 =rep(0,times =N)
for (j in 1:N) {
  Sample_data_X1_RRSS = sample(Data_random_X1_RRSS, size=N,)
  Data_X1_sort_RRSS = sort(Sample_data_X1_RRSS)
  print(Data_X1_sort_RRSS)
  New_Estimate_Data_X1_sort_RRSS = Data_X1_sort_RRSS[j]
  print(New_Estimate_Data_X1_sort_RRSS)
  Data_RRSS_X1=replace(Data_RRSS_X1,list= j,values = New_Estimate_Data_X1_sort_RRSS)
  Data_RRSS_X1
}
print(Data_RRSS_X1)
Data_X1=Data_RRSS_X1
# Random variable X2
Data_RRSS_X2 =rep(0,times =N)
for (j in 1:N) {
  Sample_data_X2_RRSS = sample(Data_random_X2_RRSS, size=N,)
  Data_X2_sort_RRSS = sort(Sample_data_X2_RRSS)
  print(Data_X2_sort_RRSS)
  New_Estimate_Data_X2_sort_RRSS = Data_X2_sort_RRSS[j]
  print(New_Estimate_Data_X2_sort_RRSS)
  Data_RRSS_X2=replace(Data_RRSS_X2,list= j,values = New_Estimate_Data_X2_sort_RRSS)
  Data_RRSS_X2
}
print(Data_RRSS_X2)
Data_X2=Data_RRSS_X2
# Random variable E
Sample_E = sample(E, size=N,)
print(Sample_E)
```

```
Data_E=Sample_E

# Correlation
X_correlation = cbind(Data_X1,Data_X2)
cor(X_correlation)

# Construct variable Y
Y = function(Data_X1,Data_X2,Data_e) {
  0.5+(1*(Data_X1))+(-0.3*(Data_X2)+Data_e)
}
Y(Data_X1,Data_X2,Data_e)

# Calculate count of missing value
NMD = function(N,PMD) {
  (N)*(PMD)/100
}
NMD(N,PMD)# Count of missing value

# Calculate position of missing value
N_MD = NMD(N,PMD)
PM=sample(N,size = N_MD)
PM

# Estimate missing value_RRQ1
Data_Xi_REG=rep(1,N)
Data_Xi_REG
Data_Y_REG = Y(Data_X1,Data_X2,Data_e)
Data_X_REG = c(Data_Xi_REG, Data_X1, Data_X2)
matrix_X_REG <- matrix(Data_X_REG,nrow = N,ncol = 3)
```

```
matrix_X_REG

matrix_Y_REG <- matrix(Data_Y_REG,nrow = N,ncol = 1)

matrix_Y_REG

# Estimate missing value

New_Estimate_REG = (sum(Data_X2)-(matrix_X_REG[PM,3]))/(N-N_MD)

New_Estimate_REG

Data_X2_REG_New=replace(Data_X2,list= PM,values = New_Estimate_REG)

Data_X2_REG_New

# Construct matrix after estimate missing value

Data_X_REG_New = c(Data_Xi_REG, Data_X1, Data_X2_REG_New)

matrix_X_REG_New <- matrix(Data_X_REG_New,nrow = N,ncol = 3)

matrix_X_REG_New

# X'_REG_New

Transpose_X_REG_New = t(matrix_X_REG_New)

Transpose_X_REG_New

# X'X_REG_New

TransposeX_X_REG_New = Transpose_X_REG_New%*%matrix_X_REG_New

TransposeX_X_REG_New

# X'Y_REG_New

TransposeX_Y_REG_New = Transpose_X_REG_New%*%matrix_Y_REG

TransposeX_Y_REG_New

# Inverse(X'X)_REG_New

Inverse_TransposeX_X_REG_New = solve(TransposeX_X_REG_New)

Inverse_TransposeX_X_REG_New

# B_REG_New

B_REG_New = Inverse_TransposeX_X_REG_New%*%TransposeX_Y_REG_New

B_REG_New

# Equation model after estimate missing value

cat("Yhat=",B_REG_New[1,1],"+",B_REG_New[2,1],"X1+",B_REG_New[3,1],"X2+e")
```

```
# Y after estimate missing value
Y_REG_New = function(B_REG_New,Data_X1,Data_X2_REG_New,Data_E) {
  B_REG_New[1,1]+(B_REG_New[2,1]*Data_X1)+(B_REG_New[3,1]*Data_X2_REG_New)+Data_E
}
Y_REG_New(B_REG_New,Data_X1,Data_X2_REG_New,Data_E)
matrix_Y_REG_New <- matrix(Y_REG_New(B_REG_New,Data_X1,Data_X2_REG_New,Data_E),nrow =
N,ncol = 1)
matrix_Y_REG_New
Data_Y_REG_New=Y_REG_New(B_REG_New,Data_X1,Data_X2_REG_New,Data_E)
matrix_Y_REG_New <- matrix(Data_Y_REG_New,nrow = N,ncol = 1)
matrix_Y_REG_New
# Position of Q1
R1=1
RRQ1=function(N,R1) {
  (R1*(N+1))/4
}
RRQ1(N,R1)
# Data position of Q1
P_RRQ1=RRQ1(N,R1)
Data_X2_Sort=sort(Data_X2)
Data_X2_Sort
matrix_Data_X2_Sort <- matrix(Data_X2_Sort,nrow = N,ncol = 1)
matrix_Data_X2_Sort
Data_X2_RRQ1 = matrix_Data_X2_Sort[P_RRQ1,1]
Data_X2_RRQ1
New_Estimate_RRQ1 =
(matrix_Y_REG_New[PM,1])*((mean(Data_X2)+Data_X2_RRQ1)/(((sum(Data_X2)-
(matrix_X_REG[PM,3]))/(N-N_MD))+Data_X2_RRQ1))
New_Estimate_RRQ1
Data_Y_RRQ1_New=replace(Data_Y_REG_New,list= PM,values = New_Estimate_RRQ1)
```

```
Data_Y_RRQ1_New

# MSE_RRQ1

matrix_Y_RRQ1_New <- matrix(Data_Y_RRQ1_New,nrow = N,ncol = 1)

matrix_Y_RRQ1_New

MSE_RRQ1 = function(N,matrix_Y_REG,matrix_Y_RRQ1_New) {

  (1/N)*(sum((matrix_Y_REG-matrix_Y_RRQ1_New)^2))

}

MSE_RRQ1(N,matrix_Y_REG,matrix_Y_RRQ1_New)

# MAPE_RRQ1

MAPE_RRQ1 = function(N,matrix_Y_REG,matrix_Y_RRQ1_New) {

  (1/N)*(sum((abs(matrix_Y_REG-matrix_Y_RRQ1_New)/abs(matrix_Y_REG))*100))

}

MAPE_RRQ1(N,matrix_Y_REG,matrix_Y_RRQ1_New)
```

```
# Estimate missing value_RRQ3
Data_Y_REG_New=Y_REG_New(B_REG_New,Data_X1,Data_X2_REG_New,Data_E)
matrix_Y_REG_New <- matrix(Data_Y_REG_New,nrow = N,ncol = 1)
matrix_Y_REG_New
# Position of Q3
R3=3
RRQ3=function(N,R3) {
  (R3*(N+1))/4
}
RRQ3(N,R3)
# Data position of Q3
P_RRQ3=RRQ3(N,R3)
Data_X2_Sort=sort(Data_X2)
Data_X2_Sort
matrix_X2_Sort <- matrix(Data_X2_Sort,nrow = N,ncol = 1)
matrix_X2_Sort
Data_X2_RRQ3 = matrix_X2_Sort[P_RRQ3,1]
Data_X2_RRQ3
New_Estimate_RRQ3 =
(matrix_Y_REG_New[PM,1])*((mean(Data_X2)+Data_X2_RRQ3)/(((sum(Data_X2)-
(matrix_X_REG[PM,3]))/(N-N_MD))+Data_X2_RRQ3))
New_Estimate_RRQ3
Data_Y_RRQ3_New=replace(Data_Y_REG_New,list= PM,values = New_Estimate_RRQ3)
Data_Y_RRQ3_New
# MSE_RRQ3
matrix_Y_RRQ3_New <- matrix(Data_Y_RRQ3_New,nrow = N,ncol = 1)
matrix_Y_RRQ3_New
MSE_RRQ3 = function(N,matrix_Y_REG,matrix_Y_RRQ3_New) {
```

```
  (1/N)*(sum((matrix_Y_REG-matrix_Y_RRQ3_New)^2))
}
MSE_RRQ3(N,matrix_Y_REG,matrix_Y_RRQ3_New)
# MAPE_RRQ3
MAPE_RRQ3 = function(N,matrix_Y_REG,matrix_Y_RRQ3_New) {
  (1/N)*(sum((abs(matrix_Y_REG-matrix_Y_RRQ3_New)/abs(matrix_Y_REG))*100))
}
MAPE_RRQ3(N,matrix_Y_REG,matrix_Y_RRQ3_New)
```

```
# Estimate missing value_R_CRQ1
Data_Y_REG_New=Y_REG_New(B_REG_New,Data_X1,Data_X2_REG_New,Data_E)
matrix_Y_REG_New <- matrix(Data_Y_REG_New,nrow = N,ncol = 1)
matrix_Y_REG_New
# Position of Q1
R1=1
RRQ1=function(N,R1) {
  (R1*(N+1))/4
}
RRQ1(N,R1)
# Data position of Q1
P_RRQ1=RRQ1(N,R1)
Data_X2_Sort=sort(Data_X2)
Data_X2_Sort
matrix_Data_X2_Sort <- matrix(Data_X2_Sort,nrow = N,ncol = 1)
matrix_Data_X2_Sort
Data_X2_RRQ1 = matrix_Data_X2_Sort[P_RRQ1,1]
Data_X2_RRQ1
New_Estimate_R_CRQ1 =
(matrix_Y_REG_New[PM,1])*(((mean(Data_X2)+Data_X2_RRQ1)/(((sum(Data_X2)-
(matrix_X_REG[PM,3]))/(N-N_MD))+Data_X2_RRQ1))^Alpha_R_CRQ)
New_Estimate_R_CRQ1
Data_Y_R_CRQ1_New=replace(Data_Y_REG,list= PM,values = New_Estimate_R_CRQ1)
Data_Y_R_CRQ1_New
# MSE_R_CRQ1
matrix_Y_R_CRQ1_New <- matrix(Data_Y_R_CRQ1_New,nrow = N,ncol = 1)
matrix_Y_R_CRQ1_New
MSE_R_CRQ1 = function(N,matrix_Y_REG,matrix_Y_R_CRQ1_New) {
```

```
  (1/N)*(sum((matrix_Y_REG-matrix_Y_R_CRQ1_New)^2))
}
MSE_R_CRQ1(N,matrix_Y_REG,matrix_Y_R_CRQ1_New)
# MAPE_CRQ1
MAPE_R_CRQ1 = function(N,matrix_Y_REG,matrix_Y_R_CRQ1_New) {
  (1/N)*(sum((abs(matrix_Y_REG-matrix_Y_R_CRQ1_New)/abs(matrix_Y_REG))*100))
}
MAPE_R_CRQ1(N,matrix_Y_REG,matrix_Y_R_CRQ1_New)
```

```
# Estimate missing value_R_CRQ3
Data_Y_REG_New=Y_REG_New(B_REG_New,Data_X1,Data_X2_REG_New,Data_E)
matrix_Y_REG_New <- matrix(Data_Y_REG_New,nrow = N,ncol = 1)
matrix_Y_REG_New
# Position of Q3
R3=3
RRQ3=function(N,R3) {
  (R3*(N+1))/4
}
RRQ3(N,R3)
# Data position of Q3
P_RRQ3=RRQ3(N,R3)
Data_X2_Sort=sort(Data_X2)
Data_X2_Sort
matrix_X2_Sort <- matrix(Data_X2_Sort,nrow = N,ncol = 1)
matrix_X2_Sort
Data_X2_RRQ3 = matrix_X2_Sort[P_RRQ3,1]
Data_X2_RRQ3
New_Estimate_R_CRQ3 =
(matrix_Y_REG_New[PM,1])*(((mean(Data_X2)+Data_X2_RRQ3)/(((sum(Data_X2)-
(matrix_X_REG[PM,3]))/(N-N_MD))+Data_X2_RRQ3))^Alpha_R_CRQ)
New_Estimate_R_CRQ3
Data_Y_R_CRQ3_New=replace(Data_Y_REG,list= PM,values = New_Estimate_R_CRQ3)
Data_Y_R_CRQ3_New
# MSE_R_CRQ3
matrix_Y_R_CRQ3_New <- matrix(Data_Y_R_CRQ3_New,nrow = N,ncol = 1)
matrix_Y_R_CRQ3_New
MSE_R_CRQ3 = function(N,matrix_Y_REG,matrix_Y_R_CRQ3_New) {
```

```
  (1/N)*(sum((matrix_Y_REG-matrix_Y_R_CRQ3_New)^2))
}
MSE_R_CRQ3(N,matrix_Y_REG,matrix_Y_R_CRQ3_New)
# MAPE_CRQ3
MAPE_R_CRQ3 = function(N,matrix_Y_REG,matrix_Y_R_CRQ3_New) {
  (1/N)*(sum((abs(matrix_Y_REG-matrix_Y_R_CRQ3_New)/abs(matrix_Y_REG))*100))
}
MAPE_R_CRQ3(N,matrix_Y_REG,matrix_Y_R_CRQ3_New)
```

```
# Estimate missing value_R_MRQ1

Data_Y_REG_New=Y_REG_New(B_REG_New,Data_X1,Data_X2_REG_New,Data_E)

matrix_Y_REG_New <- matrix(Data_Y_REG_New,nrow = N,ncol = 1)

matrix_Y_REG_New

# Position of Q1

R1=1

RRQ1=function(N,R1) {

  (R1*(N+1))/4

}

RRQ1(N,R1)

# Data position of Q1

P_RRQ1=RRQ1(N,R1)

Data_X1_Sort=sort(Data_X1)

Data_X1_Sort

matrix_Data_X1_Sort <- matrix(Data_X1_Sort,nrow = N,ncol = 1)

Data_X1_RRQ1 = matrix_Data_X1_Sort[P_RRQ1,1]

Data_X1_RRQ1

Data_X2_Sort=sort(Data_X2)

Data_X2_Sort

matrix_Data_X2_Sort <- matrix(Data_X2_Sort,nrow = N,ncol = 1)

matrix_Data_X2_Sort

Data_X2_RRQ1 = matrix_Data_X2_Sort[P_RRQ1,1]

Data_X2_RRQ1

New_Estimate_R_MRQ1 =
(matrix_Y_REG_New[PM,1])*((e2_R_MRQ*((mean(X2)+Data_X2_RRQ1)/(((sum(X2)-
(matrix_X_REG[PM,3]))/(N-
N_MD))+Data_X2_RRQ1)))+(e1_R_MRQ*((mean(X1)+Data_X1_RRQ1)/((mean(X1)+Data_X1_RRQ1)))))

Data_Y_R_MRQ1_New=replace(Data_Y_REG,list= PM,values = New_Estimate_R_MRQ1)

Data_Y_R_MRQ1_New
```

```
# MSE_R_MRQ1
matrix_Y_R_MRQ1_New <- matrix(Data_Y_R_MRQ1_New,nrow = N,ncol = 1)
matrix_Y_R_MRQ1_New
MSE_R_MRQ1 = function(N,matrix_Y_REG,matrix_Y_R_MRQ1_New) {
  (1/N)*(sum((matrix_Y_REG-matrix_Y_R_MRQ1_New)^2))
}
MSE_R_MRQ1(N,matrix_Y_REG,matrix_Y_R_MRQ1_New)
# MAPE_MRQ1
MAPE_R_MRQ1 = function(N,matrix_Y_REG,matrix_Y_R_MRQ1_New) {
  (1/N)*(sum((abs(matrix_Y_REG-matrix_Y_R_MRQ1_New)/abs(matrix_Y_REG))*100))
}
MAPE_R_MRQ1(N,matrix_Y_REG,matrix_Y_R_MRQ1_New)
```

```
# Estimate missing value_R_MRQ3
Data_Y_REG_New=Y_REG_New(B_REG_New,Data_X1,Data_X2_REG_New,Data_E)
matrix_Y_REG_New <- matrix(Data_Y_REG_New,nrow = N,ncol = 1)
matrix_Y_REG_New
# Position of Q3
R3=3
RRQ3=function(N,R3) {
  (R3*(N+1))/4
}
RRQ3(N,R3)
# Data position of Q3
P_RRQ3=RRQ3(N,R3)
Data_X1_Sort=sort(Data_X1)
Data_X1_Sort
matrix_Data_X1_Sort <- matrix(Data_X1_Sort,nrow = N,ncol = 1)
Data_X1_RRQ3 = matrix_Data_X1_Sort[P_RRQ3,1]
Data_X1_RRQ3
Data_X2_Sort=sort(Data_X2)
Data_X2_Sort
matrix_Data_X2_Sort <- matrix(Data_X2_Sort,nrow = N,ncol = 1)
matrix_Data_X2_Sort
Data_X2_RRQ3 = matrix_Data_X2_Sort[P_RRQ3,1]
Data_X2_RRQ3
New_Estimate_R_MRQ3 =
(matrix_Y_REG_New[PM,1])*((e2_R_MRQ*((mean(X2)+Data_X2_RRQ3)/(((sum(X2)-
(matrix_X_REG[PM,3]))/(N-
N_MD))+Data_X2_RRQ3)))+(e1_R_MRQ*((mean(X1)+Data_X1_RRQ3)/((mean(X1)+Data_X1_RRQ3)))))
Data_Y_R_MRQ3_New=replace(Data_Y_REG,list= PM,values = New_Estimate_R_MRQ3)
Data_Y_R_MRQ3_New
```

```
# MSE_R_MRQ3
matrix_Y_R_MRQ3_New <- matrix(Data_Y_R_MRQ3_New,nrow = N,ncol = 1)
matrix_Y_R_MRQ3_New
MSE_R_MRQ3 = function(N,matrix_Y_REG,matrix_Y_R_MRQ3_New) {
  (1/N)*(sum((matrix_Y_REG-matrix_Y_R_MRQ3_New)^2))
}
MSE_R_MRQ3(N,matrix_Y_REG,matrix_Y_R_MRQ3_New)
# MAPE_MRQ3
MAPE_R_MRQ3 = function(N,matrix_Y_REG,matrix_Y_R_MRQ3_New) {
  (1/N)*(sum((abs(matrix_Y_REG-matrix_Y_R_MRQ3_New)/abs(matrix_Y_REG))*100))
}
MAPE_R_MRQ3(N,matrix_Y_REG,matrix_Y_R_MRQ3_New)
```

```
# Estimate missing value_R_MCRQ1

Data_Y_REG_New=Y_REG_New(B_REG_New,Data_X1,Data_X2_REG_New,Data_E)

matrix_Y_REG_New <- matrix(Data_Y_REG_New,nrow = N,ncol = 1)

matrix_Y_REG_New

# Position of Q1

R1=1

RRQ1=function(N,R1) {

  (R1*(N+1))/4

}

RRQ1(N,R1)

# Data position of Q1

P_RRQ1=RRQ1(N,R1)

Data_X1_Sort=sort(Data_X1)

Data_X1_Sort

matrix_Data_X1_Sort <- matrix(Data_X1_Sort,nrow = N,ncol = 1)

Data_X1_RRQ1 = matrix_Data_X1_Sort[P_RRQ1,1]

Data_X1_RRQ1

Data_X2_Sort=sort(Data_X2)

Data_X2_Sort

matrix_Data_X2_Sort <- matrix(Data_X2_Sort,nrow = N,ncol = 1)

matrix_Data_X2_Sort

Data_X2_RRQ1 = matrix_Data_X2_Sort[P_RRQ1,1]

Data_X2_RRQ1

New_Estimate_R_MCRQ1 =
(matrix_Y_REG_New[PM,1])*((((W2_R_MCRQ*(mean(X2)+Data_X2_RRQ1))+(W1_R_MCRQ*((mean(X1)
+Data_X1_RRQ1))))/((W2_R_MCRQ*(sum(X2)-
(matrix_X_REG[PM,3])))+(W1_R_MCRQ*(mean(X1)+Data_X1_RRQ1))))^Alpha_R_MCRQ)

Data_Y_R_MCRQ1_New=replace(Data_Y_REG,list= PM,values = New_Estimate_R_MCRQ1)

Data_Y_R_MCRQ1_New
```

```
# MSE_R_MCRQ1
matrix_Y_R_MCRQ1_New <- matrix(Data_Y_R_MCRQ1_New,nrow = N,ncol = 1)
matrix_Y_R_MCRQ1_New
MSE_R_MCRQ1 = function(N,matrix_Y_REG,matrix_Y_R_MCRQ1_New) {
  (1/N)*(sum((matrix_Y_REG-matrix_Y_R_MCRQ1_New)^2))
}
MSE_R_MCRQ1(N,matrix_Y_REG,matrix_Y_R_MCRQ1_New)
# MAPE_MCRQ1
MAPE_R_MCRQ1 = function(N,matrix_Y_REG,matrix_Y_R_MCRQ1_New) {
  (1/N)*(sum((abs(matrix_Y_REG-matrix_Y_R_MCRQ1_New)/abs(matrix_Y_REG))*100))
}
MAPE_R_MCRQ1(N,matrix_Y_REG,matrix_Y_R_MCRQ1_New)
```

```
  # Estimate missing value_R_MCRQ3
 Data_Y_REG_New=Y_REG_New(B_REG_New,Data_X1,Data_X2_REG_New,Data_E)
 matrix_Y_REG_New <- matrix(Data_Y_REG_New,nrow = N,ncol = 1)
 matrix_Y_REG_New
 # Position of Q3
 R3=3
 RRQ3=function(N,R3) {
  (R3*(N+1))/4
 }
 RRQ3(N,R3)
 # Data position of Q3
 P_RRQ3=RRQ3(N,R3)
 Data_X1_Sort=sort(Data_X1)
 Data_X1_Sort
 matrix_Data_X1_Sort <- matrix(Data_X1_Sort,nrow = N,ncol = 1)
 Data_X1_RRQ3 = matrix_Data_X1_Sort[P_RRQ3,1]
 Data_X1_RRQ3
 Data_X2_Sort=sort(Data_X2)
 Data_X2_Sort
 matrix_Data_X2_Sort <- matrix(Data_X2_Sort,nrow = N,ncol = 1)
 matrix_Data_X2_Sort
 Data_X2_RRQ3 = matrix_Data_X2_Sort[P_RRQ3,1]
 Data_X2_RRQ3
 New_Estimate_R_MCRQ3
=(matrix_Y_REG_New[PM,1])*((((W2_R_MCRQ*(mean(X2)+Data_X2_RRQ3))+(W1_R_MCRQ*((mean(X1
)+Data_X1_RRQ3))))/((W2_R_MCRQ*(sum(X2)-
(matrix_X_REG[PM,3])))+(W1_R_MCRQ*(mean(X1)+Data_X1_RRQ3))))^Alpha_R_MCRQ)
 Data_Y_R_MCRQ3_New=replace(Data_Y_REG,list= PM,values = New_Estimate_R_MCRQ3)
 Data_Y_R_MCRQ3_New
```

```
# MSE_R_MCRQ3
matrix_Y_R_MCRQ3_New <- matrix(Data_Y_R_MCRQ3_New,nrow = N,ncol = 1)
matrix_Y_R_MCRQ3_New
MSE_R_MCRQ3 = function(N,matrix_Y_REG,matrix_Y_R_MCRQ3_New) {
  (1/N)*(sum((matrix_Y_REG-matrix_Y_R_MCRQ3_New)^2))
}
MSE_R_MCRQ3(N,matrix_Y_REG,matrix_Y_R_MCRQ3_New)
# MAPE_MCRQ3
MAPE_R_MCRQ3 = function(N,matrix_Y_REG,matrix_Y_R_MCRQ3_New) {
  (1/N)*(sum((abs(matrix_Y_REG-matrix_Y_R_MCRQ3_New)/abs(matrix_Y_REG))*100))
}
MAPE_R_MCRQ3(N,matrix_Y_REG,matrix_Y_R_MCRQ3_New)
```

```
RRQ1_MSE=MSE_RRQ1(N,matrix_Y_REG,matrix_Y_RRQ1_New)
sum_mse_RRQ1 = sum_mse_RRQ1+RRQ1_MSE
RRQ3_MSE=MSE_RRQ3(N,matrix_Y_REG,matrix_Y_RRQ3_New)
sum_mse_RRQ3 = sum_mse_RRQ3+RRQ3_MSE
CRQ1_MSE=MSE_R_CRQ1(N,matrix_Y_REG,matrix_Y_R_CRQ1_New)
sum_mse_CRQ1 = sum_mse_CRQ1+CRQ1_MSE
CRQ3_MSE=MSE_R_CRQ3(N,matrix_Y_REG,matrix_Y_R_CRQ3_New)
sum_mse_CRQ3 = sum_mse_CRQ3+CRQ3_MSE
MRQ1_MSE=MSE_R_MRQ1(N,matrix_Y_REG,matrix_Y_R_MRQ1_New)
sum_mse_MRQ1 = sum_mse_MRQ1+MRQ1_MSE
MRQ3_MSE=MSE_R_MRQ3(N,matrix_Y_REG,matrix_Y_R_MRQ3_New)
sum_mse_MRQ3 = sum_mse_MRQ3+MRQ3_MSE
MCRQ1_MSE=MSE_R_MCRQ1(N,matrix_Y_REG,matrix_Y_R_MCRQ1_New)
sum_mse_MCRQ1 = sum_mse_MCRQ1+MCRQ1_MSE
MCRQ3_MSE=MSE_R_MCRQ3(N,matrix_Y_REG,matrix_Y_R_MCRQ3_New)
sum_mse_MCRQ3 = sum_mse_MCRQ3+MCRQ3_MSE

RRQ1_MAPE=MAPE_RRQ1(N,matrix_Y_REG,matrix_Y_RRQ1_New)
sum_mape_RRQ1 = sum_mape_RRQ1+RRQ1_MAPE
RRQ3_MAPE=MAPE_RRQ3(N,matrix_Y_REG,matrix_Y_RRQ3_New)
sum_mape_RRQ3 = sum_mape_RRQ3+RRQ3_MAPE
CRQ1_MAPE=MAPE_R_CRQ1(N,matrix_Y_REG,matrix_Y_R_CRQ1_New)
sum_mape_CRQ1 = sum_mape_CRQ1+CRQ1_MAPE
CRQ3_MAPE=MAPE_R_CRQ3(N,matrix_Y_REG,matrix_Y_R_CRQ3_New)
sum_mape_CRQ3 = sum_mape_CRQ3+CRQ3_MAPE
MRQ1_MAPE=MAPE_R_MRQ1(N,matrix_Y_REG,matrix_Y_R_MRQ1_New)
sum_mape_MRQ1 = sum_mape_MRQ1+MRQ1_MAPE
MRQ3_MAPE=MAPE_R_MRQ3(N,matrix_Y_REG,matrix_Y_R_MRQ3_New)
```

```
  sum_mape_MRQ3 = sum_mape_MRQ3+MRQ3_MAPE
  MCRQ1_MAPE=MAPE_R_MCRQ1(N,matrix_Y_REG,matrix_Y_R_MCRQ1_New)
  sum_mape_MCRQ1 = sum_mape_MCRQ1+MCRQ1_MAPE
  MCRQ3_MAPE=MAPE_R_MCRQ3(N,matrix_Y_REG,matrix_Y_R_MCRQ3_New)
  sum_mape_MCRQ3 = sum_mape_MCRQ3+MCRQ3_MAPE

  cat(c("Finish"),fill=T)
}


AVR_MSE_RRQ1 = sum_mse_RRQ1/m
AVR_MSE_RRQ1
AVR_MSE_RRQ3 = sum_mse_RRQ3/m
AVR_MSE_RRQ3
AVR_MSE_CRQ1 = sum_mse_CRQ1/m
AVR_MSE_CRQ1
AVR_MSE_CRQ3 = sum_mse_CRQ3/m
AVR_MSE_CRQ3
AVR_MSE_MRQ1 = sum_mse_MRQ1/m
AVR_MSE_MRQ1
AVR_MSE_MRQ3 = sum_mse_MRQ3/m
AVR_MSE_MRQ3
AVR_MSE_MCRQ1 = sum_mse_MCRQ1/m
AVR_MSE_MCRQ1
AVR_MSE_MCRQ3 = sum_mse_MCRQ3/m
AVR_MSE_MCRQ3

AVR_MAPE_RRQ1 = sum_mape_RRQ1/m
AVR_MAPE_RRQ1
```

AVR_MAPE_RRQ3 = sum_mape_RRQ3/m

AVR_MAPE_RRQ3

AVR_MAPE_CRQ1 = sum_mape_CRQ1/m

AVR_MAPE_CRQ1

AVR_MAPE_CRQ3 = sum_mape_CRQ3/m

AVR_MAPE_CRQ3

AVR_MAPE_MRQ1 = sum_mape_MRQ1/m

AVR_MAPE_MRQ1

AVR_MAPE_MRQ3 = sum_mape_MRQ3/m

AVR_MAPE_MRQ3

AVR_MAPE_MCRQ1 = sum_mape_MCRQ1/m

AVR_MAPE_MCRQ1

AVR_MAPE_MCRQ3 = sum_mape_MCRQ3/m

AVR_MAPE_MCRQ3
