# Supplementary material for: New adjusted missing value imputation in multiple regression with simple random sampling and rank set sampling methods
Source: PLoS One. 2025 Mar 17;20(3):e0316641. doi: 10.1371/journal.pone.0316641 (PMC11913305; doi:10.1371/journal.pone.0316641)
Supplement: Actual Data — (PDF) [file pone.0316641.s003.pdf]

Table 1 Data of gold loss by customer groups

Department	Month	Y (Loss of precious metals)	X_1 (Input weight)	X_2 (Output weight)	X_3 (Refinement)
**Bridal customer group**	January	31.9991	2,313.6079	2,141.5979	140.0109
	February	59.1330	3,596.4387	3,416.0293	121.2764
	March	48.8532	4,956.4950	4,747.2250	160.4168
	April	34.8258	2,810.1394	2,625.4589	149.8547
	May	35.9909	2,102.0168	1,949.9149	116.1110
	June	10.8303	957.2834	883.6118	62.8413
	July	23.0665	2,130.4516	2,005.2223	102.1628
	August	28.5572	3,038.9862	2,883.9485	126.4805
	September	9.7517	885.2416	832.2991	43.1908
	October	48.2928	2,151.6729	1,950.3288	153.0513
	November	60.3569	3,236.0400	3,062.5125	113.1706
	December	32.3371	1,765.6950	1,672.7250	60.6329
**Japanese customer group**	January	413.8726	20,570.5734	18,239.1546	1,917.5462
	February	492.6038	23,457.1359	21,093.8922	1,870.6399
	March	452.1213	21,097.6234	19,016.1061	1,629.3960
	April	389.5646	13,889.7610	12,154.2168	1,345.9796
	May	374.5406	15,190.6709	13,722.5677	1,093.5626
	June	414.2982	20,330.7724	18,747.2789	1,169.1953
	July	457.1055	20,262.5734	18,401.7803	1,403.6876
	August	461.5860	22,046.2179	20,381.7676	1,202.8643
	September	445.1815	22,741.6081	20,777.2755	1,519.1511
	October	533.2217	24,938.7197	22,637.1281	1,768.3699
	November	524.2128	32,363.1792	29,793.0022	2,045.9642
	December	426.9813	14,569.2654	12,890.9547	1,251.3294
**Asia customer group**	January	1,791.8684	70,518.7950	67268.6775	1,458.2491
	February	611.0320	45,711.0450	44,628.4200	471.5930
	March	688.1363	39,595.0022	38,030.1991	876.6668
	April	448.1742	38,504.9081	37,492.8901	563.8438
	May	141.2387	19,904.9114	19,595.5914	168.0813
	June	359.9342	11,657.1450	11,120.5950	176.6158
	July	1,193.1503	46,895.9400	45,020.8598	681.9299
	August	378.2322	19,833.3269	19,243.3728	211.7219
	September	1,008.1962	37,000.4798	35,201.9498	790.3338
	October	997.1261	53,654.5200	51,951.5100	705.8839
	November	1,404.7811	74,847.4275	72,710.4938	732.1526
	December	806.6078	60,312.3354	58,672.6080	833.1196
**European customer group**	January	473.8005	163,14.1352	15,506.4217	333.9130
	February	156.3300	15,309.2745	14,608.8700	544.0745
	March	459.8749	17,987.1969	17,141.7437	385.5783
	April	150.2493	12,033.3303	11,405.8509	477.2301
	May	125.9122	11,961.4323	11,354.4523	481.0678
	June	359.9342	11,657.1450	11,120.5950	176.6158
	July	279.9724	12,617.4449	11,951.6100	385.8625
	August	208.0532	10,639.4213	9,998.3886	432.9795
	September	255.0002	13,363.5269	12,680.5260	428.0007
	October	311.0871	13,443.5001	12,528.0688	604.3442
	November	54.0272	12,984.6870	12,301.6690	628.9908
	December	213.5022	12,138.2205	11,566.1265	358.5918

Table 2 Data of platinum loss by customer groups

Department	Month	Y (Loss of precious metals)	X_1 (Input weight)	X_2 (Output weight)	X_3 (Refinement)
**Bridal customer group**	January	295.7914	22,894.6500	21,389.9500	1,208.9086
	February	276.8282	22,501.6935	21,142.7295	1,082.1358
	March	388.7447	23,319.0355	21,868.4905	1,061.8003
	April	339.3845	19,875.5050	18,461.9195	1,074.2010
	May	348.7388	25,100.9520	23,648.4045	1,103.8087
	June	286.2889	19,939.3825	18,746.9480	906.1456
	July	642.5793	24,791.5090	23,358.4375	790.4922
	August	477.2729	21,409.7440	20,271.0620	661.4091
	September	639.1203	26,001.8415	24,477.0225	885.6987
	October	227.7606	13,439.0525	12,279.2835	932.0084
	November	390.7633	27,772.5580	26,378.5410	1003.2537
	December	486.7429	24,256.1125	23,074.8485	694.5211
**Japanese customer group**	January	235.1743	27,735.2405	26,944.0160	556.0502
	February	277.2673	31,527.0565	30,532.8945	716.8947
	March	228.1340	32,437.8360	31,447.7792	761.9228
	April	257.5829	30,085.8290	28,975.4735	852.7726
	May	295.5906	33,157.9475	32,104.3960	757.9609
	June	190.4972	19,636.1530	19,043.6940	401.9618
	July	284.2365	30,087.9454	29,172.9495	630.7594
	August	253.8689	31,633.9490	30,673.6912	706.3889
	September	202.8494	24,515.6410	23,755.5405	557.2511
	October	277.0950	32,949.0195	32,097.9555	573.9690
	November	230.4457	29,056.2979	28,381.2583	444.5939
	December	232.2373	23,877.6166	23,187.6543	457.7250
**Asia customer group**	January	2.4548	299.4240	295.2470	1.7222
	February	0.7240	15.9315	14.9815	0.2260
	March	0.5657	15.8080	14.9625	0.2798
	April	0.1666	35.7295	35.3970	0.1659
	May	0.2016	47.5570	46.9110	0.4444
	June	2.3265	85.4905	82.9065	0.2575
	July	0.6273	20.1875	19.3515	0.2087
	August	0.9027	23.1325	21.6220	0.6078
	September	2.0420	172.1305	164.0555	3.0330
	October	2.7907	1,215.0700	1,194.0147	15.2646
	November	2.1384	332.0725	322.4300	2.5041
	December	2.3492	649.5150	634.1690	9.9968
**European customer group**	January	5.3215	680.3830	628.2838	46.7777
	February	9.5382	1,020.3680	940.6335	70.1963
	March	9.1047	796.4610	743.2182	44.1381
	April	9.6821	929.7745	872.4184	47.6740
	May	5.7510	780.9855	711.2748	63.9597
	June	8.9559	860.8900	799.5649	52.3692
	July	19.7885	1,421.6959	1,302.4232	99.4842
	August	15.6694	1,321.3670	1,226.1832	79.5144
	September	2.6641	316.2585	295.4206	18.1738
	October	9.9505	762.0975	700.6780	51.4690
	November	8.3206	813.5240	747.0080	58.1954
	December	7.3963	813.7985	775.7816	30.6206

The data are used for gold and platinum loss in the production process of a jewelry company in 4 departments: Bridal customer group, Japanese customer group, Asian customer group, and European customer group.

The independent variables consist of 3 variables: the input weight of all pieces (X1), the output weight of all pieces (X2), and the melt refinement (X3). The dependent variable is the loss of precious metal (Y). The missing value in the melt refinement variable (X3). The sample size used is 12 units.
