# Supplementary material for: New adjusted missing value imputation in multiple regression with simple random sampling and rank set sampling methods
Source: PLoS One. 2025 Mar 17;20(3):e0316641. doi: 10.1371/journal.pone.0316641 (PMC11913305; doi:10.1371/journal.pone.0316641)
Supplement: Attachment — Submitted filename: Response to Reviewers.pdf [file pone.0316641.s004.pdf]

Response to Academic Editor and Reviewers

New adjusted missing value imputation in multiple regression with simple random sampling and rank set sampling methods

Response to Academic Editor

Journal requirements:
When submitting your revision, we need you to address these additional requirements.
1. Please ensure that your manuscript meets PLOS ONE's style requirements, including those for file naming. The PLOS ONE style templates can be found at https://journals.plos.org/plosone/s/file?id=wjVg/PLOSOne_formatting_sample_main_body.pdf and https://journals.plos.org/plosone/s/file?id=ba62/PLOSOne_formatting_sample_title_authors_affiliations.pdf
https://journals.plos.org/plosone/s/file?id=wjVg/PLOSOne_formatting_sample_main_body.pdf and https://journals.plos.org/plosone/s/file?id=ba62/PLOSOne_formatting_sample_title_authors_affiliations.pdf

Response:

My manuscript meets PLOS ONE's style requirements, including those for file naming.

2. Please note that PLOS ONE has specific guidelines on code sharing for submissions in which author-generated code underpins the findings in the manuscript. In these cases, all author-generated code must be made available without restrictions upon publication of the work. Please review our guidelines at https://journals.plos.org/plosone/s/materials-and-software-sharing#loc-sharing-code and ensure that your code is shared in a way that follows best practice and facilitates reproducibility and reuse.

Sharing Code
We expect all researchers with submissions to PLOS in which author-generated code underpins the findings in the manuscript to make all author-generated code available without restrictions upon publication of the work. In cases where code is central to the manuscript, we may require the code to be made available as a condition of publication. Authors are responsible for ensuring that the code is reusable and well documented.

Recommendations for sharing code
Authors are encouraged to share their code in a way that follows best practice and facilitates reproducibility and reuse. Authors are encouraged to:
-Share their code via a repository that issues persistent identifiers, such as DOIs.
-Clearly license their code using an open source license.
-Share clear documentation alongside the code that details any information needed to run the code, for example dependencies.
-State how the code can be accessed in the Data Availability Statement of their manuscript.

More information on best practice for sharing code can be found in the PLOS Computational Biology author guidance.

If code cannot be shared due to legal or ethical reasons then authors should state this in the Data Availability Statement and give details of how to request access to the code. In cases where code can not be publicly shared, editors and reviewers reserve the right to access the code if deemed necessary for full assessment of the manuscript.

Response:

The data used to support this study were simulated from normal distribution using R studio program. The real-world data about gold and platinum losses were taken from an anonymous jewelry company. I share the simulation code in two attached files named "Simulation code1 SRS and Simulation code2 RSS".

3. Thank you for stating the following financial disclosure:
"School of Science, King Mongkut's Institute of Technology Ladkrabang (KMITL) for consideration of funding this research project [grant number 2567-02-5-001]"
Please state what role the funders took in the study. If the funders had no role, please state: "The funders had no role in study design, data collection and analysis, decision to publish, or preparation of the manuscript."
If this statement is not correct you must amend it as needed.
Please include this amended Role of Funder statement in your cover letter; we will change the online submission form on your behalf.

Response:

The funders had no role in the study design, data collection and analysis, decision to publish, or preparation of the manuscript.

4. Please update your submission to use the PLOS LaTeX template. The template and more information on our requirements for LaTeX submissions can be found at http://journals.plos.org/plosone/s/latex.

Response:

I have updated the manuscript into the PLOS LaTex template.

5. Thank you for stating the following in the Acknowledgments Section of your manuscript:
"The authors would like to thank the committees of the Department of Statistics and other Departments, School of Science, King Mongkut's Institute of Technology Ladkrabang (KMITL)
for consideration of funding this research project [grant number 2567-02-5-001]."
We note that you have provided funding information that is currently declared in your Funding Statement. However, funding information should not appear in the Acknowledgments section or other areas of your manuscript. We will only publish funding information present in the Funding Statement section of the online submission form.
Please remove any funding-related text from the manuscript and let us know how you would like to update your Funding Statement. Currently, your Funding Statement reads as follows:
"School of Science, King Mongkut's Institute of Technology Ladkrabang (KMITL) for consideration of funding this research project [grant number 2567-02-5-001]"
Please include your amended statements within your cover letter; we will change the online submission form on your behalf.

Response:

I have removed any funding-related text from the Acknowledgements section of my manuscript. There is no amended statement to state in the cover letter.

6. When completing the data availability statement of the submission form, you indicated that you will make your data available on acceptance. We strongly recommend all authors decide on a data sharing plan before acceptance, as the process can be lengthy and hold up publication timelines. Please note that, though access restrictions are acceptable now, your entire data will need to be made freely accessible if your manuscript is accepted for publication. This policy applies to all data except where public deposition would breach compliance with the protocol approved by your research ethics board. If you are unable to adhere to our open data policy, please kindly revise your statement to explain your reasoning and we will seek the editor's input on an exemption. Please be assured that, once you have provided your new statement, the assessment of your exemption will not hold up the peer review process.
[Note: HTML markup is below. Please do not edit.]

Response:

The data used to support this study were simulated from normal distribution using R studio program. I have made my raw simulation data available in two attached files in Microsoft Word to this manuscript. My raw simulation data available was included in simulation code. However, the real data on gold and platinum losses were taken from an anonymous jewelry company, whose identity is kept undisclosed.

Reviewers’ Comments to the Author and Responses

Reviewer #1: The manuscript investigates some imputation methods in multiple regression analysis for missing values. In essence, this work is only considered as a research report rather than a technique article. I cannot understand the specific contribution of this work. Meanwhile, the writing and organization are poor. Therefore, I cannot recommend this paper for publication in PLOS ONE

Response:

We have rewritten our contributions to the field and think that it is now clear.

We have extensively rewritten and reorganized the introduction section of the manuscript and added some information in the Materials and Methods; and Conclusions sections.

Reviewer #2: The manuscript focuses on the efficiency of four imputation methods: regression-ratio quartile1,3 (R-RQ1,3) imputation of Al-Omari, Jemain, and Ibrahim; adjusted regression-chain ratio quartile1,3 (AR-CRQ1,3) imputation of Kadilar and Cinji; adjusted regression-multivariate ratio quatile1,3 (ARMRQ1,3) imputation of Feng, Ni, and Zou; and adjusted regression-multivariate chain ratio quartile1,3 (AR-MCRQ1,3) imputation of Lu for each simple random sampling (SRS) and rank set sampling (RSS). Simulation studies were conducted to investigate the performance of different methods using mean square error and mean absolute error.

The manuscript addresses an interesting and important topic of missing imputation and is written clearly and concisely, but a major revision is needed, which should address the following concerns:

5.1) Literature review: The literature review is very selective, sorted by year of publication, and mainly includes older literature related to the approaches proposed by the authors. Important statistical literature on missing imputation in regression analysis (e.g. Song & Guo, 2024; Thongsri & Samart, 2022, see references) is not included, nor is included important basic literature to missing imputation, e.g. by Rubin or Little (see references). The question of alternatives to multiple imputations, e.g. maximum likelihood, is not discussed at all (e.g. Chen & Ibrahim, 2013).

The work of Al-Omari, Jemain, and Ibrahim only refers to SRS; but is not related to missing imputation. To contribute to the current literature on missing imputation in linear regression would also require a review of the entire literature on the subject.

Finally, the four proposed methods of missing imputation are new. They are mentioned in the introduction, but the more detailed explanations only follow in the methods section. Readers who are not familiar with SRS have no chance of understanding the idea of these new methods. It would be helpful to explain the basic idea of these procedures right in the beginning.

5.1.1) Important statistical literature on missing imputation in regression analysis (e.g. Song & Guo, 2024; Thongsri & Samart, 2022, see references) is not included, nor is included important basic literature to missing imputation, e.g. by Rubin or Little (see references). The question of alternatives to multiple imputation, e.g. maximum likelihood, is not discussed at all (e.g. Chen & Ibrahim, 2013).

Response:

Although our mentioning of papers is not intended to be a complete review of the entire literature on imputation methods, we have added the literature on missing imputation in regression analysis as suggested, including Song & Guo, 2024; Thongsri & Samart, 2022; Rubin, 1987; Little & Rubin, 1987; Little, 1992, 2021; and Chen & Ibrahim, 2013; in the Introduction section, so that they may be of benefits to some readers.

5.1.2) The work of Al-Omari, Jemain, and Ibrahim only refers to SRS; but is not related to missing imputation. To contribute to the current literature on missing imputation in linear regression would also require a review of the entire literature on the subject.

Response:

There were estimators for estimating the missing values in multiple regression analysis with simple random sampling (SRS). The R-RQ3 imputation of Al-Omari, Jemain, and Ibrahim was more efficient than an estimator based on the R-RQ1 imputation of Al-Omari, Jemain, and Ibrahim (Al-Omari AI et al., 2009). However, an efficient missing-value imputation method, the R-RQ1 imputation of Al-Omari, Jemain, and Ibrahim was more efficient in several situations. The R-RQ3 imputation of Al-Omari, Jemain, and Ibrahim was efficient for the situation of large sample size (n = 100), medium percentage of missing values (15%), and medium value of variance (σ^2 = 1) (Jomprapan, 2012).

5.1.3) Last but not least, the four proposed methods of missing imputation are new. They are mentioned in the introduction, but the more detailed explanations only follow in the methods section. Readers who are not familiar with SRS have no chance of understanding the idea of these new methods. It would be helpful to explain the basic idea of these procedures right in the beginning.

Response:

I added simple random sampling and rank set sampling ideas at the beginning Introduction and explained that SRS and RSS might yield a different data set and results from the new methods.

5.2) New methods of missing imputation: As these are new approaches developed by the authors themselves ("The authors propose three new methods"), a comparison with common missing imputation methods in linear regression, such as stochastic regression imputation, predictive mean matching imputation or random forest imputation (e.g., Thongsri & Samart, 2022) may be necessary.

Response:

A comparison of the proposed methods with the common imputation methods in multiple regression analysis was not done for several reasons. First, the stochastic regression imputation was not considered because, according to Isabella et al. (2022), the k-nearest stochastic regression imputation is less efficient than the regression-ratio quartile1 (RRQ1) and multiple imputation (MI). Second, the predictive mean matching imputation is less efficient than the K-Nearest Neighbor (KNN) and EM Algorithm (EM) (Warittha and Anupap, 2013). Finally, in this study, the small sample sizes were 20, 40, 60, 80, 100, and 120. The random forest imputation is a suitable machine learning method for large sample sizes, many records, and big data, which is different from the field of multiple regression analysis. Future recommendation in manuscript, the random forest imputation may be a good method for estimating missing value imputations in multiple regression analysis (Thongsri and Samart, 2022). A comparative study between this imputation method and the author’s method should be conducted.

Therefore, the stochastic regression imputation, predictive mean matching imputation, and random forest imputation were not considered in this research.

5.3) Assumptions: Three assumptions are discussed in the context of missing imputation, which also forms the basis of later simulations: "missing completely at random (MCAR)", "missing at random (MAR)" and "missing not at random (MNAR)" (Rubin, 1987; Von Buuren, 2018). Only the MAR assumption is briefly mentioned in the introduction and in the simulation study, but not elaborated on. The missing imputation mechanism and the assumptions should also be addressed in much more detail. What is the case, if the data are MNAR?

Response:

Three assumptions are discussed in the context of missing imputation, which also forms the basis of later simulations: missing completely at random (MCAR), missing at random (MAR), and missing not at random (MNAR) (Rubin, 1987; Graham et al., 2003; and Von Buuren, 2018).

Both the MCAR and MAR assumptions are mentioned and elaborated in the introduction. (Little, 1992; Chen and Ibrahim, 2013; Hasan et al., 2017; Little, 2021; and Isabella Sayers et al., 2022). In addition, MCAR assumptions and mechanisms are mentioned in the Materials and Methods section.

5.4) MAR: MAR is assumed in the simulation study (p. 8). Roughly speaking, if a missing value of any variable in the dataset depends on observed values of other variables in the dataset, then it is defined as MAR. I wonder how MAR is realized. Since X1 is complete, the data in X2 and its missingness should related to X1, i.e. the higher X1, the more missing values in X2. In the simulation, however, X1 and X2 are simulated independently of each other, and the positions of the missing values are randomized. This is more in favor of MCAR. These issues would need further clarification in the revision. The variance inflation factor (VIF), which also takes into account the sample size and the root mean square error, would be a more appropriate measure of multiple collinearities than the Pearson correlation.

Response:

We have made a mistake in saying that we assumed MAR. Instead, MCAR was assumed in this simulation study. The missing values in X_2 were not related to X_1. In the simulation, X_1 and X_2 were simulated independently of each other. The positions of the missing values in X_2 were randomized, they were replaced with the series mean of the entire data set in X_2.

Multicollinearity can be tested based on VIF and Pearson correlation coefficient. In this research, we used the Pearson correlation coefficient because it was easier to use the values of the independent variables X_1 and X_2 to determine the correlation coefficient without the need to determine the value of the dependent variable Y first (Glass and Hopkins, 1984; Howell, 2007). However, if VIF is obtained, it is necessary to know the value of the dependent variable in multiple regression analysis.

5.5) SRS estimator: The ratio estimator introduced on page 10 needs further explanation, especially in the context of missing values and missing value imputation. The presentation of Eq. 4 is somewhat confusing. In my view less confusing is the definition by Lu (2013): u_YSRS = mean_y_srs*(u_x/mean_xsrs), where u_x is replaced by mean_x_in (Eq. 8). For me it is not quite clear what is meant by "mean_x_in is the perfect sample mean of independent variable X2". Common taxonomies in missing value statistics with a missing value variable R, with R=1 (complete) and R=0 (missing) may be helpful in the statistical derivations.

Response:

The reason that we introduced the ratio estimator in Eq. (4) on page 15 was because it was used to derive Eq. (10) and (11) that we used in this study (see in adjusted manuscript).

A further explanation of the ratio estimator is seen above in Eq. (4).

$\bar{x}_{in}$ is the sample mean of the independent variable X_2 with the complete value (see below in Eq. (8) on page 16).

$\bar{x}_{ir}$ is the sample mean of the independent variable X_2 without accounting for the missing value.

5.6) Conclusions: It is relatively difficult to draw practical implications from the simulation studies, as certain methods of multiple imputation are better or worse depending on the level of error variance and the measures (mean absolute percentage error, mean square error), comparable to the heterogeneous results of Jomprapan (2012). Furthermore, I wonder what a researcher should do when he or she has a linear regression with more than 2 predictors. The practical implications should be made clear. In the statistical literature, missing imputation approaches are discussed for the dependent variables, for the covariates, and for both variables (e.g., Shao, 2013). It is often the case in applications that there are missing values in both the dependent variable and the covariates. The proposed approaches, which focus on the covariates, therefore appear to be of only limited use.

5.6.1) It is relatively difficult to draw practical implications from the simulation studies, as certain methods of multiple imputations are better or worse depending on the level of error variance and the measures (mean absolute percentage error, mean square error), comparable to the heterogeneous results of Jomprapan (2012).

Response:

Yes, our focus on the covariates makes out methods to be of limited use. However, we did not tackle both the cases of covariates and dependent variable because the amount of work would be inhibitive. As we have time, we will consider doing so in a more extensive manner and suggest that some other researchers may be interested in doing so as well. On drawing practical implications, it is not difficult to reap the benefits of our summary table that suggests the best imputation method based on sample size, error variance, and missing value percentage.

5.6.2) What a researcher should do when he or she has a linear regression with more than 2 predictors? The practical implications should be made clear.

Response:

In situations where there are more than two independent variables, such as three independent variables, we can also estimate the missing value imputation. It will be even more difficult. There will be many more situations depending on which independent

variables have missing values, for example, if only one independent variable is missing, there are three situations; or if two independent variables are missing, there are three situation; or all three independent variables are missing, there is only one situation, as follows. There are a total of seven situations. The results should be similar to the situation with two independent variables. One practical implication for a hypothetical researcher is that he or she should focus on the independent variables because in a real situation, the chance that an independent variable of an experiment has some missing values is high. After all, the value of the independent variable at a particular time and place may not be attainable, constrained by the conditions of the experiment. In this research, two independent variables were used as a guideline for estimating the missing value imputation in multiple regression analysis with simple random sampling (SRS) and ranked set sampling (RSS) methods.

5.6.3) In the statistical literature missing imputation approaches are discussed for the dependent variables, for the covariates, and both variables (e.g., Shao, 2013). It is often the case in applications that there are missing values in both the dependent variable and the covariates. The proposed approaches, which focus on the covariates, therefore appear to be of only limited use.

Response:

The response to this comment is Response 5.6.1) above.

5.7) References

Chen Q., Ibrahim J.G. (2013). A note on the relationships between multiple imputation, maximum likelihood and fully bayesian methods for missing responses in linear regression models. Statistics and its Interface, 6(3), 315-324.

Hasan H., Ahmad S., Osman B.M., Sapri S., Othman N. (2017). A comparison of model-based imputation methods for handling missing predictor values in a linear regression model: A simulation study. AIP Conference Proceedings, 1870, art. no. 060003.

Little R.J. (2021). Missing data assumptions. Annual Review of Statistics and Its Application, 8, pp. 89-107.

Little R.J. (1992). Regression with missing X's: a review. J. Am. Stat. Assoc. 87:1227-3.

Rubin D.B. (1987). Multiple Imputation for Nonresponse in Surveys. New York: Wiley

Shao, J. (2013). Estimation and imputation in linear regression with missing values in both response and covariate. Statistics and its Interface, 6(3), pp. 361-368.

Song L., Guo, G. (2024). Full Information Multiple Imputation for Linear Regression Model with Missing Response Variable. Journal of Applied Mathematics, 54(1), pp. 77-81.

Thongsri T., Samart K. (2022). Development of Imputation Methods for Missing Data in Multiple Linear Regression Analysis. Lobachevskii Journal of Mathematics, 43(11), pp. 3390-3399.

Von Buuren, S. (2018). Flexible Imputation of Missing Data. New York: Chapman & Hall.

Yang X., Belin T.R., Boscardin W.J. (2005). Imputation and variable selection in linear regression models with missing covariates. Biometrics, 61(2), pp. 498-506.

Response:

I have added these references in my manuscript as suggested.
