# Supplementary material for: New adjusted missing value imputation in multiple regression with simple random sampling and rank set sampling methods
Source: PLoS One. 2025 Mar 17;20(3):e0316641. doi: 10.1371/journal.pone.0316641 (PMC11913305; doi:10.1371/journal.pone.0316641)
Supplement: Attachment — Submitted filename: Response to Reviewers - Revised.pdf [file pone.0316641.s005.pdf]

## Response to Reviewers

# New adjusted missing value imputation in multiple regression with simple random sampling and rank set sampling methods

## Reviewers' Comments to the Responses

**Reviewer #1:** (No Response)

**Reviewer #2:** The revised manuscript focusses on the efficiency of four imputation methods: regression-ratio quartile1,3 (R-RQ1,3) imputation of Al-Omari, Jemain and Ibrahim; adjusted regression-chain ratio quartile1,3 (AR-CRQ1,3) imputation of Kadilar and Cinji; adjusted regression-multivariate ratio quatile1,3 (ARMRQ1,3) imputation of Feng, Ni, and Zou; and adjusted regression-multivariate chain ratio quartile1,3 (AR-MCRQ1,3) imputation of Lu for each simple random sampling (SRS) and rank set sampling (RSS). Simulation studies were conducted to investigate the performance of different methods using mean square error and mean absolute error.

I really appreciate the improvements the authors have made to the manuscript (e.g. discussion of MAR and MCAR). Thank you very much! Despite these improvements and the importance of the topic of missing imputation in linear regression, I cannot recommend the revised manuscript for publication in PlosOne for the following reasons:

1. MCAR: A strong limitation of the results is the assumption of MCAR in the simulation study. Listwise deletion or maximum likelihood estimation would be equally effective. The advantage of missing imputation over complete case analysis is that it avoids the reduction in sample size and diminishes the confidence intervals of the parameters, as mentioned in the manuscript. However, the simulation study would need to include at least one MCAR condition. It would also need to include a control condition with Listwise deletion. Overall, MCAR seems to be rather unrealistic for practical applications.

**Response:**

1.1 MCAR: A strong limitation of the results is the assumption of MCAR in the simulation study. Listwise deletion or maximum likelihood estimation would be equally effective.

Listwise deletion or maximum likelihood estimation may be effective. The authors would to present new methods that were researched from many journals using auxiliary variables.

The aforementioned studies inspired the authors to study the use of a ratio estimator that utilizes the 1$^{st}$ and 3$^{rd}$ quartiles of Al-0mari et al. (2009). The estimator was for estimating the missing values in multiple regression analysis with a simple random sampling (SRS). An efficient missing-value imputation method was the regression-ratio quartile1,3 imputation with SRS of Al-Omari, Jemain, and Ibrahim. This method was highly efficient. The authors propose three new adjusted methods for comparison: the adjusted regression-chain ratio quartile1,3 imputation with SRS of Kadilar and Cingi (2003), the adjusted regression-multivariate ratio quartile1,3 imputation with SRS of Feng, Ni, and Zou (1998), which provided highly efficient, and the adjusted regression-multivariate chain ratio quartile1,3 imputation with SRS of Lu (2013), which provided the highest efficiency.

In addition, the authors compare an estimator of the utilization ratio of the 1$^{st}$ and 3$^{rd}$ quartiles analyzed with a rank set sampling (RSS) (referenced in Paglinawan (2017) versus simple random sampling. It was found that the efficiencies of the utilization ratio estimators of the

1st and 3rd quartiles analyzed with RSS were higher than those analyzed with SRS. Interested in developing further, the authors propose four new adjusted methods for estimating missing values in regression analysis: the adjusted regression-ratio quartile1,3 imputation with RSS of Al-Omari, Gemain, and Ibrahim (2009); the adjusted regression-chain ratio quartile1,3 imputation with RSS of Kadilar and Cinji (2003); the adjusted regression-multivariate ratio quartile1,3 imputation with RSS of Feng, Ni, and Zou (1998); and the adjusted regression-multivariate chain ratio quartile1,3 imputation with RSS of Lu (2013).

However, I added the assumption of MCAR in the simulation study with the Listwise deletion estimation in all of Table 2-11 for SRS and Table 12-21 for RSS in the Result section. I also included the Listwise deletion in gold loss of Table 22-25 for SRS and RSS, and platinum loss of Table 26-29 for SRS and RSS in the Result section.

1.2 The advantage of missing imputation over complete case analysis is that it avoids the reduction in sample size and diminishes the confidence intervals of the parameters, as mentioned in the manuscript.

The authors avoided sample size reduction in sample size and diminished the confidence intervals of the parameters by increasing the sample sizes to very large sizes of 200 and 500. The authors also increased the percentages of missing values of 30 and 40%. Very large percentages of missing values are not good. We should collect new data because the replacement of missing values is not real data. The authors tried to process some values of the percentages of missing values of 50% and got the same result as the percentages of missing values of 30 and 40%. The authors omit the percentage of missing values of 50%.

1.3 However, the simulation study would need to include at least one MCAR condition. It would also need to include a control condition with Listwise deletion.

Most of the new literature reviews had not included the Listwise deletion at all. For example, the research of Al-Omari, Jemain, and Ibrahim (2009); Kadilar and Cinji (2003); Feng, Ni, and Zou (2014); and Lu (2015) for each simple random sampling (SRS) and rank set sampling (RSS).

Listwise deletion is the removal of records (rows) or columns with missing data from the analysis and the remaining data is analyzed. This method can be error-prone, as the pattern of data loss can be systematic, and eliminating records with missing data will lead to bias in the data. In addition, information in all other data regions will be lost because values in one region are lost. We may replace the missing values with some constant values, such as mean imputation, mean of nearby points, median of nearby points, etc. (Larose, 2005).

However, I added a control condition with Listwise deletion of all of Table 2-11 for SRS and Table 12-21 for RSS in the Result section. I also included the Listwise deletion in gold loss of Table 22-25 for SRS and RSS, and platinum loss of Table 26-29 for SRS and RSS in the Result section.

2. MAR: On page 6 we read: "Although it is easy to show that full-case analysis is unbiased and efficient when the missing at random (MAR) assumption is made, the methods above are still commonly used in practice for this setting". Strictly speaking, this statement only applies in the case of MCAR or the case of missing values only for the outcome variable. Under the assumption of MCAR, complete case analysis without missing imputation or full information maximum likelihood of missing values in the covariates might lead to bias.

**Response:**

2.1 This statement only applies in the case of MCAR or the case of missing values only for the outcome variable.

In some research, the estimation of missing values in covariates is also used, such as the research titled “Missing value estimation in multiple linear regression analysis” by Jomprapan, R. (2002); “Missing value estimation in multiple linear regression analysis. Journal of Development Administration; by Jomprapan R, Siripanich P. (2015) and “A comparison of model-based imputation methods for handling missing predictor values in a linear regression model: A simulation study” by Hasan et al. (2017). For the reason above, the authors applied in the case of MCAR or the case of missing values only for the covariate variable.

2.2 Under the assumption of MCAR, complete case analysis without missing imputation or full information maximum likelihood of missing values in the covariates might lead to bias.

We just assumed that the maximum likelihood estimator (MLE) of missing values in the covariates may be biased, but we have not seen any mention that the above statement is true.

3. Generalizability of the results: The results of the simulation study ultimately relate to a regression analysis with 2 covariates and MCAR. This is a very limited scope of the approach.

**Response:**

3.1 The results of the simulation study ultimately relate to a regression analysis with 2 covariates and MCAR. This is a very limited scope of the approach.

The authors proposed 7 new methods using 2 auxiliary variables in the covariate for simple random sampling and rank set sampling. Sample sizes provided 8 values, the proportions of missing values provided 6 values, and missing value estimation methods provided 8 values which was quite a wide scope in multiple regression analysis with 2 covariates and MCAR. There were 8x6x8 = 384 scenarios in total for each error variance of the sampling method. The authors studied 2 covariates as a basis for those who interest in further the developers for other covariates in SRS and RSS. If there is enough time, we can expand from 2 covariates to 3 or more. The results should be similar to the authors.

In addition, the authors compared a better sampling method between SRS and RSS estimators based on sample sizes, and error variances. The result showed that the RSS estimators provided smaller MSE and MAPE than the SRS estimators. Therefore, the RSS estimators were more efficient than the SRS estimators.

4. Sample size (Table 1). It makes little sense to include very small sample sizes of 20 or 40 with 2 covariates as a condition in the simulation. Higher sample sizes of 1000 and more and higher proportions of missing values of 40-50% would also be of interest. Since MCAR is assumed, the question is whether a proportion of 5-10% missing values significantly increases the power of the statistical tests, especially for larger sample sizes above 100. I fear that the differences between the methods will diminish with larger sample sizes and smaller proportions of missing values.

**Response:**

4.1 It makes little sense to include very small sample sizes of 20 or 40 with 2 covariates as a condition in the simulation. Higher sample sizes of 1000 and more and higher proportions of missing values of 40-50% would also be of interest.

I added the higher sample sizes of 200 and 500 all of Table 2-11 for SRS and Table 12-21 for RSS in the Result section. In addition, I included the proportions of missing values of 30 and 40% all of Table 2-11 for SRS and Table 12-21 for RSS in the Result section.

The sample size is much larger. It will take a very long time to process. The authors have tried processing some values of a sample size of 1,000 and got the same result as sample sizes of 200 and 500. The authors did not process the data in the case of a sample size of 1,000. Therefore, the authors request to increase only the sample sizes of 200 and 500.

In the case of a missing value of 50%, the authors believe that the missing value is too much. The missing value should not be replaced with statistics because the value that replaces the missing value is not real data. In addition, the data analysis may be highly error. Therefore, the authors request to increase only the missing values of 30 and 40%.

4.2 Since MCAR is assumed, the question is whether a proportion of 5-10% missing values significantly increases the power of the statistical tests, especially for larger sample sizes above 100. I fear that the differences between the methods will diminish with larger sample sizes and smaller proportions of missing values.

The differences between the methods were not diminished with larger sample sizes and smaller proportions of missing values of Table 2-11 for SRS and Table 12-21 for RSS. The results of the large sample size and small proportions of missing values are mostly consistent with small and middle sample sizes.

5. Alternatives: Alternatives to the proposed approaches are discussed but not considered in the analysis (e.g., FIML, complete case analysis), and results remain idiosyncratic. In statistics as well, it is important not only to replicate the results of other authors' simulation studies but also to compare the results with the results of other studies with the sample design of one's own study.

**Response:**

5.1 Alternatives to the proposed approaches are discussed but not considered in the analysis (e.g., fully informative multiple imputation (FIML), complete case analysis), and results remain idiosyncratic.

The authors did not simulate the dependent variable from the beginning. The dependent variable (Y) was created from the multiple regression equation of the two independent variables ($X_1$ and $X_2$). As for all data analysis, the dependent variable must be created from the multiple regression equation of the two independent variables as above. The dependent variable has the same value. The authors could not find the mean square error and the mean absolute percentage error; or both values are equal to zero. Therefore, the authors will not present all data analysis for the reasons mentioned above.

The results were not much different. For both SRS and RSS, the AR-MCRQ1 was the best method for missing value imputation in multiple regression analysis, followed by the AR-MCRQ3 in Table 31 of the Conclusions section and Abstract.

5.2 In statistics as well, it is important not only to replicate the results of other authors' simulation studies but also to compare the results with the results of other studies with the sample design of one's own study.

There are the comparisons of other studies with the sample design of one’s own study (SRS and RSS) in the Discussions section as follows:

The results of the study show that for a small error variance, AR-MRQ1 with SRS achieved the minimum mean square error. However, for a large error variance, AR-MCRQ3 achieved the minimum mean square error. For all error variances in mean absolute percentage error, AR-MCRQ1 achieved the minimum mean absolute percentage error. The results of this study were similar to those reported in [14,15]. In those papers, the proposed estimator was a multivariate chain ratio (MCR) estimator and a regression estimator that used a linear combination of two auxiliary variables. The MCR estimator and regression estimator that used the two auxiliary variables were equally highly effective, followed by the traditional multivariate ratio (MR) estimator and the regression estimator that used the two auxiliary variables. This study was also consistent with [11] in that the chain ratio (CR) estimator with SRS was more efficient than the traditional ratio estimator under certain conditions. Our results may be contrasted to [22] which reported that the regression-ratio Q1 (R-RQ1) estimator was the most efficient. This discrepancy was because the study did not include AR-CRQ1,3, AR-MRQ1,3, and AR-MCRQ1,3 estimators in the test.

Moreover, the RSS estimators provided smaller mean square error and mean absolute percentage error than the SRS estimators for the same error variances, sample sizes, and missing value percentages. Therefore, the RSS estimators were more efficient than the SRS estimators in Table 32 of the Conclusions section. Our results agree well with those reported by [3-5,13]: the RSS estimators were more efficient than the SRS estimators for the same quartile, coefficient of correlation, and sample size.
