# Supplementary material for: New adjusted missing value imputation in multiple regression with simple random sampling and rank set sampling methods
Source: PLoS One. 2025 Mar 17;20(3):e0316641. doi: 10.1371/journal.pone.0316641 (PMC11913305; doi:10.1371/journal.pone.0316641)
Supplement: Attachment — Submitted filename: Response to Reviewers - Revised 3.pdf [file pone.0316641.s006.pdf]

Response to Reviewers

New adjusted missing value imputation in multiple regression with simple random sampling and rank set sampling methods

Reviewers' Comments to the Responses

Reviewer #3: Thank you for the opportunity to review your paper on New Adjusted Missing Value Imputation in Multiple Regression with Simple Random Sampling and Rank Set Sampling Methods. This paper makes a noteworthy contribution to statistical methodologies for handling missing data in regression analysis, an area of significant importance for researchers facing incomplete datasets. I am pleased to recommend this paper for publication, as it presents a valuable contribution to the field of statistical analysis in handling missing data within regression models. Congratulations on this well-executed and impactful research!

Response: Thank you for your comment.

Reviewer #4: The authors are required to revise all in-text citations to ensure that they are consistent throughout the paper and adhere to the PLOS ONE format. Additionally, the quality of the English writing must be improved by having a native speaker proofread this work.

Response: I've revised all in-text citations and the quality of my English writing as suggested.
